# 3D hESC exosomes enriched with miR-6766-3p ameliorates liver fibrosis by attenuating activated stellate cells through targeting the TGFβRII-SMADS pathway

**DOI:** 10.1186/s12951-021-01138-2

**Published:** 2021-12-20

**Authors:** Ning Wang, Xiajing Li, Zhiyong Zhong, Yaqi Qiu, Shoupei Liu, Haibin Wu, Xianglian Tang, Chuxin Chen, Yingjie Fu, Qicong Chen, Tingting Guo, Jinsong Li, Shuai Zhang, Mark A. Zern, Keqiang Ma, Bailin Wang, Yimeng Ou, Weili Gu, Jie Cao, Honglin Chen, Yuyou Duan

**Affiliations:** 1 School of Biomedical Sciences and Engineering, Guangzhou International Campus, South China University of Technology, Guangzhou, 510006 People’s Republic of China; 2 Laboratory of Stem Cells and Translational Medicine, Institutes for Life Sciences and School of Medicine, South China University of Technology, No.382 Waihuan East Road, Suite 406, Higher Education Mega Center, Guangzhou, 510006 People’s Republic of China; 3 School of Medicine, South China University of Technology, Guangzhou, 510180 People’s Republic of China; 4 School of Biology and Biological Engineering, South China University of Technology, Guangzhou, 510006 People’s Republic of China; 5 State Key Laboratory of Cell Biology, Shanghai Key Laboratory of Molecular Andrology, Shanghai Institute of Biochemistry and Cell Biology, Center for Excellence in Molecular Cell Science, Chinese Academy of Sciences, Shanghai, 200031 China; 6 University of Chinese Academy of Sciences, Shanghai, 200031 China; 7 Department of Gastroenterology and Hepatology, Guangzhou Digestive Disease Center, Guangzhou First People’s Hospital, School of Medicine, South China University of Technology, No.1 Panfu Road, Guangzhou, 510180 People’s Republic of China; 8 Department of Internal Medicine, University of California Davis Medical Center, Sacramento, CA 95817 USA; 9 Department of Hepatobiliary Pancreatic Surgery, Huadu District People’s Hospital of Guangzhou, Guangzhou, 510800 People’s Republic of China; 10 Department of General Surgery, Guangzhou Red Cross Hospital, Jinan University, Guangzhou, 510220 People’s Republic of China; 11 Department of General Surgery, The First Affiliated Hospital of Guangdong Pharmaceutical University, Guangzhou, 510080 People’s Republic of China; 12 Department of General Surgery, Guangzhou Digestive Disease Center, Guangzhou First People’s Hospital, School of Medicine, South China University of Technology, No.1 Panfu Road, Guangzhou, 510180 People’s Republic of China; 13 National Engineering Research Center for Tissue Restoration and Reconstruction, South China University of Technology, Guangzhou, 510006 People’s Republic of China; 14 Key Laboratory of Biomedical Engineering of Guangdong Province, South China University of Technology, Guangzhou, 510006 People’s Republic of China; 15 Key Laboratory of Biomedical Materials and Engineering of the Ministry of Education, South China University of Technology, Guangzhou, 510006 People’s Republic of China; 16 Innovation Center for Tissue Restoration and Reconstruction, South China University of Technology, Guangzhou, 510006 People’s Republic of China

**Keywords:** Human embryonic stem cells, Cell spheroids, Exosomes, Liver fibrosis, microRNAs, TGFβRII-SMADS pathway

## Abstract

**Background:**

Exosomes secreted from stem cells exerted salutary effects on the fibrotic liver. Herein, the roles of exosomes derived from human embryonic stem cell (hESC) in anti-fibrosis were extensively investigated. Compared with two-dimensional (2D) culture, the clinical and biological relevance of three-dimensional (3D) cell spheroids were greater because of their higher regeneration potential since they behave more like cells in vivo. In our study, exosomes derived from 3D human embryonic stem cells (hESC) spheroids and the monolayer (2D) hESCs were collected and compared the therapeutic potential for fibrotic liver in vitro and in vivo.

**Results:**

In vitro, PKH26 labeled-hESC-Exosomes were shown to be internalized and integrated into TGFβ-activated-LX2 cells, and reduced the expression of profibrogenic markers, thereby regulating cellular phenotypes. TPEF imaging indicated that PKH26-labeled-3D-hESC-Exsomes possessed an enhanced capacity to accumulate in the livers and exhibited more dramatic therapeutic potential in the injured livers of fibrosis mouse model. 3D-hESC-Exosomes decreased profibrogenic markers and liver injury markers, and improved the level of liver functioning proteins, eventually restoring liver function of fibrosis mice. miRNA array revealed a significant enrichment of miR-6766-3p in 3D-hESC-Exosomes, moreover, bioinformatics and dual luciferase reporter assay identified and confirmed the TGFβRII gene as the target of miR-6766-3p. Furthermore, the delivery of miR-6766-3p into activated-LX2 cells decreased cell proliferation, chemotaxis and profibrotic effects, and further investigation demonstrated that the expression of target gene TGFβRII and its downstream SMADs proteins, especially phosphorylated protein p-SMAD2/3 was also notably down-regulated by miR-6766-3p. These findings unveiled that miR-6766-3p in 3D-hESC-Exosomes inactivated SMADs signaling by inhibiting TGFβRII expression, consequently attenuating stellate cell activation and suppressing liver fibrosis.

**Conclusions:**

Our results showed that miR-6766-3p in the 3D-hESC-Exosomes inactivates smads signaling by restraining TGFβRII expression, attenuated LX2 cell activation and suppressed liver fibrosis, suggesting that 3D-hESC-Exosome enriched-miR-6766-3p is a novel anti-fibrotic therapeutics for treating chronic liver disease. These results also proposed a significant strategy that 3D-Exo could be used as natural nanoparticles to rescue liver injury via delivering antifibrotic miR-6766-3p.

**Graphical Abstract:**

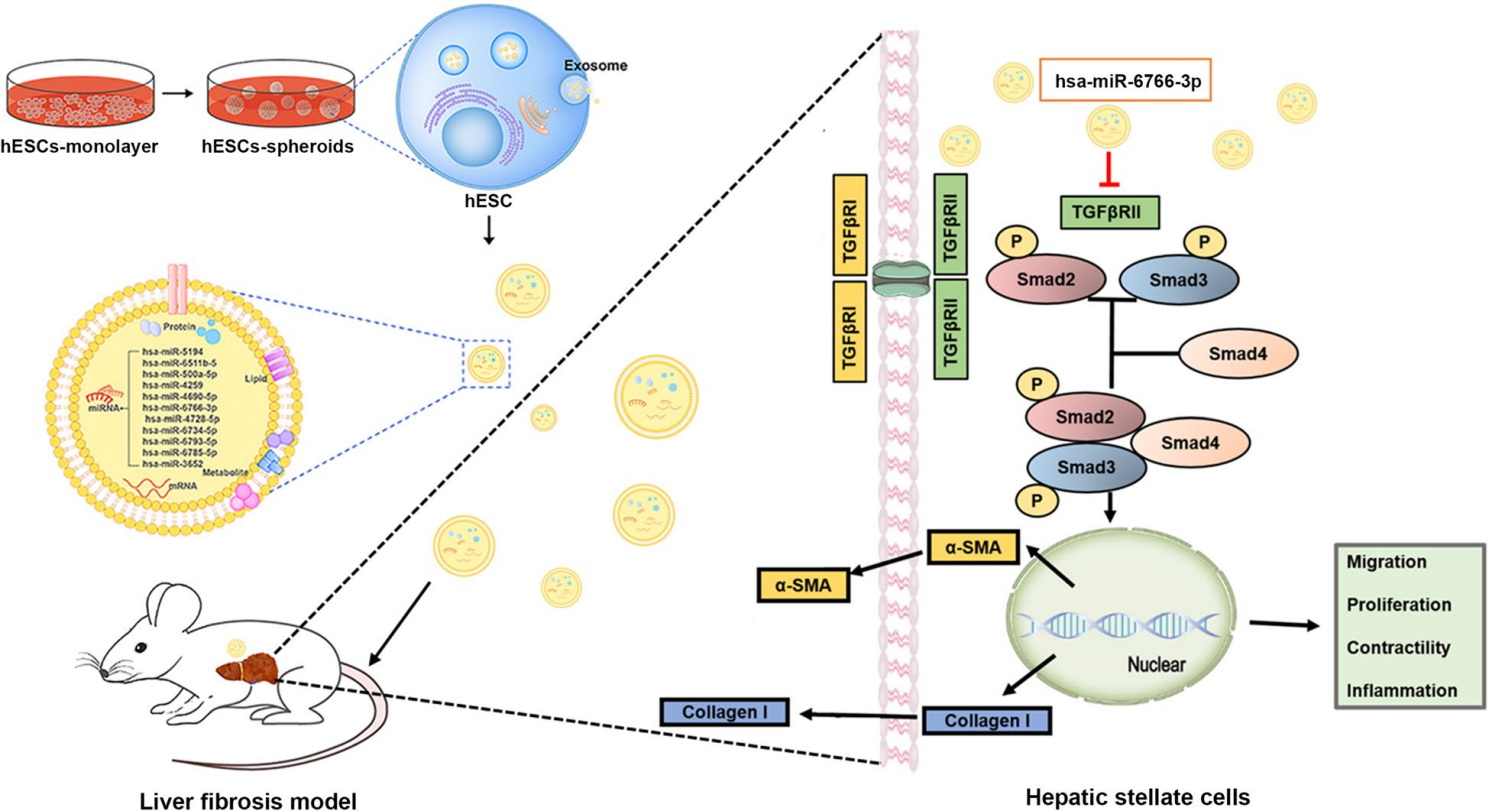

**Supplementary Information:**

The online version contains supplementary material available at 10.1186/s12951-021-01138-2.

## Background

Liver fibrosis, which is one of the major risk factors for the development of liver cancer, leads to the requirement of liver transplantation when it leads to advanced cirrhosis [1]. However, the vast majority of liver disease patients with advanced cirrhosis are unable to undergo transplantation due to complications. Moreover, even in patients, who are appropriate liver transplant recipients, transplantation is still plagued by limited donor supplies, high costs, and the need for chronic immunosuppressive therapy [2]. Therefore, liver regeneration therapy has emerged as an attractive therapeutic strategy to meet the increasing need for liver transplantation [3].

It is acknowledged that the imbalance of the homeostasis mechanism, which controls the formation and degradation of extracellular matrix (ECM), causes a series of pathological changes in the ECM of liver tissue [4, 5]. Indeed, continuous necrosis and necrosis-associated inflammation trigger excessive ECM synthesis and reduced degradation, resulting in liver fibrosis [6]. Eventually, liver fibrosis can progress into cirrhosis [7]. Liver injury induced by fibrosis or cirrhosis involves apoptosis of liver parenchymal cells and activation of hepatic stellate cells, which cannot be reversed by the body itself [8, 9]. As a result, liver repair with exogenous stem cells provides an alternative for liver organ or cell transplantation [10]. Mesenchymal stem cell (MSC) therapy has been utilized in clinical trials of liver disease treatment [11]. Moreover, it has been shown that pluripotent stem cells (PSC), including human embryonic stem cells (hESCs) and human induced pluripotent stem cells (hiPSC), exert great potential for liver regeneration because of their unparalleled ability to differentiate [12, 13]; however, the risk to form teratomas would limit the transplantation of PSC-derived cells [14]. Additionally, some issues still exist in regard to the application of both MSC and ESC in treating fibrotic livers, such as cell retention rate, and survival difficulties [15]. Taken together, there is a strong demand for enhancing the regenerative ability of PSC while avoiding the possible risks related to cell transplantation [16]. Importantly, accumulating evidence demonstrate that a wide range of immunosuppressive and paracrine factors generated by stem cells display efficient effects in reversing fibrosis and enhancing liver cell function [17]. In an attempt to enhance the effects of stem cell therapeutics, our group has suggested that stem cell based-exosomes could provide therapeutic effects in cell-free treatment of liver diseases [18].

Donor-derived exosomes, which are vesicles released after fusion of multi-vesicleand plasma membrane endocytosis, contain non-coding small RNAs and other biomolecules (such as proteins, lipids, and mRNAs) that can stimulate T cell-like responses in target cells [19, 20]. Some components inside exosomes have been shown to enhance tissue regeneration and regulate endogenous repair [19]. Moreover, compared with 2D culture, the clinical and biological relevance of 3D cell spheroids are greater because of their enhanced regeneration potential [21]. In the present study, we investigated the roles of 2D hESC-Exosomes (2D-Exo) and 3D hESC-Exosomes (3D-Exo) played in liver repair using a liver fibrosis mouse model. We found that 2D-Exo and 3D-Exo were able to both improve the function of liver parenchymal cells and inhibit the activation of hepatic stellate cells, thus enhancing endogenous liver repair. Furthermore, we demonstrated that the underlying mechanisms of their function involved in part, the transfer of hESC-specific microRNA. Overall, our results indicated that hESC-derived exosomes might be a new cell-free system that can effectively enhance endogenous liver repair after injury.

## Results

### Characterization of 2D and 3D hESC-derived Exosome

**Fig. 1:**
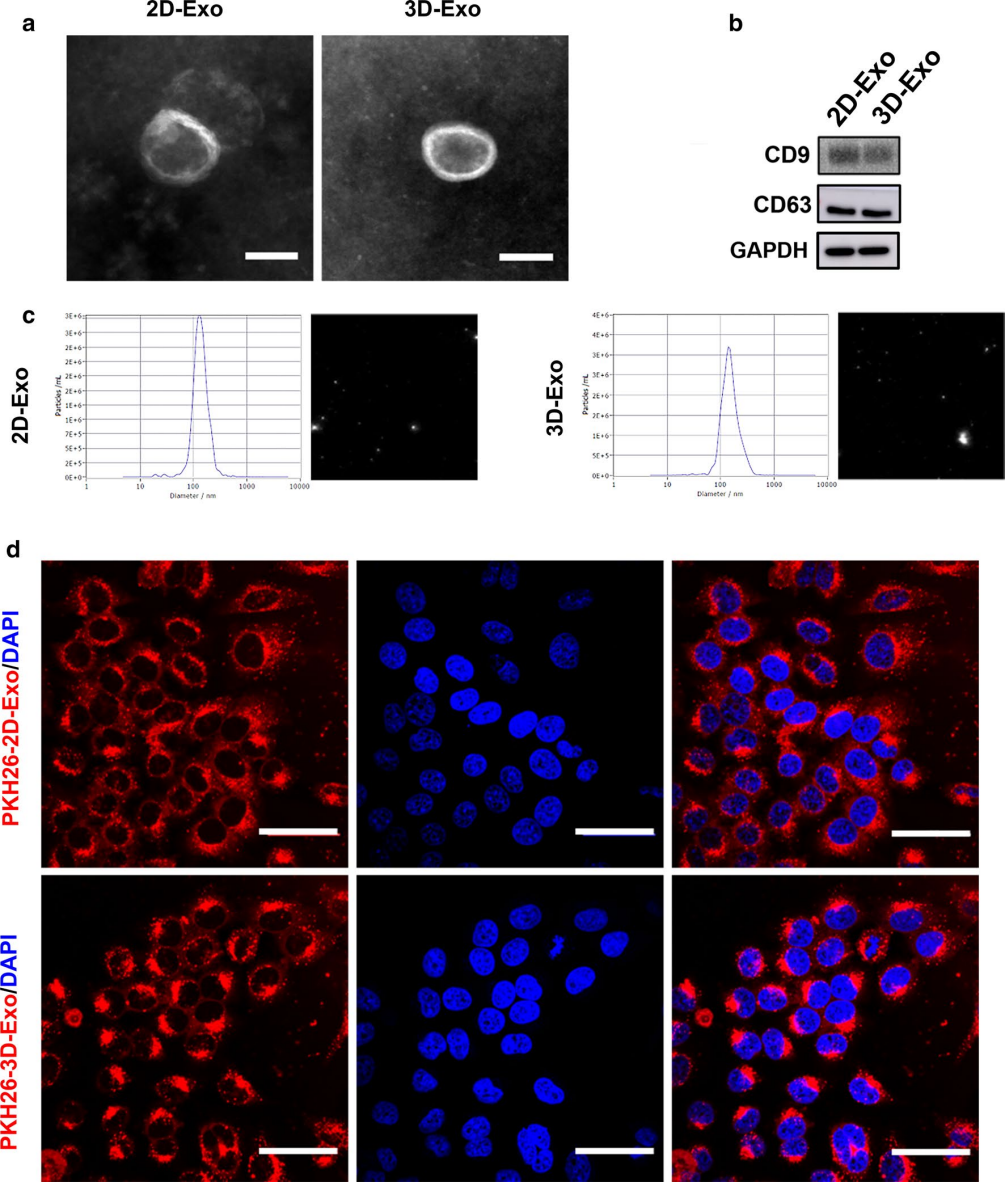
Characterization of hESC-Exosomes. **a** TEM images of 2D-Exo and 3D-Exo. Original magnification, 40,000×, Scale bar, 100 nm. **b** Western blot analysis of CD9 and CD63 in 2D-Exo and 3D-Exo. **c** Size distribution of 2D-Exo and 3D-Exo analyzed by NTA in scatter mode. **d** CLSM images of 2D-Exo and 3D-Exo labeled with PKH26. Red: PKH26-Exo. Blue: DAPI. Original magnification, 40×, Scale bar, 50 μm

It has been reported that 3D culture can maintain cell stemness [22–25], thus, we passed hESCs from 2D culture into ultralow attachment culture plates to form hESC spheroids to determine whether the stemness of hESCs was improved by 3D culture. The morphology of hESCs in a monolayer (2D) culture, as demonstrated in Additional file 1: Fig. S1a, is a classic colony shape. When hESCs were cultured in suspension, they formed compact spheroids, and the average diameter of hESC spheroids was around 100–200 μm (Additional file 1: Fig. S1a). The spheroids with these sizes highly expressed stemness-related proteins, SOX2, OCT4, NANOG and TRA-1–81 (Additional file 1: Figs. S1b–d and S2d), even exhibited slightly enhanced stemness when cultured as 3D spheroids. However, the major barriers to the possible transplantation of hESCs into patients are ethical issues and tumorigenic potential. The transplantation of 2D- and 3D-hESCs both triggered teratomas (Additional file 1: Fig. S2a, b). Unlike hESCs, no teratomas were formed after injection of 2D-Exo or 3D-Exo in immunodeficient mice (Additional file 1: Fig. S2c). Consistently, the results obtained by flow cytometry revealed that both 2D-hESCs and 3D-hESCs strongly expressed a tumorigenic marker, TRA-1–81, while TRA-1–81 expression were almost undetected in exosomes derived from both two groups (Additional file 1: Fig. S2d, e). These data indicated that hESC-derived exosomes could be harnessed as a new and alternative therapeutics for the treatment of many human diseases including liver fibrosis. As visualized by transmission electron microscopy (TEM), both exosomes appeared as round and cup-shaped vesicles of approximately 120–140 nm in diameter, exhibiting clear membrane structures (Fig. 1a). Flow cytometry results showed that both 2D-Exo and 3D-Exo contained homogenous surface marker SSEA4, demonstrating they were derived from hESCs (Additional file 1: Fig. S1e). In addition, Western blot results confirmed that 3D-Exo had similar expression levels of exosome surface markers (CD9 and CD63) as 2D-Exo did (Fig. 1b). The diameter of the 3D-Exo was also similar to those of 2D-Exo as demonstrated by nanoparticle tracking analysis (NTA) (Fig. 1c). Furthermore, exosomes derived from 2D-hESCs and 3D-hESCs were labeled with PKH26, and labeled Exos were used to treat LX2 cells and internalized by LX2 cells. Cells were then stained with DAPI and examined by immunofluorescence, and all the internalized exosomes displayed remarkable fluorescent signals, distributed around the nucleus (Fig. 1d). Therefore, PKH26, an efficient fluorescent dye, was utilized for labeling and tracking exosomes in this study.

**Fig. 2:**
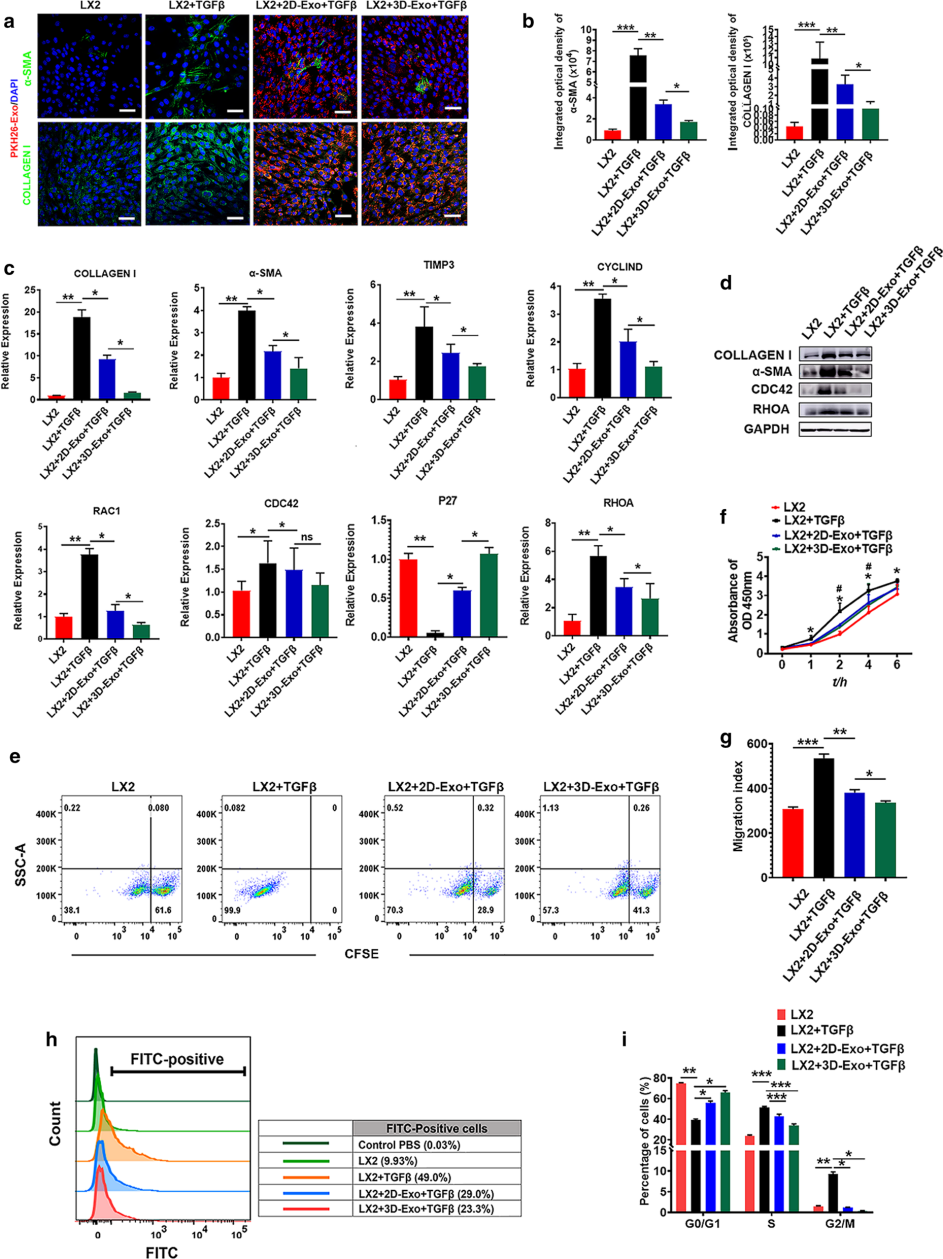
hESC-Exosomes internalized by LX2 cells reduced profibrogenic phenotype. Representative confocal microphotographs of TGFβ-treated LX2 cells exposed to PKH26-labeled 2D-Exo and 3D-Exo (red) and EV-free supernatant for 24 h. **a** 2D-Exo and 3D-Exo inhibited the expression of COLLAGEN I and α-SMA in activated LX2 cells. The colors of green, red, and blue represent the immunostainings for COLLAGEN I/α-SMA, PKH26 and nuclei, respectively (Original magnification, 20×, Scale bar, 40 μm). **b** Quantification of integrated optical density of COLLAGEN I and α-SMA in **a**. **c** Quantification of COLLAGEN I, α-SMA, TIMP3, CYCLIND, RAC1, CDC42, P27, and RHOA by qPCR. **d** Representative Western blot analyses of markers COLLAGEN I, α-SMA, CDC42, and RHOA in LX2 cells primed with TGFβ and exposed to 2D-Exo and 3D-Exo for 24 h. GAPDH was used as a loading control. **e** Proliferation (as measured by the dilution of carboxyfluorescein succinimidyl ester (CFSE)) of LX2 cells and activated LX2 cells with different treatments. **f** The proliferative activity of LX2 cells in each group were measured by CCK-8 assay at 1 h, 2 h, 4 h and 6 h after treatment. **g** Wound recovery rates of LX2 cells with different treatment, modeled by cell scratch assays (The images of cell scratch assays were provided in Additional file 1.). **h** Fluo-3 AM fluorescent probes were used to detect the concentration of calcium ions in LX2 cells of different treatment groups. **i** Quantification of cell cycle analysis by flow cytometry which was used to measure the growth of LX2 cells and activated LX2 cells exposed to 2D-Exo or 3D-Exo (Flow cytometry analysis used to determine the stages of cell cycle was provided in Additional file 1). Data represent the mean ± SEM. *P < 0.05, **P < 0.01, and ***P < 0.001

### 3D-hESC-derived Exosomes internalized by LX2 cells reduced the expression of profibrogenic markers

Subsequently, we compared the effects of exosomes derived from 2D-hESCs with those of 3D-hESC-Exosomes on the proliferation, migration, and function of TGFβ-activated LX2 cells in vitro. As expected, 2D-Exo and 3D-Exo fluorescently labeled with PKH26 were internalized by LX2 cells after 6 h of PKH26 exposure (Additional file 1: Fig. S3a). Confocal microscopy imaging detected that PKH26^+^ 2D-Exo and 3D-Exo were in the cytosol of LX2 cells, particularly localized in the perinuclear area (Additional file 1: Fig. S3a), suggesting that hESC-Exosomes taken up by LX2 cells might participate in mediating the biological processes of LX2 cells. To investigate whether hESC-Exosomes have potentially effects on the regulation of profibrogenic genes, we performed a gene expression analysis focused on several well-described markers of LX2 cell activation in the presence or absence of labeled-2D-Exo and 3D-Exo. First, immunofluorescence staining of profibrogenic markers, such as COLLAGEN I and α-SMA, were performed to evaluate the activation of LX2 cells, the results revealed that approximately half of LX2 cells expressed COLLAGEN I and α-SMA after TGFβ treatment, indicating that these cells became active myofibroblast-like cells (Fig. 2a, b). Interestingly, under highly efficient internalization shown by PKH26, we noticed that extremely low number of positive cells for COLLAGEN I and α-SMA in activated LX2 treated with 3D-Exo, indicating that 3D-Exo significantly prevented the trend to some extent through down-regulating the expression of these two proteins compared to 2D-Exo (Fig. 2a, b and Additional file 1: Fig. S3b, c). qRT-PCR results further showed the genes of COLLAGEN I and α-SMA were significantly increased in activated LX2 cells after TGFβ induction, while their expression level was strongly decreased with 3D-Exo treatment (Fig. 2c). In agreement, similar results were obtained by Western blot analysis (Fig. 2d, Additional file 1: Fig. S3d). This phenomenon also occurred to tissue inhibitor metalloproteinases 3 (TIMP3), the antagonist of matrix metalloproteinases (MMPs) which decompose the collagens, is usually concurrent with collagens in the livers during the progression of fibrosis. The expression of TIMP3 was in line with those of Collagen I either in activated LX2 or in the treatment of activated LX2 with 3D-Exo (Fig. 2c). These results demonstrated that 3D-Exo might play a vital role in posttranscriptional regulation of genes associated with profibrogenic TGFβ-dependent activation of LX2 cells.

**Fig. 3:**
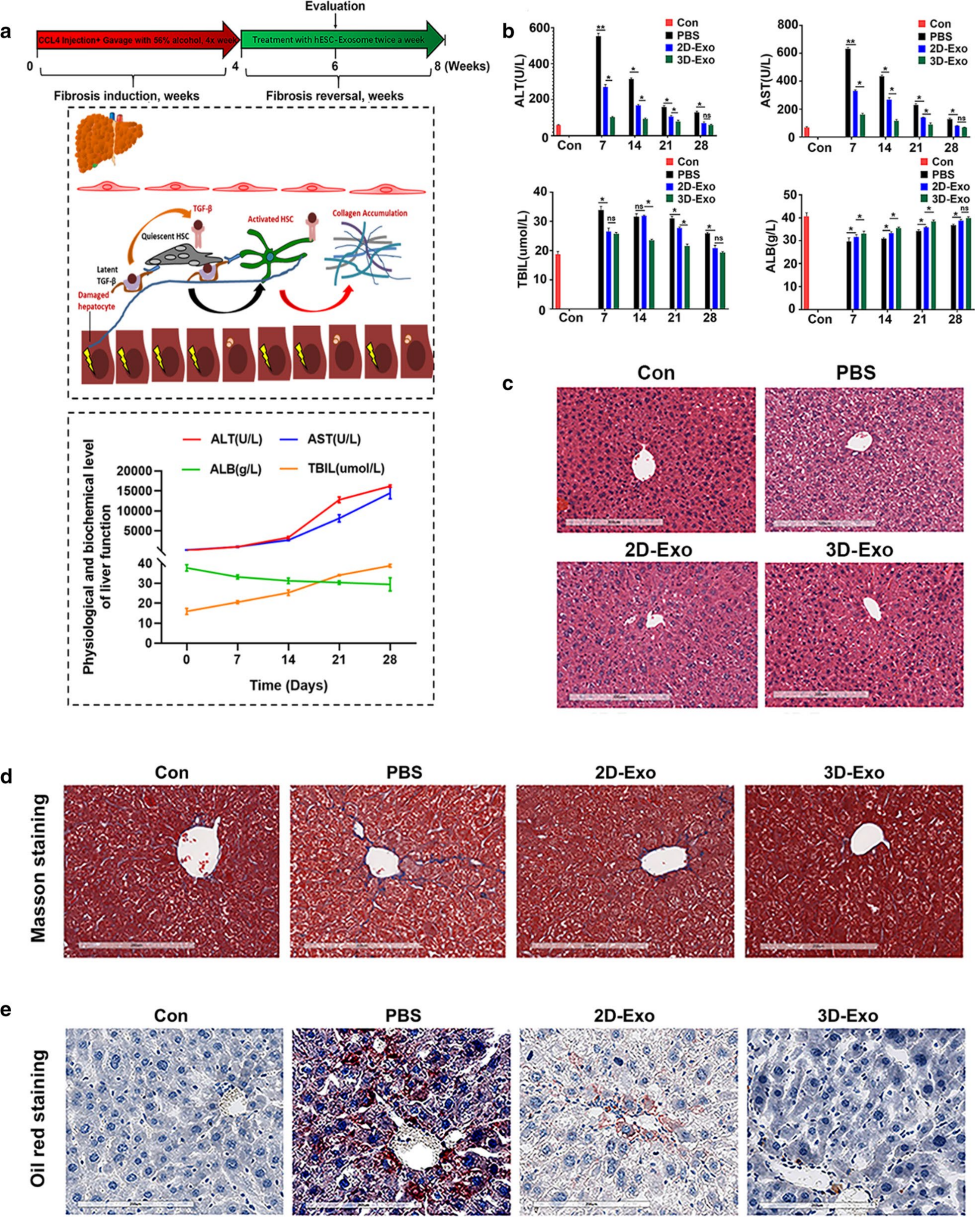
3D-Exo and 2D-Exo limited the progression of liver fibrosis and promoted liver function recovery in fibrotic mouse model. ICR mice were treated with CCl_4_ intravenously and 56% alcohol gavage for 4 weeks. During the 4th to 8th week, PBS, 2D-Exo and 3D-Exo were injected intravenously, respectively. **a** Schematic diagram of treatments in mice. The curves of liver function in fibrotic mouse model showed gradual deterioration of liver function. **b** The serum liver function indexes of mice treated with exosomes were measured with corresponding test kit. **c** HE staining of livers with different treatment groups showed that the deformed and necrotic liver cells decreased and the degree of inflammatory infiltration was reduced after exosome treatment, especially in 3D-Exo-treated group. (Original magnification, 20×, Scale bar, 200 μm). **d** Liver images of normal mice (con), and the mice injected with PBS, 2D-Exo or 3D-Exo. Masson staining showed that liver fibrosis was reduced in 2D-Exo- and 3D-Exo-treated mice compared with PBS-treated mice (Original magnification, 20×, Scale bar, 200 μm). **e** The oil red staining of liver in normal mice and treated mice was observed, and the content of lipid droplets in 3D-Exo-treated mice was significantly reduced. (Original magnification, 20×, Scale bar, 200 μm) Data represent the mean ± SEM. *P < 0.05, **P < 0.01, and ***P < 0.001

In addition to regulating profibrogenic genes, 3D-Exo also significantly reduced gene expression of the common markers of cell proliferation (CYCLIND, RAC1 and CDC42) in activated LX2 cells (Fig. 2c, d and Additional file 1: Fig. S3d), which are usually upregulated in activated stellate cells during liver fibrosis. In contrast, P27, an antagonist of cell cycle, strongly decreased in the proliferating activated LX2 cells, were significantly up-regulated in TGFβ-treated LX2 cells after treatment with 3D-Exo (Fig. 2c). It demonstrated that the proliferation of activated LX2 cells was seriously inhibited. This result was consistent with that the expressions of cell cycling markers CYCLIND, RAC1 and CDC42, significantly downregulated in activated LX2 cells after 3D-Exo treatment (Fig. 2c). The proliferation and chemotaxis of activated LX2 cells are particularly crucial for profibrogenic responses that determine the severity of fibrosis. Accordingly, we investigated whether 3D-Exo could halt or attenuate proliferation and chemotaxis of activated LX2 cells. Established CFSE assays showed the percentage of negative cells was significantly decreased after administration of 3D-Exo for 48 to 72 h (Fig. 2e), suggested proliferation of activated LX2 cells was profoundly reduced, which was also confirmed by CCK8 assay (Fig. 2f). Furthermore, these results were consistent with dramatic decrease of positive nucleus staining for KI67, a proliferative marker, as determined by immunofluorescence assay (Additional file 1: Fig. S4a–b).

**Fig. 4:**
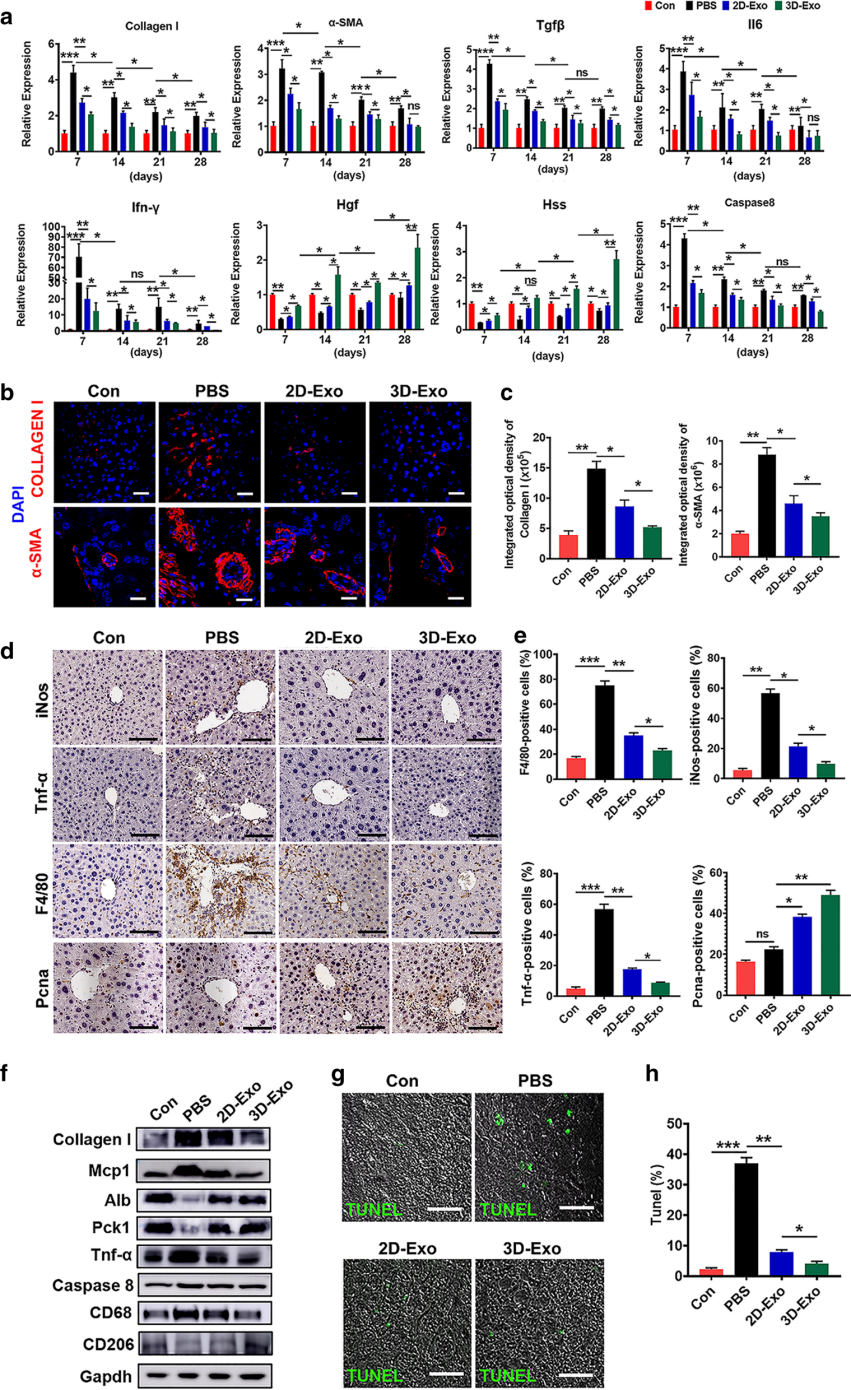
Inhibition of inflammation, apoptosis and fibrosis mediated by 3D-Exo. **a** The mRNA levels of Collagen I, α-SMA, Tgfβ, Il6, Ifn-γ, Hgf, Hss and Caspase 8 were determined by qRT-PCR in livers of mice with different treatments at 7, 14, 21 and 28 days. **b** Immunofluorescence images of Collagen I and α-SMA of livers of control mice and the fibrotic mice injected with PBS, 2D-Exo, or 3D-Exo. (Original magnification, 20×, Scale bar, 100 μm). **c** The integrated optical density in **b** was quantified. **d** Immunohistochemical staining was performed to detect the protein expressions of iNos, Tnf-α, F4/80 and Pcna in the injured liver of mice treated with 2D-Exo and 3D-Exo. (Original magnification, 20×, Scale bar, 40 μm). **e** The percentage of positive cells in **d** was calculated. **f** The protein levels of Collagen I, Mcp1, Alb, Pck1, Tnf-α, Caspase 8, CD68, CD206 were determined by western blotting in the liver tissues of the mice with different treatments. Gapdh was used as a loading control. **g** TUNEL staining of liver tissues were used to measure the apoptotic cells in mice with different treatments. (Original magnification, 20×, Scale bar, 40 μm). **h** TUNEL positive cells in **g** were quantified. n = 6 in each group. Data represent the mean ± SEM. *P < 0.05, **P < 0.01, and ***P < 0.001

Another remarkable feature of liver fibrosis is stellate cell migration toward the area of injury. Thus, we investigated the effects of hESC-Exosomes on the migration of activated LX2 cells using a cell wound healing assay. LX2 cells primed with TGFβ and exposed to 2D-Exo and 3D-Exo, showed a markedly decreased chemotaxis in both 2D-Exo and 3D-Exo groups compared to the control TGFβ-primed LX2 cells (Fig. 2g and Additional file 1: Fig. S4c). Notably, 3D-Exo showed a much better anti-chemotaxis efficacy than 2D-Exo. It has been shown that the cytosol Ca^2+^ amount is increased with the proliferation of the cells [26]. Next, we used TGFβ to activate store-operated Ca^2+^ entry into the cytosol and studied the role of 3D-Exo in regulating the cell cycle in the presence of intracellular Ca^2+^. As shown in Fig. 2h, the cytosolic Ca^2+^ level was significantly increased in proliferating LX2 activated by TGFβ, however, 3D-Exo significantly decreased the cytosolic Ca^2+^ level in activated LX2 cells, indicating slowing down the proliferation by 3D-Exo in activated LX2 cells. Then, we assessed the cell cycle distribution of activated LX2 cells after all the treatments using flow cytometry (Additional file 1: Fig. S4d), similar to the cytosolic Ca^2+^ level assay, strongly decreased population in S phase was observed in activated LX2 cells treated with 3D-Exo in comparison to the TGFβ-activated group and the activated 2D-Exo-treated group (Fig. 2i and Additional file 1: Fig. S4d). It therefore appeared that 3D-Exo had greater anti-proliferation and anti-chemotaxis abilities than 2D-Exo did. These results demonstrated 3D-Exo displayed the best anti-activation effects on stellate cells in vitro, and these in vitro effects might well indicate 3D-Exo would function as important mediators to impair the progression of liver fibrosis in vivo. Furthermore, the aforementioned results were further verified using freshly-isolated mouse primary hepatic stellate cells [27], and the results were consistent with each other (Additional file 1: Fig. S5–S8).

**Fig. 5:**
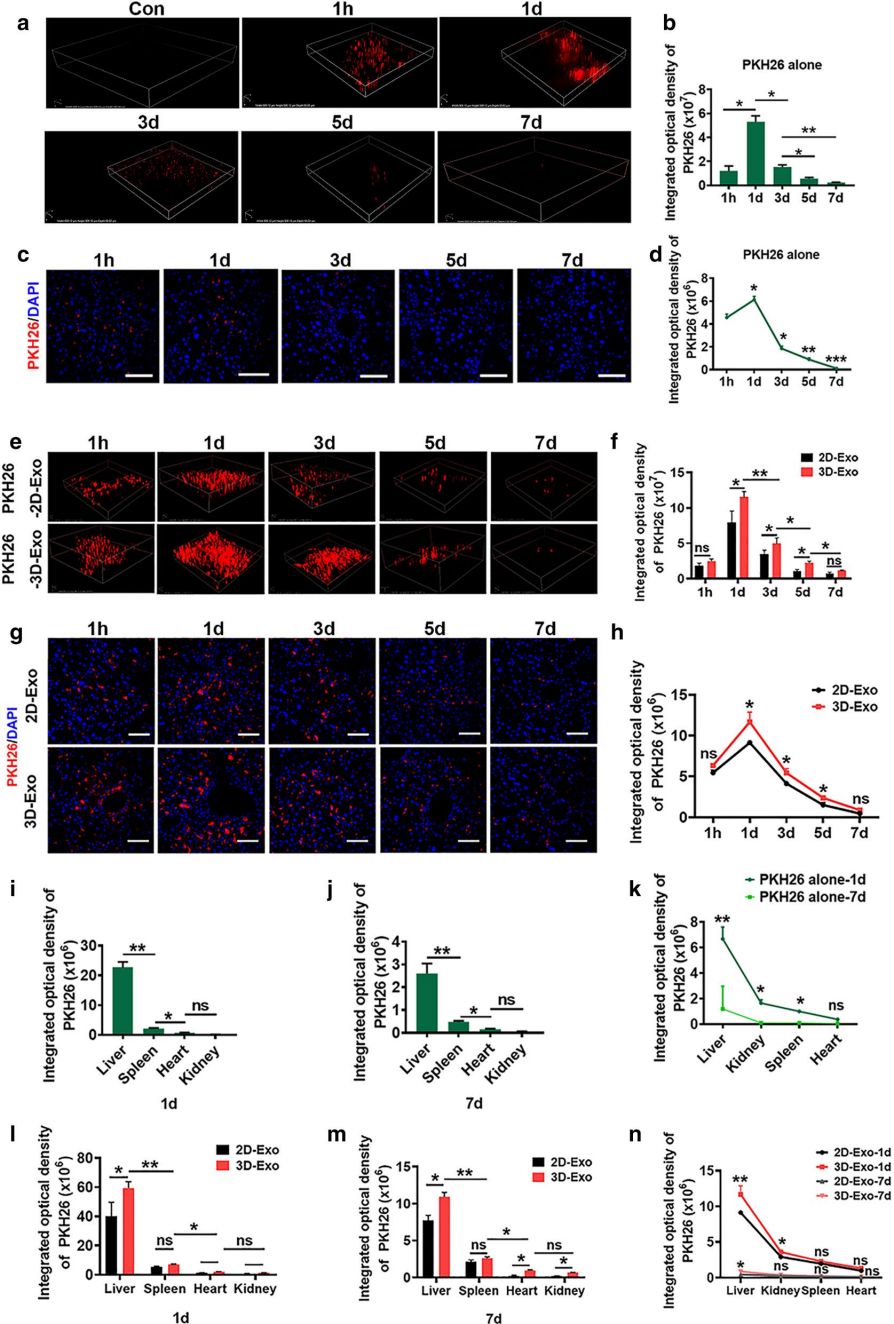
3D-Exo were significantly accumulated in livers of fibrosis mice. **a** Representative time-dependent in vivo fluorescence images of PKH26 in the livers of mice treated with PKH26 dye only. **b** Time-dependent fluorescence intensity changes of PKH26 in **a** was calculated. **c** Ex vivo CLSM images of the livers of mice sacrificed at different time points. (Original magnification, 25×, Scale bar, 40 μm). **d** Quantification of integrated optical density of PKH26 in **c**. **e** The mice were injected with pKH26-labeled 2D-Exo and 3D-Exo through tail vein. The distribution and metabolism of exosomes in livers at different time points after treatment were recorded by TPEF in vivo. **f** The integrated optical density analyses of PKH26 in **e** were quantified. **g** CLSM images and fluorescence density analysis after the injection with exosome in the livers of mice sacrificed at different time points (Original magnification, 20×, Scale bar, 40 μm). **h** The integrated optical density analyses of PKH26 in **e** were quantified. **i**, **k** Quantification of the integrated optical density of the fluorescence recorded by TPEF for the enrichment and metabolism of PKH26 dye in liver, kidney, spleen and heart at day 1 (1 d) and day 7 (7 d) respectively after caudal vein injection (TPEF images were presented in Additional file 1). **l**–**n** Quantification of the integrated optical density of the fluorescence recorded by TPEF for the enrichment and metabolism of PKH26 labeled-exosomes in liver, kidney, spleen and heart at day 1 (1 d) and day 7 (7 d) respectively after caudal vein injection (TPEF images were presented in Additional file 1). n = 6 in each group. Data represent the mean ± SEM. *P < 0.05, **P < 0.01, and ***P < 0.001

**Fig. 6:**
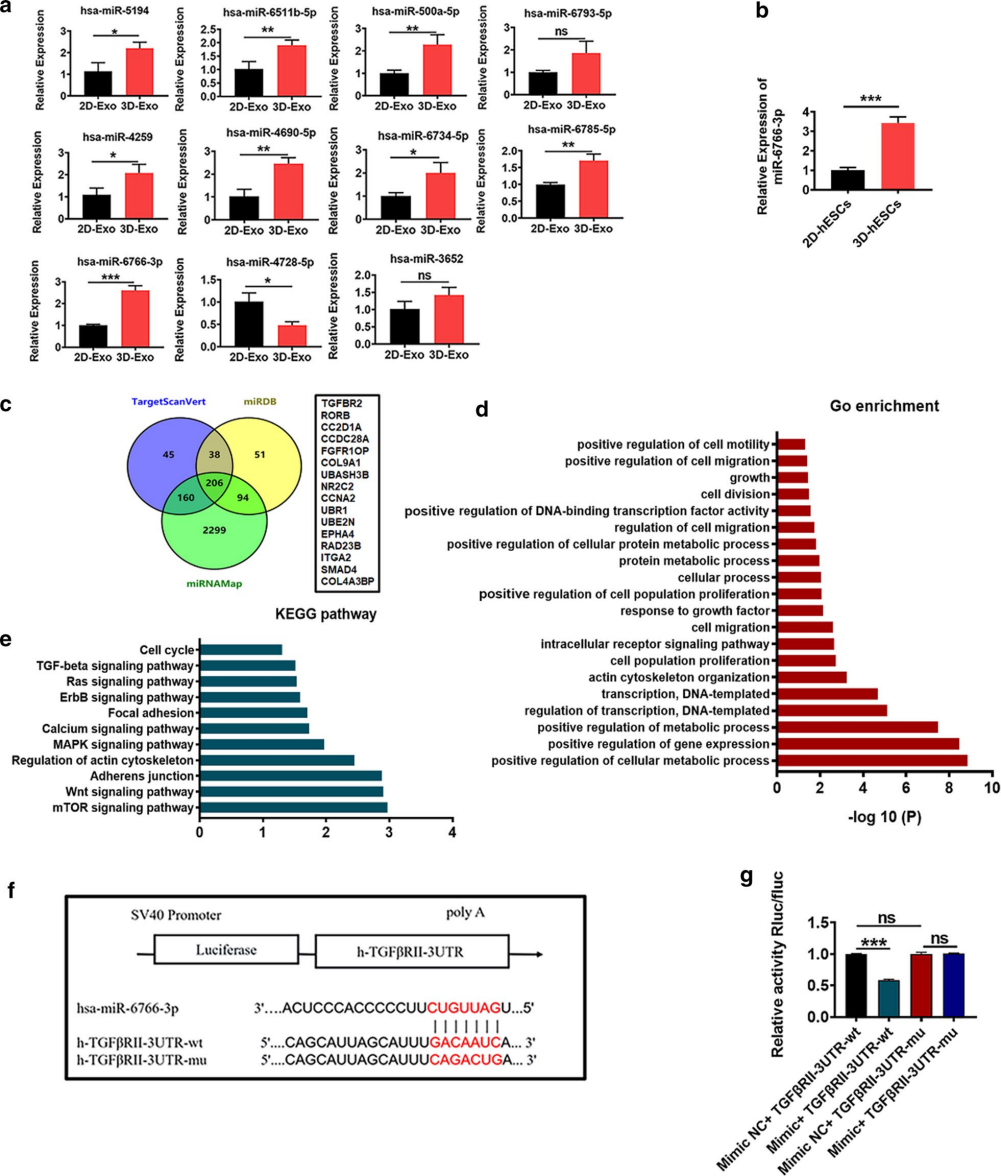
3D-Exo suppressed TGFβ-induced LX2 cell activation by delivering miR-6766-3p. **a** According to microarray analysis, 11 miRNAs were selected to verify their expressions by qPCR in 2D-Exo and 3D-Exo. **b** miR-6766-3p was highly expressed in 3D-hESCs, compared with 2D-hESCs. **c** Venn diagrams showing the intersection between predicted target genes of miR-6766-3p. **d** Go terms enriched functions for target genes of miR-6766-3p. **e** KEGG enriched pathways for target genes of miR-6766-3p. **f** A schematic drawing showing the putative binding sites of wt-TGFβRII and mut-TGFβRII in miR-6766-3p, and that the 3ʹ untranslated regions (UTRs) of TGFβRII including wild-type (WT) or mutant type (MUT) were respectively cloned downstream of the Renilla luciferase (Rluc) gene as fusion gene driven by SV40 promoter. **g** miR-6766-3p mimics or NC was co-transfected with a dual-luciferase (Rluc and the firefly luciferase (fluc)) reporter construct containing TGFβRII WT or TGFβRII MUT into HEK-293 T cells. Relative luciferase reporter activity were shown by Rluc activity versus fluc activity (Rluc/fluc). Data represent the mean ± SEM. *P < 0.05, **P < 0.01, and ***P < 0.001

**Fig. 7:**
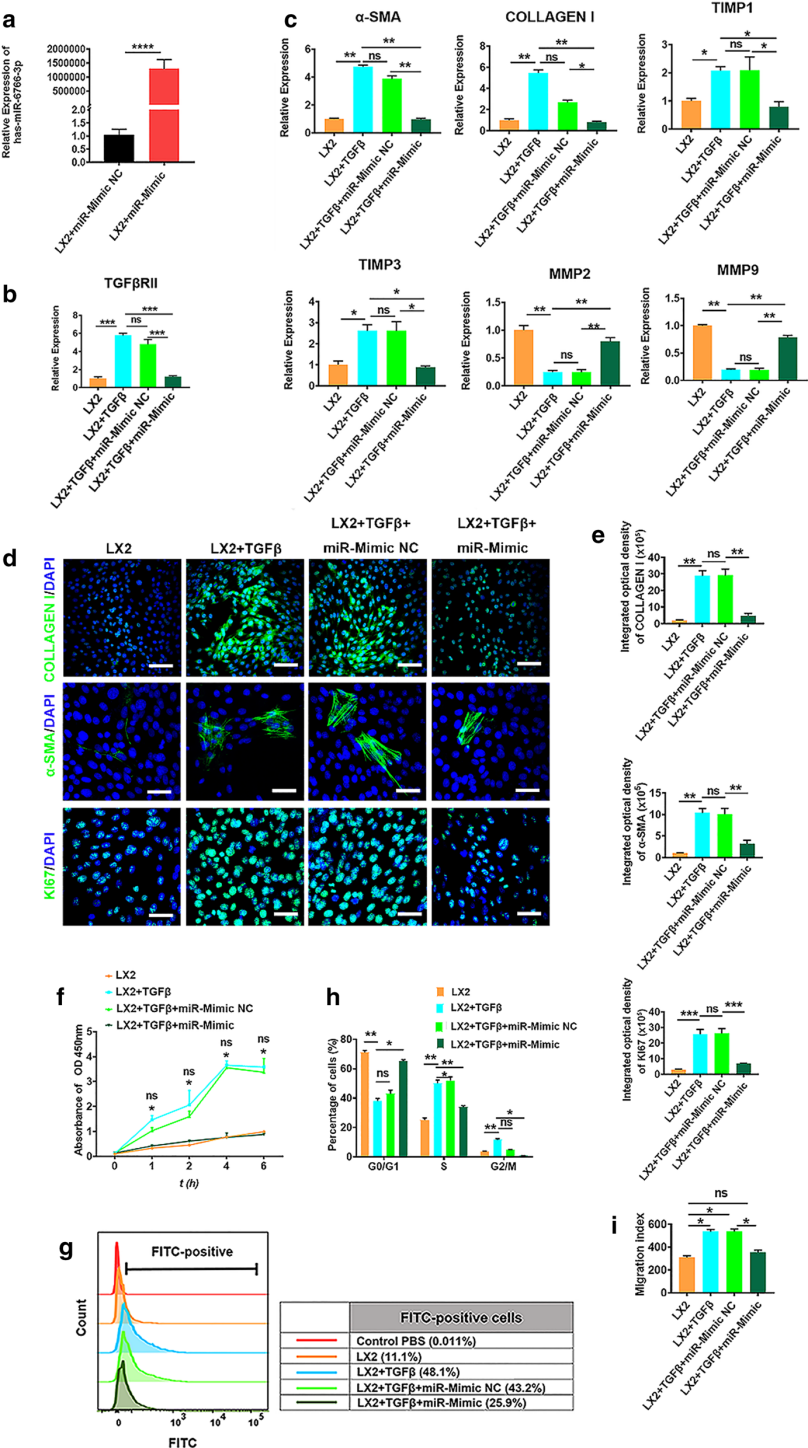
TGFβRII served as a direct target of miR-6766-3p. **a** The efficiency of transfection with miR-6766-3p mimic in LX2 cells was verified by qPCR. **b**, **c** The expression changes of TGFβRII and fibrosis-related genes (α-SMA, COLLAGEN I, TIMP1, TIMP3, MMP2, and MMP9) regulated by miR-6766-3p were verified by qPCR in LX2 cells and activated LX2 cells treated with miR-6766-3p mimic and its NC. **d** TGFβ induced profibrogenic protein expressions in activated LX2 cells, such as COLLAGEN I, α-SMA and KI67, whereas they were inhibited by miR-6766-3p after treatment with miR-6766-3p mimic or its NC. **e** Quantification of integrated optical density of genes in **d**. **f** Cell proliferation was measured using cell viability assay with CCK8 kit in LX2 cells and TGFβ-induced LX2 treated with miR-6766-3p mimic and its NC. **g** Flow cytometry assay was performed to determine the changes of calcium content in LX2 cells and activated LX2 cells after the addition of the miR-6766-3p mimic and its NC. **h** Quantification of cell cycle with the forced expression of miR-6766-3p measured by flow cytometry in LX2 cells and activated LX2 cells after treatment with the miR-6766-3p mimic and its NC. (Flow cytometry analysis used to determine the stages of cell cycle was provided in Additional file 1.). **i** Quantification of wound recovery rates modeled by cell scratch assays, in LX2 cells and activated LX2 with different treatments, and the migration index was presented over time. (The images of cell scratch assays were provided in Additional file 1.). Data represent the mean ± SEM. *P < 0.05, **P < 0.01, and ***P < 0.001

**Fig. 8:**
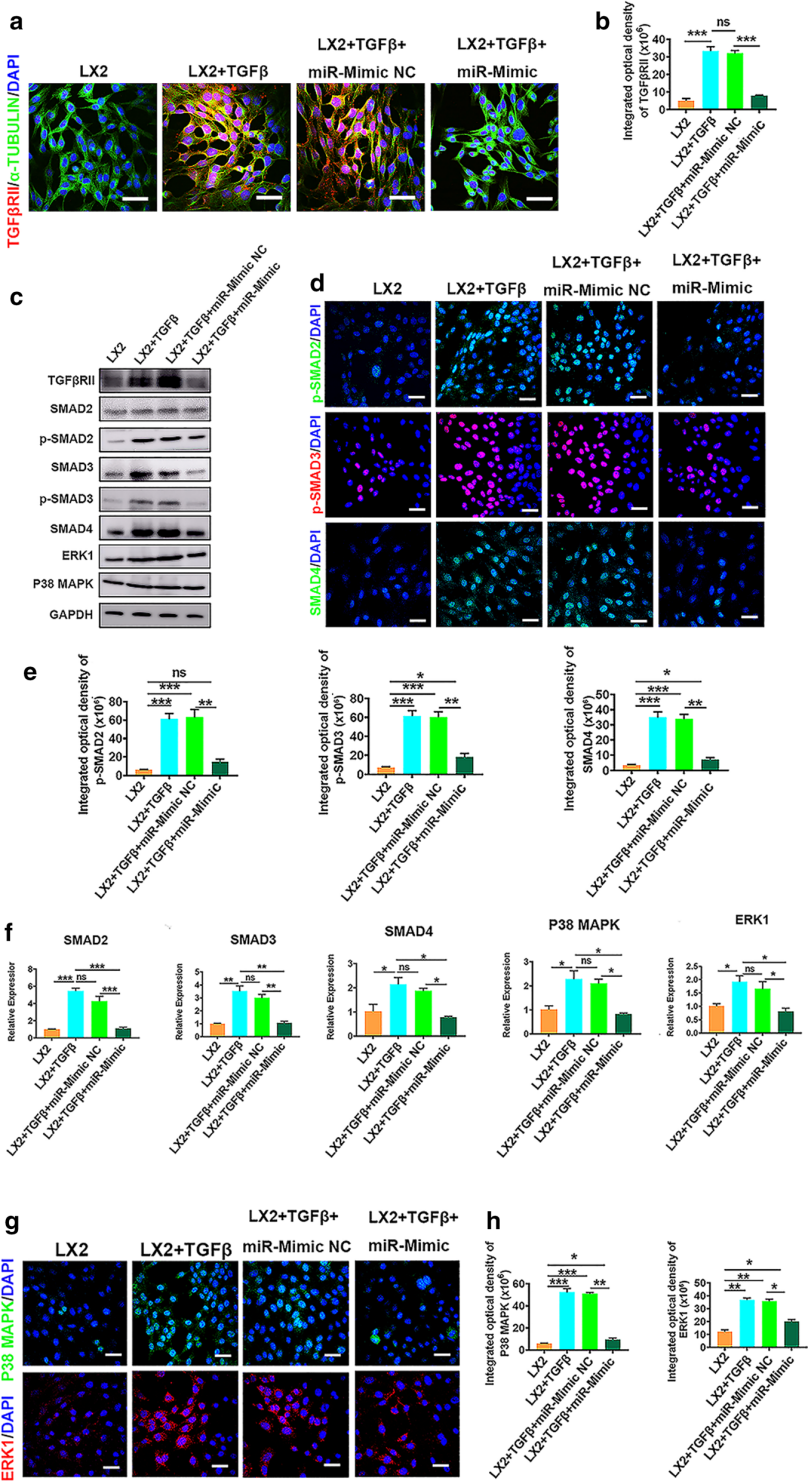
3D-Exo ameliorated fibrosis through modulating TGFβRII/Smads signaling. **a** CLSM images of TGFβRII immunostaining in TGFβ-induced LX2 cells transfected with miR-6766-3p or its NC. Red: TGFβRII. Green: Tubulin. Blue: DAPI. (Original magnification, 20×, Scale bars, 40 μm) **b** The integrated optical density analysis of TGFβRII in **a**. **c** Western blot showed expression changes of TGFβRII fibrosis pathway-associated proteins were determined by Western blot in LX2 cells and activated LX2 cells after treatment with miR-6766-3p mimic or its NC. **d** The expressions of p-SMAD2, p-SMAD3 and SMAD4 in LX2 cells and activated LX2 with different treatments were detected by immunofluorescence analysis. Red: p-SMAD2/SMAD4. Green: p-SMAD2/SMAD4. Blue: DAPI. (Original magnification, 20×, Scale bars, 40 μm). **e** The integrated optical density analysis of p-SMAD2, p-SMAD3 and SMAD4 in **d**. **f** The gene expressions of SMAD2, SMAD3, SMAD4, P38 MAPK and ERK1 were verified by qPCR in LX2 cells and TGFβ-induced LX2 cells under the treatment with miR-6766-3p mimic or its NC. **g** The expressions of P38 MAPK and ERK1 were detected by immunofluorescence staining in LX2 cells and TGFβ-induced LX2 cells after the addition of miR-6766-3p mimic or its NC. **h** Quantification of integrated optical density of P38 MAPK and ERK1 in **g**. Data represent the mean ± SEM. *P < 0.05, **P < 0.01, and ***P < 0.001

### Administration of 3D-hESC-Exosomes showed efficacious therapeutic effects in mouse model of liver injury and fibrosis

We next investigated the anti-inflammatory effects of 3D-Exo in the mouse liver fibrosis model which was induced by CCL_4_ in conjuction with employing alcohol for 8 weeks. The liver injury was shown by increasing the levels of AST, ALT, TBIL and decreasing functioning proteins like albumin (ALB), along with the deposition of collagen and lipids (Fig. 3a and Additional file 1: Fig. S9). First, we evaluated the levels of AST and ALT in mouse serum, and found that they were significantly reduced upon 3D-Exo treatment compared with PBS-treated group and 2D-Exo-treated group (Fig. 3b). Furthermore, the histopathological analysis showed that the liver of the PBS group, which was severely damaged, exhibited dilation of the sinusoidal space with inflammatory cell infiltration and necrosis (Fig. 3c, Additional file 1: Fig. S9b). Notably, the 2D-Exo- and 3D-Exo-treated groups had reduced cell necrosis and alleviated liver fibrosis, as well as more complete tissue structure (Fig. 3c). The imbalanced ECM (mainly collagen) synthesis and degradation of fibrous connective tissue caused by a large number of deposits, is the main factor for the formation of liver fibrosis, which could be detected by Masson staining (Additional file 1: Fig. S9c) [28]. As shown in Fig. 3d, a significant reduction in the fibrotic area was observed in the livers of the 2D-Exo- and 3D-Exo-treated groups compared with the PBS control group, whereas the accumulation of collagen in the livers of the 3D-Exo group was significantly lower than those in the livers of the 2D-Exo group. In addition, accumulation of the lipid usually occurs during the development of the liver fibrosis (Additional file 1: Fig. S9d) [29], the results of the oil red staining showed that the content of lipid droplets in the liver of 3D-Exo-treated mice was significantly reduced, indicating the amelioration of the liver fibrosis after treatment (Fig. 3e). Overall, the histological structure of the livers in the 3D-Exo treatment group was the closest to the normal control group (Fig. 3d–e).

**Fig. 9:**
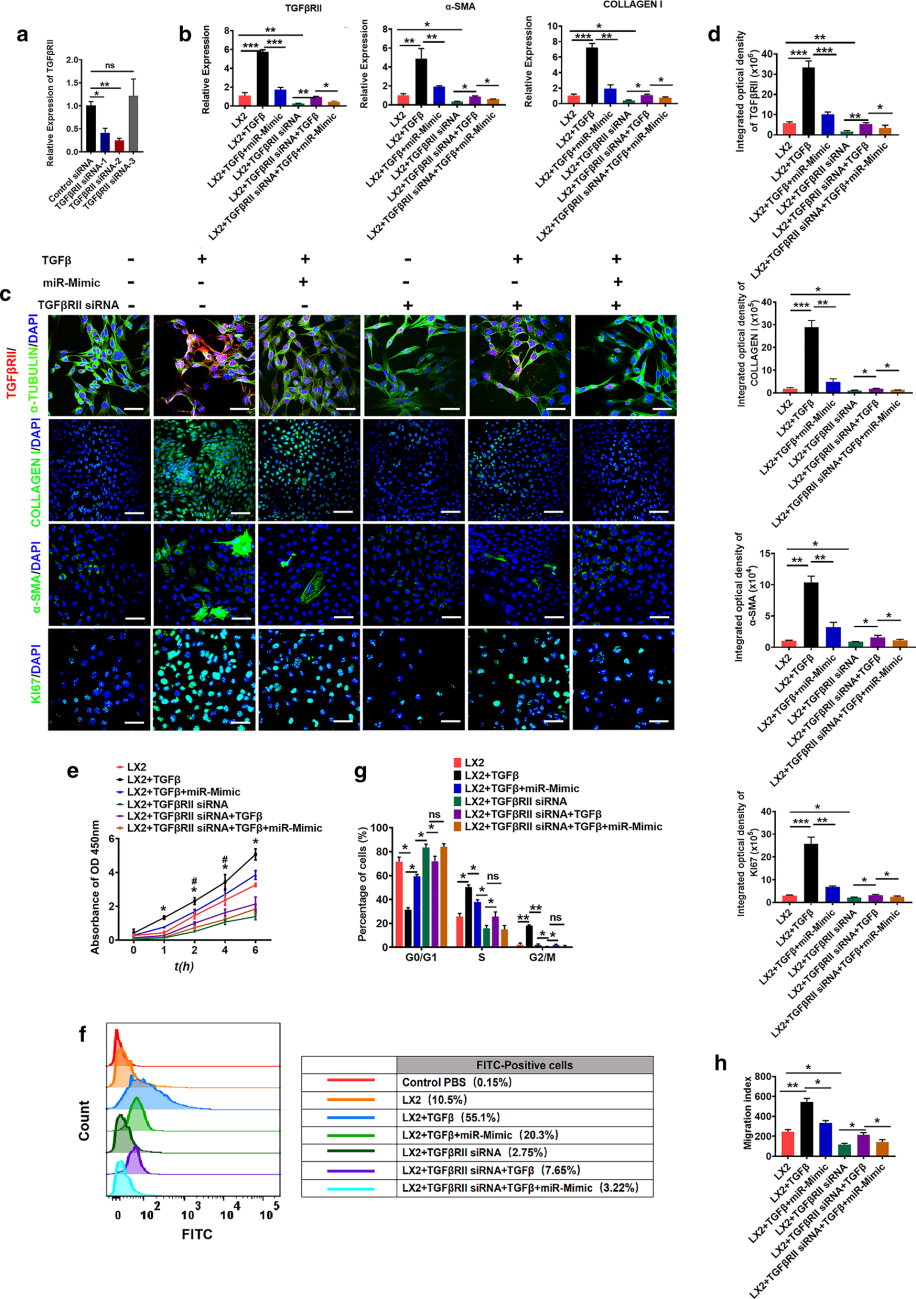
Knockdown of TGFBRII reduced LX2 cell activation. **a** Validation of siRNA knockdown efficiency of TGFBRII by qPCR. **b** Knockdown of TGFBRII inhibited the mRNA expression levels of TGFBRII, α-SMA and COLLAGEN I, determined by qPCR. **c** Knockdown of TGFBRII inhibited the protein expression levels of TGFBRII and profibrogenic protein (α-SMA, COLLAGEN I and KI67), detected by immunostaining. Red: TGFβRII. Green: Tubulin, COLLAGEN I, α-SMA and KI67. Blue: DAPI. (Original magnification, 20×, Scale bars, 40 μm). **d** Quantification of integrated optical density of proteins in **c**. **e** The capacity of cell proliferation was measured using cell viability assay with CCK8 kit in LX2 cells and TGFBRII KD LX2 cells treated with TGFβ or/and miR-6766-3p mimic. **f** Flow cytometry assay was performed to determine the changes of calcium content in LX2 cells and TGFBRII KD LX2 cells treated with TGFβ or/and miR-6766-3p mimic. **g** Quantification of cell cycle were measured by flow cytometry in LX2 cells and TGFBRII KD LX2 cells treated with TGFβ or/and miR-6766-3p mimic. **h** Quantification of wound recovery rates was modeled by cell scratch assays in LX2 cells and TGFBRII KD LX2 cells treated with different treatments, and the migration index was presented over time. (The images of cell scratch assays were provided in Additional file 1.). Data represent the mean ± SEM. *P < 0.05, **P < 0.01, and ***P < 0.001

Collagen I and α-SMA, two profibrogenic markers, were then used to monitor the progression of hepatic fibrosis in different groups of mice. We observed a significant reduction in the expressions of both Collagen I and α-SMA at both mRNA and protein levels in the livers of 3D-Exo-treated mice overtime during the treatment, as determined by qPCR (Fig. 4a), Immunofluorescence assay (Fig. 4b, c), and Western bolt (Fig. 4f, Additional file 1: Fig. S10). These data consistently showed that 3D-Exo inhibited collagen synthesis in fibrosis liver, also down-regulated the expression of α-SMA and Tgfβ (Fig. 4a), consequently alleviated the progression of liver fibrosis. Furthermore, immunohistochemistry of inducible nitric oxide synthase (iNos), tumor necrosis factor-α (Tnf-α) and EGF-like module-containing mucin-like hormone receptor-like 1(F4/80) were performed to evaluate infiltrating inflammatory cells, and the results indicated their expression were significantly decreased in the livers of 3D-Exo group compared with those of the PBS group and 2D-Exo group (Fig. 4d, e). Similar results were obtained by measuring the expression of the pro-inflammatory cytokines playing important roles in the process of fibrosis, including Interleukin-6 (Il-6), Interferon-gamma (Ifn-γ), and monocyte chemotactic protein 1 (Mcp1) overtime during the treatment of the liver fibrosis through qPCR and Western blot (Fig. 4a, f and Additional file 1: Fig. S10). These findings consistently demonstrated a more effective anti-inflammatory action of 3D-Exo in the mouse livers with injury and fibrosis.

**Fig. 10:**
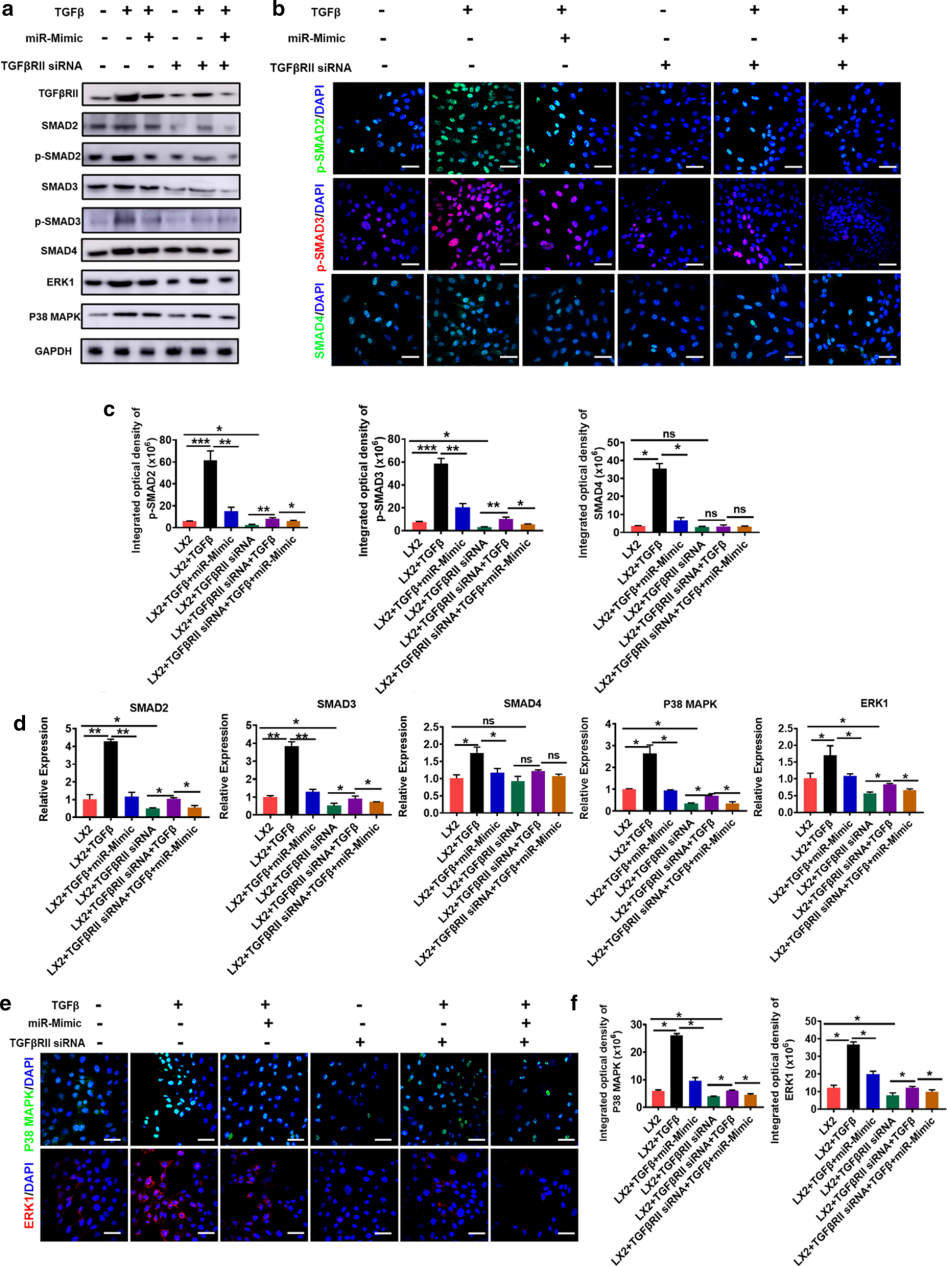
Knockdown of TGFBRII blocked the TGF-β/SMAD signaling pathway. **a** Western blot was performed to determine the expression changes of TGFβRII/SMADs pathway-associated proteins in LX2 cells and TGFBRII KD LX2 cells treated with TGFβ or/and miR-6766-3p mimic. **b** The expressions of p-SMAD2, p-SMAD3 and SMAD4 in LX2 cells and TGFBRII KD LX2 cells with different treatments were detected by immunofluorescent analysis. Green: p-SMAD2/SMAD4. Red: p-SMAD3. Blue: DAPI. (Original magnification, 20×, Scale bars, 40 μm). **c** The integrated optical density analysis of p-SMAD2, p-SMAD3 and SMAD4 in **b**. **d** The expressions of SMAD2, SMAD3, SMAD4, P38 MAPK and ERK1 were verified by qPCR in LX2 cells, TGFβ-induced LX2 cells and TGFBRII KD LX2 cells under the treatment with TGFβ or/and miR-6766-3p mimic. **e** The expressions of P38 MAPK and ERK1 in LX2 cells and TGFBRII KD LX2 cells with different treatments were detected by immunofluorescent analysis. Green: P38 MAPK. Red: ERK1. Blue: DAPI. (Original magnification, 20×, Scale bars, 40 μm). **f** The integrated optical density analysis of P38 MAPK and ERK1 in **e**

Next, to ascertain whether exosomes could promote the proliferation of hepatocytes, we conducted Proliferating Cell Nuclear Antigen (Pcna, cell nuclear antigen) immunostaining, the number of Pcna^+^ cells were observed to be markedly higher in the livers of mice injected with 3D-Exo than in those of mice injected with PBS or 2D-Exo (Fig. 4d, e). Consistent results with the increase of the markers of liver regeneration and function were obtained in qRT-PCR and Western blot analyses of hepatocyte proliferation-related genes including hepatocyte growth factor (Hgf), hepatic stimulator substance (Hss) (Fig. 4a), hepatic progenitor marker Pck1, and hepatocyte specific protein albumin (Alb) (Fig. 4f, Additional file 1: Fig. S10) in the mouse livers overtime during the treatment with 3D-Exo, these results were validated by the continuous increase of the serum albumin in the mice treated with 3D-Exo (Fig. 3b). In addition, a gene associated with liver cell apoptosis, Caspase 8 was assessed by qRT-PCR analysis. As shown in Fig. 4a, 2D-Exo and 3D-Exo administration inhibited the expression of Caspase 8 in the mouse livers overtime during the treatment when compared with those in the PBS group, particularly with the most obvious inhibitory effect in the 3D-Exo group, which was in accordance with the Western bolt results (Fig. 4f, Additional file 1: Fig. S10).

Notably, M1 microphage secreting aforementioned pro-inflammatory factors promote the inflammation and the process of the liver fibrosis, in contrast, M2 microphage function as anti-inflammation, the ratio change of M1 and M2 microphages reflects the status of liver fibrosis, increasing of M2 microphage and decreasing of M1 microphage indicate the reverse of the diseases. CD68, a M1 microphage marker, and CD206, a M2 microphage, were used to monitor the changes of these two types of microphages. Western blot revealed that CD68 was significantly decreased, whereas CD206 was contrarily increased in the mouse livers of the 3D-Exo group (Fig. 4f, Additional file 1: Fig. S10), indicating treatment with the 3D-Exo reversed the progression of the liver fibrosis. Moreover, the results of the TUNEL assay further confirmed that 3D-Exo significantly alleviated apoptosis in liver tissues of fibrosis mice after treatment (Fig. 4g, h). In particular, the expression levels of most important liver functioning protein, Alb, and Pck1, the marker of hepatic progenitor cells which are essential for liver regeneration in serious liver injury, were significantly increased in 3D-Exo treatment group (Figs. 3b, 4f, Additional file 1: Fig. S10), indicating the reconstitution of the liver and the recovery of liver function in treated fibrosis mice (Fig. 3b–e). These results might account for the phenomenon that 3D-Exo possessed superior therapeutic potential to counteract the progression of liver fibrosis than 2D-Exo. Thus, our findings strongly indicated that 3D-hESC-Exo might be a promising and effective approach for reducing or reversing liver fibrosis in patients with chronic liver disease.

### 3D-Exo possessed enhanced capacity to accumulate in livers of fibrosis mouse

Motivated by the above data indicating that 3D-Exo displayed better therapeutic effects than 2D-Exo, we evaluated whether 3D-Exo had a greater ability to migrate from vessels and penetrate into livers. The distribution of exosomes in vivo was based on PKH26-labeling characteristics of exosomes in vitro, and determined by TPEF imaging. As expected, PKH26 signals could be detected as early as 1 h, and still detectable until day 7 after intravenously injection with the dye only in the livers in all injected mice (Fig. 5a, b), moreover, free dye also could be detected even day 5 post injection by ex vivo CLSM imaging of livers dissected after sacrificing the mice at different time points (Fig. 5c, d). The dye signal in other organs was also determined, it appeared that more dyes were accumulated in the liver in vivo and ex vivo (Fig. 5i–k, Additional file 1: Fig. S11a, b). In fibrosis mice with Exos, the PKH26-Exos exhibited the capacity to accumulate in most fibrotic areas of the livers. As shown in Fig. 5e, f, TPEF imaging showed that the fluorescence intensity of PKH26 was detectable just 1 h after administration, and peaked on day 1 in the livers of all treatment mice, and obvious PKH26 signals were still detected in both Exo-treatment groups on day 7 post-injection when the PKH26 signal almost disappeared in the mice injected with the PKH26 dye alone group in vivo and ex vivo (Fig. 5a–d). We also concurrently examined fluorescence signals in different organs, and found that the strongest PKH26 signals were in the liver, followed by the kidneys, the spleen, and heart on day 1 and day 7 (Fig. 5l–n, Additional file 1: Fig: S11c, d). Subsequently, at different time points post-administration, mice were sacrificed, and different organs were prepared and imaged using CLSM for immunofluorescence analysis. Consistent results were shown that immunofluorescence of PKH26-labeled-Exos was detected just 1 h, and still available until on day 7 after administration in the livers in all injected mice (Fig. 5g, h). Furthermore, more PKH26-labeled Exos were accumulated in the livers than the other organs from day 1 to day 7 (Additional file 1: Fig. S11e, f). Collectively, these results indicated that exosomes derived from hESCs specifically targeted the liver injury sites. In particular, of all treatments, the PKH26-3D-Exo group had the most targeted accumulation in most fibrotic areas of the livers, thus, it appeared that 3D-Exo had greater capacity to be taken up by the livers of these mice than 2D-Exo did.

**Fig. 11:**
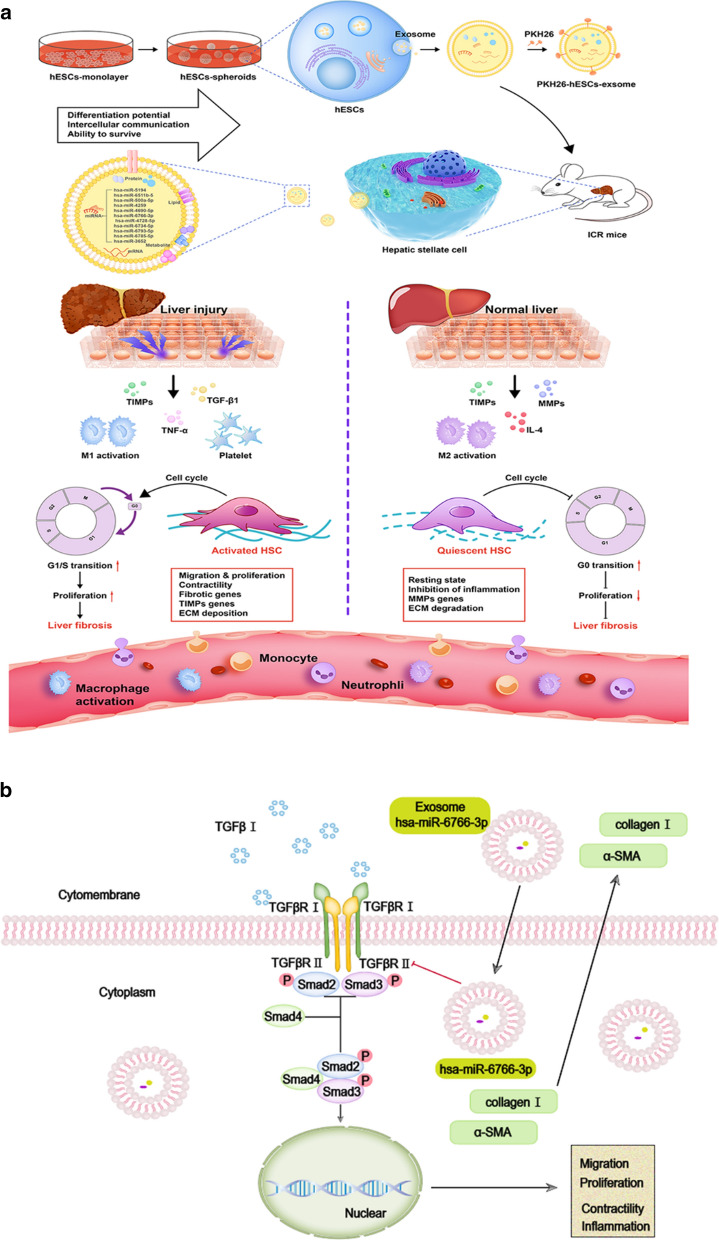
Schematic diagram of exosomes from 3D-hESCs with suppression for fibrosis through modulating TGFβRII signaling-related small RNA expression. **a** During the development of liver fibrosis, the stellate cells was activated, acquired the capacity of the proliferation and became myofibroblasts, which produced and released excessive extracellular matrix (ECM) causing the pathological change of the livers; meanwhile, M1 macrophage was activated, and secreted a large number of pro-inflammatory factors and promoted the inflammation, leading to the progression of the liver fibrosis. The delivery of hESC-derived exosomes inhibited the proliferation of activated stellate cells, decreased the deposition of ECM, reduced the expression of pro-inflammatory factors; and M2 macrophage were increased, and played anti-inflammatory roles, reversing the liver fibrosis. Thus, hESC-derived exosomes exhibited anti-fibrotic effect by alleviating activation of stellate cells and suppressing the progression of liver fibrosis. **b** The internalized miR-6766-3p enriched from hESC-Exosomes bond the 3ʹUTR of TGFβRII and inhibited the expression of TGFβRII, consequently repressing the expression of nuclear phosphorylated p-SMAD2, p-SMAD3 and SMAD4, the downstream genes of TGFβRII. These decreased SMADs pathway-associated proteins failed to form homologous oligomers, and could not enter the nucleus of the target cells to regulate the transcriptional activities including proliferation, migration, and inflammation, suppressing the progression of liver fibrosis. Thus, the molecular mechanism by which miR-6766-3p enriched from hESC-derived exosome ameliorated liver fibrosis was to attenuate activated stellate cells through targeting TGFβRII-SMAD2/3 pathway

### miR-6766-3p acted as a novel upstream regulator for TGFβRII by directly targeting its 3′UTR

To screen and identify the candidate miRNAs that enhanced the antifibrotic effect in CCl_4_-induced fibrosis, a miRNA microarray was used to profile the simultaneously upregulated or downregulated miRNAs between 2D-Exo and 3D-Exo (Additional file 1: Fig. S12a). Microarray data have been deposited in the NCBI Gene Expression Omnibus and are accessible through the GEO Series accession number GSE164617. Microarray data analysis showed that 147 miRNAs were upregulated, 173 miRNAs were downregulated and 711 miRNA were not differentially expressed between 2D-Exo and 3D-Exo (Additional file 1: Fig. S12b). Intriguingly, 39 miRNAs were upregulated and 29 miRNAs were downregulated (> 1.5-fold) in 3D-Exo compared with those in 2D-Exo, whereas no change were detectable in the expression of 953 miRNAs (Additional file 1: Fig. S12c). The top eleven of the altered miRNAs were selected and validated by qRT-PCR (Fig. 6a). Among these miRNAs, miR-6766-3p was most significantly up-regulated, which was consistent with its high expression in 3D hESCs (Fig. 6a, b). We subsequently employed TargetScanVert, miRanda and miRNAMap software to identify potential target genes of miR-6766-3p, and Venn diagrams showed the intersection among the predicated targeted genes of miR-6766-3p (Fig. 6c). Association analysis was applied to screen functions and pathways enriched for differentially expressed genes. The enrichment functions had cell division, cell population proliferation, cell migration and other cellualr process regulation-associated activities (Fig. 6d), while the enrichment pathways included TGF-β signaling pathway, calcium signaling pathway, MAPK signaling pathway, mTOR signaling pathway and other pathways (Fig. 6e).

TGF-β signaling pathway plays a critical role in liver fibrosis [30]. Meanwhile, our bioinformatic analysis indicated that the 3′UTR of the TGFβ type II receptor (TGFβRII), which acts as the receptor for TGFβ and participates in orchestrating fibrogenesis [31], comprises a predicted binding site for miR-6766-3p (Fig. 6f). Then, the dual-luciferase reporter system in which the protein expression of the Renilla luciferase (Rluc) rather than the firefly luciferase (fluc) depends on the binding between miR-6766-3p and its target site in transcribed mRNA of fused Rluc with wild-type (wt) TGFβRII 3′UTR or with mutant-type (mut) TGFβRII 3′UTR, was employed to further confirm whether miR-6766-3p directly targets TGFβRII by binding TGFβRII 3′UTR. miR-6766-3p mimics was found to remarkably reduce the level of the Rluc activity shown by relative activity Rluc/fluc, in the recipient cells transfected with the vector containing fused Rluc with wt TGFβRII 3′UTR, indicating that the translation of the Rluc was suppressed by the binding of miR-6766-3p to its target in mRNA of this fused gene. But miR-6766-3p mimics NC did not affect the level of the Rluc activity in the recipient cells transfected with one with fused the Rluc with mut TGFβRII 3′UTR, suggesting no binding between miR-6766-3p mimic NC and mut TGFβRII 3′UTR (Fig. 6g), these results further confirmed TGFβRII 3′UTR as targetting site of miR-6766-3p. Overall, these data demonstared that miR-6766-3p might exert anti-fibrogenic effects by negatively regulating TGFβRII expression through directly binding to its 3′UTR sequences.

### miR-6766-3p reduced cell proliferation, chemotaxis and expression of profibrotic markers in activated LX2 cells

To investigate the functional role of miR-6676-3p in liver fibrosis, we performed gain-of-function and loss-of-function experiments in LX2 cells which was activated in the presence of TGFβ. First, LX2 cells were transfected with miR-6676-3p mimics and its negative control (NC), and miR-6676-3p expression was confirmed by qRT-PCR (Fig. 7a). Next LX2 cells were activated by TGFβ to induce the proliferation of LX2 cells, consequently increasing the expression of TGFβRII (Fig. 7b) and other genes; afterwards activated LX2 cells were transfected with miR-6676-3p mimics, mimic negative control (Mimic NC), or miR-6676-3p inhibitors, inhibitor negative control (Inhibitor NC). The results showed that TGFβRII expression was profoundly decreased after miR-6676-3p restoration (Fig. 7b), whereas this reduction could be rescued by miR-6676-3p inhibitors (Additional file 1: Fig. S13a). We further demonstrated that miR-6676-3p downregulated the mRNA expression of α-SMA, COLLAGEN I, TIMP1, TIMP3 and upregulated MMP2, MMP9 (Fig. 7c), which are all profibrogenic genes in which TIMP1/3 antagonize MMP2/9 which decompose the collagen I during the progression of liver fibrosis. However, these gene expressions could be reversed by miR-6676-3p inhibitors (Additional file 1: Fig. S13b), indicating that miR-6676-3p negatively modulated these profibrogenic genes in TGFβ-activated LX2 cells. In agreement with this observation, immunofluorescence analysis also showed low percentages of positive cells for α-SMA and COLLAGEN I (Fig. 7d, e), indicating the protein levels of these two profibrogenic markers were reduced by miR-6676-3p mimics; similarly, this reduction could be inhibited by miR-6676-3p inhibitors (Additional file 1: Fig. S13c, d). Taken together, these results revealed that TGFβRII expression and its downstream profibrogenic markers of TGFβ were mediated by miR-6676-3p.

We next sought to verify whether miR-6766-3p might exert inhibitory effects on proliferation and chemotaxis of activated LX2 cells. All groups of cells were treated with TGFβ in advance in order to trigger the profibrogenic phenotypes to activate LX2 cells. As a result, miR-6766-3p mimics markedly decreased the growth rate of TGFβ-treated LX2 cells, measured by CCK8 assay (Fig. 7f), but this decrease could be prevented by miR-6676-3p inhibitors (Additional file 1: Fig. S14a). Notably a lower positive cell percentage for KI67 which is an indicator of cell proliperation, was observed in miR-6766-3p-transfected LX2 cells than in miR-6766-3p mimic negative control-treated cells (Fig. 7d, e), but the percentage number of these positive cells could be increased by miR-6676-3p inhibitors (Additional file 1: Fig. S14b). Additionally, intracellular calcium concentration assays showed that miR-6766-3p mimic substantially decreased the cytosolic Ca^2+^ level in activated LX2 cells (Fig. 7g). In contrast, miR-6766-3p knockdown by its inhibitors in activated LX2 cells dramatically enhanced the Ca^2+^ level (Additional file 1: Fig. S14c).

Based on the evidence that miR-6766-3p significantly inhibited the activated LX2 cell proliferation, we speculated that miR-6766-3p might display its anti-proliferative function by affecting the cell cycle of the activated LX2 cells. By means of cell cycle assay using flow cytometry (Additional file 1: Fig. S15a), we found that the accumulation of the activated LX2 cells in S and G2/M-phase (35%) was notably reduced, along with a significant increase of cells in G1-phase (65%) after treatment with miR-6766-3p mimics, in comparison with TGFβ-treated LX2 cells (G2/M-phase, 61%, and G1-phase, 38%) (Fig. 7h). Furthermore, miR-6766-3p mimics NC did not induce alteration of cell number in the four distinct phases of cell cycle compared with TGFβ-treated cells, suggesting a critical role of miR-6766-3p in modulating the cell cycle of the activated LX2 cells. As expected, miR-6766-3p knockdown by its inhibitors dramatically increased the number of cells in the S peak and decreased those in the G1 peak (Additional file 1: Fig. S15b, c). Hence, we summarized that miR-6766-3p was able to limit proliferation and intracellular calcium concentration in the activated LX2 cells by blocking the G1/S cell cycle transition. Next, the results of cell wound healing assay presented in Fig. 7i and Additional file 1: Fig. S15d indicated that miR-6766-3p mimics significantly halted migration of the activated LX2 cells when compared to TGFβ-treated LX2 cells and miR-6766-3p mimic NC-treated cells, however this phenomenon could be reversed by the miR-6766-3p inhibitor (Additional file 1: Fig. S15e, f). The above results strongly demonstrated that miR-6766-3p regulated not only the expression of profibrogenic markers in the activated LX2 cells, but also secondary cell responses, such as proliferation and migration, ultimately modulating the progression of liver fibrosis. Together, these data further confirmed function enrichments on cell cycle, cell population proliferation, cell division, calcium signaling pathway, cell migration in the prediction and enrichment analysis of target genes of miR-6766-3p (Fig. 6d, e).

### miR-6766-3p repressed LX2 cell activation through TGFβRII/SMADS pathway

To further clarify the mechanism underlying the regulation of liver fibrosis by the miR-6766-3p/TGFβRII axis, TGFβ treated-LX2 cells were transfected with miR-6766-3p mimics, miR-6766-3p inhibitor, and their negative controls to measure the expression levels of TGFβRII. Immunofluorescence staining showed that the number of the TGFβRII positive cells was extremely low in miR-6766-3p mimic-treated LX2 cells (Fig. 8a, b), indicating that the expression of TGFβRII was significantly suppressed. By contrast, the percentage of TGFβRII positive cells in the activated LX2 cells treated with a miR-6766-3p inhibitor was higher than its controls (Additional file 1: Fig. S16a, b). Similar results were acquired by Western blot analysis at the protein levels of TGFβRII in the activated LX2 cells with the treatments of miR-6766-3p mimics and inhibitors (Fig. 8c and Additional file 1: Fig. S16c–e).

SMADs, crucial downstream signal transduction molecules of TGFβ1, have been identified as important mediators of fibrogenesis [32]. Thus, we determined whether miR-6766-3p could also alter the activation and phosphorylation of SMADs. Western blot analysis revealed phosphorylation levels of SMAD2 and SMAD3 were dramatically reduced in the activated LX2 cells receiving miR-6766-3p mimics (Fig. 8c, Additional file 1: Fig. S16c). In addition, these decreased phosphorylation levels elicited by miR-6766-3p mimics was completely blocked by treatment with miR-6766-3p inhibitors (Additional file 1: Fig. S16d, e). Immunofluorescence assays showed that the percentages of nuclear positive staining for phosphorylated p-SMAD2/3 and SMAD4 was dramatically decreased in activated LX2 cells (Fig. 8d, e), further confirming miR-6766-3p mimics repressed expression of nuclear phosphorylated p-SMAD2, p-SMAD3 and SMAD4. Moreover, the addition of the miR-6766-3p inhibitor reversed the effects of miR-6766-3p mimics, strongly enhancing the expressions of p-SMAD2, p-SMAD3 and SMAD4 in the activated LX2 cells, as shown by increasing the positive staining of these three downstream genes in immunofluorescence assays (Additional file 1: Fig. S17a, b).

The ability of miR-6766-3p to impair activation and phosphorylation of SMADs prompted us to evaluate other factors involved in the TGFβRII /SMADs signaling pathway, such as ERK1 and P38 MAPK, two upstream modulators of SMADs [23, 33]. To verify our speculation, we measured the expressions of P38 MAPK and ERK1 under the circumstance that the expressions of the downstream genes SMAD2 and SMAD3 as well as SMAD4 were suppressed by miR-6766-3p. As expected, qPCR results revealed that two upstream modulators of SMADs, P38 MAPK and ERK1, and the downstream genes SMADs were consistently and significantly downregulated in the activated LX2 cells treated by miR-6766-3p mimics (Fig. 8f); in contrary, all five genes mentioned above were concurrently upregulated by the miR-6766-3p inhibitors in the activated LX2 cells (Additional file 1: Fig. S18). Immunofluorescence assay was used to further confirm the expression changes of these two upstream modulators by miR-6766-3p. Similarly, the percentages of positive cells for P38 MAPK and ERK1 were significantly lower in the activated LX2 cells treated with miR-6766-3p mimic than those in its NC control-treated cells and the activated LX2 cells (Fig. 8g, h), indicating that the expressions of ERK1 and P38 MAPK were reduced by miR-6766-3p; moreover, this reduction was inhibited in the activated LX2 cells treated with miR-6766-3p inhibitors by increasing the positive cells for ERK1 and P38 MAPK (Additional file 1: Fig. S19a, b). These results also further confirmed pathway enrichments on TGFβ signaling pathway, MAPK signaling pathway, SMADs pathways in the prediction and enrichment analysis of target genes of miR-6766-3p (Fig. 6c, e).

### Knockout (KD) of TGFBRII reduced cell proliferation, chemotaxis and expression of profibrotic markers in LX2 cells

To characterize the effects of TGFBRII on liver fibrosis and further confirm whether miR-6766-3p specifically targets TGFβRII, we knocked down the expression of TGFBRII in LX2 cells employing siRNAs which specifically target mRNA of TGFBRII. The mRNA levels of TGFBRII were significantly decreased after TGFβRII siRNA-2 transfection (Fig. 9a). Next TGFβRII KD LX2 cells were treated with TGFβ or/and miR-6676-3p mimics, the results showed that TGFβRII expression was profoundly decreased in TGFβRII KD LX2 cells, and slightly increased after TGFβ stimulation, however this increase was inhibited again with the treatment of miR-6676-3p mimics (Fig. 9b). We further demonstrated that the mRNA expressions of α-SMA, COLLAGEN I were consistent with the decrease of TGFβRII (Fig. 9b). Consistently with this observation, immunofluorescent analysis also showed lower percentages of positive cells for α-SMA and COLLAGEN I in TGFβRII KD LX2 cells (Fig. 9c, d); similarly, miR-6676-3p mimics could also significantly inhibit these up-regulation induced by TGFβ in TGFβRII KD LX2 cells (Fig. 9c, d). Thus, these results indicated that the decreased expression of these downstream profibrogenic markers of TGFβ were mediated by the knockout of TGFβRII. We next sought to verify whether TGFβRII KD might exert inhibitory effects on proliferation and chemotaxis of LX2 cells. As expected, the growth rate of LX2 cells was markedly decreased by TGFβRII KD, measured by CCK8 assay (Fig. 9e), but slightly increased after TGFβ stimulation, this phenomenon could be reversed by the miR-6766-3p (Fig. 9e). Notably a number of KI67 positive cells was observed in TGFβRII KD LX2 cells, then the number of these positive cells was decreased by the treatment with miR-6676-3p mimics in TGFβ activated TGFβRII KD LX2 cells (Fig. 9c). Additionally, intracellular calcium concentration assays showed a decrease of calcium content after TGFβRII KD in LX2 cells. Consistently, miR-6766-3p mimic substantially decreased the cytosolic Ca^2+^ level in TGFβ activated TGFβRII KD LX2 cells. (Fig. 9f). As expected, TGFβRII KD dramatically decreased the number of cells in the S phase, then blocked the cell cycle and inhibited proliferation of LX2 cells (Fig. 9g and Additional file 1: Fig. S20a). Next, the results of cell wound healing assay presented in Fig. 9h and Additional file 1: Fig. S20b indicated a significantly halted migration in TGFβRII KD LX2 cells. Taken together, these results strongly demonstrated that TGFβRII KD down-regulated not only the expression of profibrogenic markers, but also secondary cell responses, such as proliferation and migration, ultimately modulating the progression of liver fibrosis.

### Knockdown of TGFBRII reduced LX2 cell activation by inhibiting the TGF-β/SMAD pathway

To further verify whether miR-6766-3p acts solely through TGFβRII, TGFβRII was knocked down in LX2 cells, TGFβ or/and miR-6676-3p mimics was further used to treat TGFβRII KD LX2 cells to measure the expression levels of SMADs. Western blot analysis revealed that the phosphorylation levels of SMAD2 and SMAD3 were dramatically reduced in TGFβRII KD LX2 cells, but slightly increased after TGFβ stimulation, similarly this phenomenon could be suppressed by the treatment with miR-6766-3p mimics (Fig. 10a, Additional file 1: Fig. S21). Immunofluorescent assays showed that the percentages of phosphorylated p-SMAD2/3 positive cells was dramatically decreased in TGFβRII KD LX2 cells (Fig. 10b, c), consistently, miR-6676-3p mimics could also substantially repressed the up-regulation of nuclear phosphorylated p-SMAD2 and p-SMAD3 induced by TGFβ in TGFβRII KD LX2 cells (Fig. 10b, c). Furthermore, we measured the expressions of P38 MAPK and ERK1 under the circumstance that the expressions of the downstream p-SMAD2/3 were suppressed by TGFβRII KD. As expected, qPCR results revealed that P38 MAPK and ERK1, as well as the downstream genes SMAD2/3 were significantly downregulated in TGFβRII KD LX2 cells (Fig. 10d); Similarly, the percentages of positive cells for P38 MAPK and ERK1 were significantly lower in TGFβRII KD LX2 cells, and also be inhibited by miR-6766-3p after TGFβ stimulation in TGFβRII KD LX2 cells (Fig. 10e, f). Importantly, among all five genes mentioned above, the expression of SMAD4, a potential target of miR-6766-3p in Fig. 6c, was not significantly inhibited by miR-6766-3p after TGFβ stimulation in TGFβRII KD LX2 cells (Fig. 10d), whereas SMAD4 was dramatically down-regulated by miR-6766-3p in TGFβ-activated LX2 cells (Fig. 8c–f), these results indicated that miR-6766-3p targeted SMAD4 through TGFβRII rather than direct targeting. In conclusion, these aforementioned data strongly demonstrated that miR-6766-3p played a vital role in alleviating LX2 cell activation and suppressing the progression of liver fibrosis through targeting the TGFβRII/SMADS pathway (Fig. 11a, b).

## Discussion

hPSCs, including both hESCs and hiPSCs, have been proposed for regenerative medicine and tissue replacement after injury or disease, owing to their plasticity and potentially unlimited capacity of self-renewal [12, 13, 20]. For clinical applications, MSCs are expanded by continuously passages in vitro in order to obtain a sufficient number of cells. However, MSCs exhibit passaging-induced senescence, such as increased DNA damage foci, loss of telomerase and loss of stemness ability, ultimately leading to reduced therapeutic effects [11]. Unlike MSCs, hESCs derived from blastocyst-stage embryos, have been proven to possess great plasticity and unlimited self-renewal ability [34]. It is interesting to note that hESC, due to their anti-senescence ability, have been shown to significantly reverse the aging MSCs and improve the therapeutic effect of MSCs [35]. This provides a better alternative for regenerative medicine and tissue replacement after injury or disease. However, the major barriers to the possible transplantation of hESCs into patients are ethical issues and tumorigenic potential (Additional file 1: Fig. S2). Recent studies have suggested that extracellular vesicles which are biological particles released by various cell types, transfer proteins and nucleic acids between cells, and exhibit anti-apoptotic, pro-angiogenic, and anti-fibrotic properties [19]. In fact, a broad interest pointed to exosomes secreted from embryonic stem cells, which are capable of instigating cell analogous response in target cells. Unlike hESCs, no teratomas were formed after injection of hESC-derived exosomes in immunodeficient mice (Additional file 1: Fig. S2a–c). Collectively, these results suggested that hESC-derived exosomes could serve as a promising and effective cell-free strategy for regenerative medicine.

LX2 cells, a stellate cell line derived from normal liver, are retinoic aldehyde storing cells, and once activated, they proliferate and become myofibroblasts, which produce and release excessive extracellular matrix, during liver injury [36]. Therefore, the blockade or inactivation of LX2 cells is an extremely important intervention for determining anti-fibrosis treatment. It had been verified in our present study that exosomes were capable to be internalized and integrated into target cells and regulate cellular phenotypes. Furthermore, it has been demonstrated that 3D spheroids of MSCs and human dermal fibroblasts possess greater therapeutic potential than their 2D monolayer cultured cells [37, 38]. Accordingly, we speculated that exosomes derived from 3D-hESCs might show better efficacy on the treatment of liver fibrosis than 2D-hESC-Exosomes. To this end, we compared the effects of exosomes derived from 2D-hESCs with those of 3D-hESC-Exosomes on the proliferation, migration, and function of TGFβ-activated LX2 cells in vitro. Our results revealed that LX2 cells took up exosomes immediately after being exposed to hESC-Exosomes. Further analyses also indicated a significant reduction of the key profibrogenic genes and proteins, such as α-SMA, Collagen I and TIMP1 in TGFβ activated LX2 cells treated with 3D-Exo compared to the 2D-Exo treated group. Besides, compared to the TGFβ-treated cells, all cells treated with hESC-Exosomes showed a significant decrease in wound recovery and cell proliferation. Notably, among all the treatments, 3D-Exo resulted in the lowest concentration of intracellular calcium, and the lowest proportion of cells in S phase of cell cycle in activated LX2 cells. It was inferred from these results that 3D-Exo had greater anti-proliferation and anti-chemotaxis abilities than 2D-Exo, to some extent, our observation illustrated that 3D-Exo displayed the best anti-activation effect on stellate cells, leading to significantly reduced crucial responses of stellate cells during liver fibrosis. It is important to note that the present evidence relies on the interesting in vitro data, convincing data also needed to be confirmed in the mouse model of liver fibrosis. Consequently, to clarify the effects of 2D- and 3D-Exo on regulating liver fibrosis in vivo, we first evaluated the accumulation of hESC-Exosomes in the livers of fibrosis mice after the administration through intravenous injection. Just 1 h after injection of PKH26 labeled-hESC-Exosomes, most of the fluorescent signal was localized in the livers and low level of signal was detectable in other organs. While compared to the PKH26-3D-Exo, PKH26-2D-Exo showed a remarkably less accumulation at the same time in the liver. This strongly suggests that an enhancing antifibrotic efficacy is maximized by exosomes that contain different bioactive molecules (miRNAs, proteins, and lipids) accumulating at the intended site [39, 40]. This has been found in other systems. For example, 3D-tumour-cell-derived microparticles (3D MPs) have been shown to be softer and more deformable than 2D-tumour-cell-derived microparticles (2D MPs). This enables greater penetration of 3D MPs into target tissue for achieving effective internalization into target cells, ultimately enhancing their ability to deliver drugs [41]. The basis for this phenomenon was yet unknown.

In this liver fibrosis model, we found that administration of hESC-Exosomes resulted in obvious enhancement of fibrinolytic substance activity and reduction of key profibrogenic ECM accumulation, thereby inhibiting liver fibrosis, reconstituting the liver and restoring liver function in treated fibrosis mice. Importantly, 3D-Exo displayed the most significant antifibrotic effects among all the treated groups, further strengthening the in vitro data. Quantification of Masson staining demonstrated that collagen deposition was significantly reduced in the livers of fibrosis mice injected with 3D-Exo. Molecular analysis also showed a most significant downregulation of key profibrogenic genes α-SMA, Collagen I, and TIMP3 in fibrosis mice injected with 3D-Exo. This might be due to the fact that the three-dimensional cell culture system better maintain primitive functions and characteristics of stem cells, more accurately mimicked the natural physiological environment of tissues, released higher levels of cytokines, expressed increased pluripotent markers and displayed prolonged in vivo retention. From this standpoint, our study indicated that the 3D-Exo might be a promising and effective approach in reducing or reversing liver fibrosis in patients with chronic liver disease.

The possible mechanisms of liver repair induced by exosomes remain unclear. Emerging evidence suggests that exosomes communicate with target cells through delivering specific miRNAs from their parental cell sources. Therefore, we speculated that hESC-specific miRNAs carried by exosomes are involved in the regulation of fibrotic signal pathway activation. To characterize miRNA information of hESC-Exosomes, we performed miRNA microarray and identified a key miRNA miR-6766-3p in abundant miRNAs. In miRNA profiles of GSE69787 from the Gene Expression Omnibus database, miR-6766-3p has been shown to be a key miRNA in regulating LEF1 expression, thus participating in WNT signaling pathway [42]. It remains unknown whether miR-6766-3p plays a role in the process of liver fibrosis. We next explored our speculation whether miR-6766-3p could reduce or reverse liver fibrosis by modulating LX2 cell activation. As expected, the expressions of profibrogenic markers (α-SMA, Collagen I, and TIMP3) were significantly depressed in miR-6766-3p mimic-treated activated LX2 cells compared to mimic NC-treated group. In addition, after treatment with an inhibitor of miR-6766-3p, the above effects of activated LX2 cells were reversed. These findings indicated that miR-6766-3p had important effect on some characteristics of LX2 cells. We found that miR-6766-3p repressed mRNA expression of TGFβRII and its downstream signal transduction proteins, such as p-SMAD2/3 and SMAD4 in activated LX2 cells. These phosphorylated SMADs failed to form homologous oligomers, so they could not enter the target LX2 cells to regulate the transcriptional activity of profibrogenic genes [43–45]. On the other hand, ERK1 and P38 MAPK, two upstream modulators of SMADs [23, 33], have also been reported to regulate translocation of SMAD2/3/4 into nucleus and phosphorylation of SMAD2/3, which in turn increase the expression of collagen I and promote the deposition of ECM [23, 33, 46]. These data are consistent with our results that, the expression of SMAD4, ERK1 and P38 MAPK were markedly reduced in miR-6766-3p mimic-treated cells, and such reduction was observed to be weakened in miR-6766-3p inhibitor-treated activated LX2 cells. Here, we confirmed that cell proliferation, chemotaxis and profibrotic phenotypes of activated LX2 cells could be attenuated by miR-6766-3p mimic. To further determine whether TGFβRII was the unique target mediating the activity of miR-6766-3p, relevant validation experiment with TGFβRII KD LX2 cells were performed to characterize the functionality of TGFβRII in TGFβRII/SMADS pathway. Our results showed that knockdown of TGFβRII mimicked the roles of miR-6766-3p, further demonstrating that TGFβRII was the primary functional target of miR-6766-3p in treating chronic liver fibrosis. Notably, in aforementioned results of the entire work, the anti-fibrotic effects induced by 3D-Exos were more significant than those by 2D-Exos, it is believed that the higher level of miR-6766-3p expression in 3D-Exos represents an important factor. In this study, our results provided the further insights into hESC-Exosomes’ cargo and antifibrotic gene regulation in LX2 cells. The contributions from other miRNAs in hESC-Exosomes, alone or in combination, to the antifibrogenic effects could not be excluded. The complete miRNA content of hESC-Exosomes and its underlying mechanism of antifibrotic function should be further explored. In summary, we have tentatively identified a novel mechanism by which 3D-Exo rescue liver injury via antifibrotic miR-6766-3p.

## Conclusions

Our findings demonstrated that the beneficial effect of 3D hESC-Exosome was tied to delivery of miR-6766-3p to LX2 cells, which inactivated SMADs signaling by decreasing TGFβRII expression through targeting TGFβRII 3ʹUTR, which in turn, attenuated stellate cell activation and suppressed the progression of liver fibrosis. This is the first report that 3D hESC-Exosomes exhibit the anti-fibrotic effect on alleviating liver fibrosis in vitro and in vivo, and these findings represent a significant step towards developing 3D hESC-Exosome-derived miR-6766-3p as a novel anti-fibrotic therapeutic for treating chronic liver disease.

## Methods

### Cell culture

hESC was generated in Dr. Jinsong Li’s laboratory, and cultured as previously described [47]. Human hepatic stellate cells (LX2 cells) was purchased from Procell (Life Science & Technology Co,. Ltd) in Wuhan, China, and cultured maintained as described in protocol from the provider.

### Liver fibrosis model

All mouse experiments were performed according to our experimental protocols approved by the Guangzhou Committee for the Use and Care of Laboratory Animals, and by the Animal Ethics Committee of South China University of Technology University (Approval number: 2019073). Male ICR mice (8–10 weeks old) were purchased from Hunan SJA Laboratory Animal Co, Ltd (Changsha, China), and liver fibrosis was established as previously described [48].

### Statistics

Statistical analysis was performed using Student t test. Comparison of two or more groups is performed by 1-way ANOVA or 2-way ANOVA with Bonferroni post hoc test. P < 0.05 is considered statistically significant. Error bars represent SEM. Statistical analysis is performed using Graph Pad prism v 5.0 software.

## Supplementary Information

**Additional file 1. **Extra materials and methods, additional figures and antibodies used in this study shown.

## Data Availability

All data generated or analyzed during this study are included in this published article.

## Abbreviations

α-SMA: α-Smooth muscle actin
CCL_4_: Carbon tetrachloride
CFSE: 5(6)-Carboxyfluorescein diacetate succinimidyl ester
HE: Haematoxylin and eosin
HSCs: Hepatic stellate cells
CCK-8: Cell Counting Kit-8
TUNEL: TdT-mediated dUTP Nick-End Labeling
Smad2: Recombinant Mothers Against Decapentaplegic Homolog 2
p-Smad2: Phosphatase-Smad2
Smad3: Recombinant Mothers Against Decapentaplegic Homolog 3
p-Smad3: Phosphatase-Smad3
Smad4: Recombinant Mothers Against Decapentaplegic Homolog 4
Erk1: Recombinant Extracellular Signal Regulated Kinase 1
Erk2: Recombinant Extracellular Signal Regulated Kinase 2
P38 MAPK: Mitogen-activated protein kinases
