# Supplementary material for: 3D hESC exosomes enriched with miR-6766-3p ameliorates liver fibrosis by attenuating activated stellate cells through targeting the TGFβRII-SMADS pathway
Source: J Nanobiotechnology. 2021 Dec 20;19:437. doi: 10.1186/s12951-021-01138-2 (PMC8686281; doi:10.1186/s12951-021-01138-2)
Supplement: Supplementary file 1 — Additional file 1. Extra materials and methods, additional figures and antibodies used in this study shown. [file 12951_2021_1138_MOESM1_ESM.pdf]

Supplementary Materials for

3D hESC exosomes enriched with miR-6766-3p ameliorates liver fibrosis by attenuating activated stellate cells through targeting the TGFβRII-SMADS pathway

Ning Wang[1,2‡], Xiajing Li[2,3‡], Zhiyong Zhong[1,2‡], Yaqi Qiu[2‡], Shoupei Liu[2], Haibin Wu[2], Xianglian Tang[1,2], Chuxin Chen[4], Yingjie Fu[2], Qicong Chen[1,2], Tingting Guo[2], Jinsong Li[5,6], Shuai Zhang[7], Mark A Zern[8], Keqiang Ma[9], Bailin Wang[10], Yimeng Ou[11], Weili Gu[7*], Jie Cao[12*], Honglin Chen[2,13,14,15,16*], Yuyou Duan[2,13,14,15,16*]

1. School of Biomedical Sciences and Engineering, Guangzhou International Campus, South China University of Technology, Guangzhou 510006, P.R. China.

2. Institutes for Life Sciences and School of Medicine, South China University of Technology, Guangzhou 510006, P.R. China.

3. School of Medicine, South China University of Technology, Guangzhou 510180, P. R. China.

4. School of Biology and Biological Engineering, South China University of Technology, Guangzhou 510006, P.R. China.

5. State Key Laboratory of Cell Biology, Shanghai Key Laboratory of Molecular Andrology, Shanghai Institute of Biochemistry and Cell Biology, Center for

Excellence in Molecular Cell Science, Chinese Academy of Sciences, Shanghai 200031, China.

6. University of Chinese Academy of Sciences, Shanghai 200031, China.

7. Department of Gastroenterology and Hepatology, Guangzhou Digestive Disease Center, Guangzhou First People’s Hospital, School of Medicine, South China University of Technology, Guangzhou 510180, P. R. China.

8. Department of Internal Medicine, University of California Davis Medical Center, Sacramento, California 95817, USA.

9. Department of Hepatobiliary pancreatic surgery, Huadu District People's Hospital of Guangzhou, Guangzhou, 510800, P. R. China.

10. Department of General Surgery, Guangzhou Red Cross Hospital, Jinan University, Guangzhou, 510220, P. R. China.

11. Department of general surgery, the First Affiliated Hospital of Guangdong Pharmaceutical University, Guangzhou 510080, P. R. China.

12. Department of general surgery, Guangzhou Digestive Disease Center，Guangzhou First People’s Hospital, School of Medicine, South China University of Technology, Guangzhou 510180, P. R. China.

13. National Engineering Research Center for Tissue Restoration and Reconstruction, South China University of Technology, Guangzhou 510006, P. R. China.

14. Key Laboratory of Biomedical Engineering of Guangdong Province, South China University of Technology, Guangzhou 510006, P. R. China.

15. Key Laboratory of Biomedical Materials and Engineering of the Ministry of Education, South China University of Technology, Guangzhou 510006, P. R. China.

16. Innovation Center for Tissue Restoration and Reconstruction, South China University of Technology, Guangzhou 510006, P. R. China.

‡ Ning Wang, Xiajing Li, Zhiyong Zhong and Yaqi Qiu contributed equally to this work.

*** Correspondence to:**

Yuyou Duan, Ph.D.

Laboratory of Stem Cells and Translational Medicine

Institutes for Life Sciences

School of Medicine

South China University of Technology

No.382 Waihuan East Road, Suite 406

Higher Education Mega Center

Guangzhou 510006, P.R.China

Telephone: +86 20 3939 0970

E-mail: yuyouduan@scut.edu.cn

Honglin Chen, Ph.D.

Laboratory of Stem Cells and Translational Medicine

Institutes for Life Sciences

School of Medicine

South China University of Technology

382 Waihuan East Road, Suite 406

Higher Education Mega Center

Guangzhou 510006, P.R.China

Telephone: +86 20 3939 0970

E-mail: chenhl@scut.edu.cn

Jie Cao, M.D., Ph.D.

Department of general surgery

Guangzhou Digestive Disease Center

Guangzhou First People's Hospital

School of Medicine

South China University of Technology

No.1 Panfu Road

Guangzhou 510180, P. R. China

Telephone: +86 20 8104 8181

E-mail: eycaojie@scut.edu.cn

Weili Gu, M.D., Ph.D.

Department of Gastroenterology and Hepatology

Guangzhou Digestive Disease Center

Guangzhou First People’s Hospital

School of Medicine

South China University of Technology

No.1 Panfu Road

Guangzhou 510180, P. R. China

Telephone: +86 20 8104 8281

E-mail: lili-6423@163.com

This file includes:

Materials and Methods

Figures S1 to S21

Tables S1 to S4

Methods

hESC Culturing

The hESC line was cultured in 2D and 3D conditions respectively, brifly, hESCs dissected from culture condition with feeder cells [47] were moved to the Matrigel-coated plate for 2D culture and the ultra-low attachment plate (Corning) for 3D culture, and cultured with serum-free mTesR1 medium (STEMCELL).

Exosome Isolation

2D-Exo and 3D-Exo were isolated as previously described [20]. Supernatants were collected from hESC culture in mTesR1 medium without FBS and subsequently subjected to sequential centrifugation steps at 500 g for 10 minutes to remove cells, at 2000 g for 20 minutes to remove apoptotic bodies, and at 5000 g for 30 minutes to remove cell debris. The resulting supernatant was then filtered using 0.22 μm filters (Millipore, Merck, Germany), and hESC-Exosomes were harvested by centrifugation at 130000 g for 2 hours in a SW32 Ti rotor (Beckman Coulter, L-100XP Ultracentrifuge, CA). The pellet was resuspended in PBS and subsequently ultracentrifuged at 130000 g for another 2 hours to remove the contaminating proteins.

Transmission Electron Microscope (TEM)

TEM (Talos F200C, Thermo Fisher, MA) was performed at 200 kV to visualize and examine the morphology of hESC-Exosomes. Samples were deposited on copper grids covered with a carbon support film (Zhongjingkeyi Technology, Beijing, China)

and dried for 2 minutes at room temperature. The excess fluid was removed with a piece of filter, and the samples were negatively stained with 2% uranyl acetate for 30 seconds. Finally, these samples were air-dried for 60 minutes, and images were captured.

Nanoparticle Tracking Analysis

The size, concentration, and zeta potential of hESC-Exosomes were determined using NTA (Particle Metrix, Germany). The parameters of the measurement were set using 100 nm polystyrene-latex beads as standards. hESC-Exosomes were prepared by the dilutions at 4000- and 2000-folds using distilled deionized water respectively, to achieve the objects at the number between 20 and 100 per frame. Each sample was measured in triplicate at the camera setting with an acquisition time of 60 seconds.

Western Blot

The protein concentrations were determined using a bicinchoninic acid (BCA) protein assay reagent (Pierce, Rockford, IL) according to the manufacturer's instructions. hESC-exosomes or the cells were lysed with ice-cold lysis buffer (150 mM NaCl, 50 mM Tris−Cl (pH 7.5), 1 mM EDTA, 1% NP-40, 0.1% SDS, 0.25% sodium deoxycholate, 1 mM PMSF, 1 mM β-glycerophosphate, 1 mM NaF, 1 mM Na3VO4, and protease inhibitor cocktail tablets (Roche Molecular BioChemicals, Indianapolis, IN). Aliquots containing 30 μg of protein were mixed with loading buffer, followed by denaturation at 95 °C for 5 minutes. Proteins were separated by 12% sodium dodecyl sulfate polyacrylamide gel electrophoresis (SDS-PAGE; Invitrogen, Carlsbad,

CA) and transferred to polyvinylidene difluoride membranes (PVDF) (Millipore, Watford, UK). The membranes were blocked with 5% non-fat milk in tri-sec-buffered saline/ Tween-20 (TBST) buffer (20 mM Tris−HCl, pH 7.6, 136 mM NaCl and 0.1% Tween-20), and then probed overnight with primary antibodies, then the membranes were incubated with horseradish peroxidase-conjugated secondary antibodies for 2 hours at room temperature. Protein signals were visualized using the West Pico Chemiluminescent Substrate Kit (Pierce, Rockford, IL). The primary and secondary antibodies were listed in Supplemental Table S4.

hESC-Exosome Labeling

Purified 2D-Exo and 3D-Exo were labeled with PKH26 Red Fluorescent Cell Linker Kit according to the manufacturer's protocol. The extracted hESC-exosomes and lipophilic red fluorescent dye PKH26 (4 μM) (Sigma, MIDI26) were fully mixed and incubated at 37 °C for 30 minutes. After incubation, the mixture was sucked into a 100 KDa MWCO ultra-filtration centrifuge tube, 10 times the volume of PBS, was gently blown and mixed, centrifuged at 4 °C for 30 minutes, the unbound PKH26 dye was removed, the concentrated liquid in the ultra-filtration centrifuge tube was collected, and the bacteria were removed by a 0.22 μm filter. The filtered concentrated liquid was moved into the new EP tube and preserved at 4 °C.

Activation of LX2 Cells and Treatment with hESC-Exosomes

In the logarithmic proliferation phase of LX2 cells, the cells were digested and subcultured by trypsin. 1.25×10^5 cells per well were seeded in the six-well plate, and

the microslide treated with polylysine were placed in the dish pool. When LX2 cells were adherent to the wall (> 6 hours), TGF-β1 (TGFβ, Peprotech, 100-21-50) was added to induce activation of LX2 cells at the final concentration of 10ng/mL for 48 hours. 20 μg of 2D-Exo and 3D-Exo labeled with PKH26 (4 μM; λ_{ex} = 565 nm, λ_{em} = 594 nm) were respectively added. The same volume of PBS was added to the control group. The cells were examined 24 hours after treatment.

Observation of Intracellular Localization of Exosomes in Cells by Laser Scanning Microscopy (LSM)

LX2 cells were cultured in 6-well plates with the cell number at 1×10^5 cells per well and adhered overnight. PKH26-labeled 2D-Exo and 3D-Exo were respectively added into LX2 cells and incubated at 37 °C in dark for 24 hours, discarded culture medium, and washed with PBS for 3 times. Afterward, the cells were fixed using 4% paraformaldehyde (PFA) (Sangon Biotech, Shanghai, China) for 10 minutes, and then washed with PBS three times. The fixed cells were incubated with primary antibody against α-Tubulin (CST, 3873s) overnight at 4 °C, and then incubated with Alexa Fluor 488-conjugated goat anti-rabbit IgG (CST, 4412s). The cell nuclei were counterstained with 4, 6-diamidino-2-phenylindole (DAPI) for 10 minutes and imaged using LSM (Ni-E-A1, Japan).

Determination of Intracellular Calcium Concentration

Differently treated-LX2 cells were washed three times with PBS, and incubated with Fluo-3 AM (Beyotime, S1056) at 5 mM at 20-37 °C for 10-60 minutes, then washed

for 3 times with PBS. Fluo-3 fluorescence was detected by flow cytometry to determine the changes in intracellular calcium concentration.

Terminal Deoxynucleotidyl Transferase-Mediated dUTP Eick-End Labeling (TUNEL) assay

A TUNEL assay was performed to evaluate the apoptotic cells of liver tissues. The tissues were fixed with 4% PFA and post-fixed in a pre-chilled solution containing ethanol and acetic acid mixed at 2:1 for 5 minutes at −20°C. After fixation, samples were processed according to the manufacturer's instructions (Beyotime, C1086) and imaged using LSM (Ni-E-A1, Japan). Quantitative analysis was accomplished using ImageJ software (National Institutes of Health, USA).

Wound Healing Assay

5×10^5 LX2 cells were seeded in the six-well plate, when the cells were adherent (> 6 hours), TGFβ was added to induce activation of LX2 cells at the final concentration of 10 ng per mL for 48 hours, 20 μg of 2D-Exo and 3D-Exo were respectively added and incubated with LX2 cells. After culturing for 24 hours, the LX2 cells were scratched with a pipette tip, and the cells were then washed three times with PBS to remove the scraped cells, finally, serum-free medium was added for culturing with 5% CO_2 at 37 °C. The scratched areas were imaged and measured using NIH Image J at 0 hour and 24 hours (n = 3)

CFSE assay

LX2 cell proliferation rate in four different groups were also determined using flow cytometric analysis with a CellTrace CFSE Cell Proliferation Kit ($1x10^6$ cells/ml in 5μM of CFSE, 0.1% BSA in PBS for 10 minutes, Biolengend). The reaction was terminated by the addition of 10 volumes of cold RPMI 1640 with 10% FBS. Finally, the CFSE fluorescent staining was analyzed by flow cytometry. CFSE assays were performed in triplicate. When cells undertook divisions, the CFSE was distributed to daughter cells equally and CFSE fluorescence decreased in daughter cells. Briefly, the progressive loss of CFSE fluorescence is a hallmark of the cell proliferation.

Cell Viability Assay

Briefly, LX2 cells in the above groups were seeded at 5,000 cells per well in a 96-well plate and allowed to settle overnight. Cell proliferation of LX2 cells was measured using a Cell Counting Kit-8 (Beyotime, C0037). CCK-8 reagent was added to each well and incubated for 1, 2, 4 and 6 hours respectively. Absorbance was read at 450 nm and recorded using a microplate spectrophotometer.

Isolation and Charactwerization of Mouse Primary Hepatic Stellate Cells

Primary mouse hepatic stellate cells (mHSCs) were isolated from ICR mice as described previously [27], and freshly-isolated primary mHSCs were cultured in DMEM supplemented with 10% FBS. mHSCs were characterized by Oil red staining and immunofluorescent staining for the deposit of the lipid droplets and cell type specific marker Desmin.

Teratoma Formation Assay

2D-hESCs, 3D-hESCs and their respective hESC-Exosomes were collected, and approximately 1×10^6 cells of hESCs and 100 ug of hESC-Exosomes were subcutaneously injected into immunodeficient NDG mice (around 5 weeks old) (Biocytogen Co., Ltd, Beijing, China). 6 NDG mice were treated in each group. Teratomas generally developed within 6-8 weeks, and the animals were sacrificed before the tumor sizes exceeded 1.5 cm in diameter. The teratomas were then embedded in paraffin and processed for hematoxylin and eosin (HE) staining.

Immunofluorescence Staining

The cells were fixed in 4% PFA at room temperature for 15 minutes and blocked with blocking buffer that contained 0.2% Triton X-100 (Sigma-Aldrich, T8787) and 3% normal goat serum (Jackson Immuno Research, 017-000-121) in PBS at room temperature for 45 minutes, then the cells were incubated with primary antibodies at 4 °C overnight. Next day, primary antibodies were replaced with secondary antibodies conjugated with fluorescences (CST, 7074s and 7076s) after washing with PBS, and the cells with antibodies were incubated at room temperature for 1 hour. The nuclei were stained with DAPI. The primary and secondary antibodies were listed in Supplemental Table S4.

Flow cytometric Analysis

Single cell suspensions from 2D-hESCs and 3D-hESCs culturing were prepared using GCDR (STEMCELL, 07174), washed in PBS, then resuspended in 0.1% BSA in PBS. Anti-TRA-1-81-PE and anti-SSEA4-PE (STEMCELL Technologies) and PE

Mouse IgG1 κ Isotype Control (BD) were added to cell suspensions to incubate at 4 °C for 30 minutes in the dark. Intracellular staining for OCT4 were performed according to the manufacturer's instructions (STEMCELL Technologies). Cells were examined by flow cytometry. Data were analyzed using Flowjo software (Flowjo, Ashland, USA).

Detection of Serological Markers and Liver Injury in Fibrotic Mice

4 weeks after intravenously receiving CCL_4 and 56% alcohol gavage, the mice fasted 12 hours before they were sacrificed. Serum was collected by centrifugation of eyeball blood for testing Aspartate Transaminase (AST) (Solarbio, BC1560), Alanine transaminase (ALT) (solarbio, BC1550), albumin (ALB) (Ek-Bioscience, Ek-M20152) and Total bilirubin (TBIL) (GTX， YS01266B) using an automatic biochemical analyzer. Liver tissue samples were retained separately, and fixed in 4% PFA for histopathological analysis. In addition, the remaining liver tissues were first placed in liquid nitrogen for quick cryopreservation and then transferred into -80 °C for storage.

Two-photon excited fluorescence (TPEF) imaging of PKH26-lebeled hESC-Exosomes Distributed in Various Organs of Mice in vivo and ex vivo

The hepatic fibrosis model mice were injected intravenously with 100 μg of PKH26-labeled 2D-Exo and 3D-Exo as well as equal volume PBS (control group). The mice anesthetized at differetn time points, and tissue samples from mice were imaged to collect fluorescence in stack scan mode by TPEF (Ni-E-A1RMP) with a

laser at wavelengths of 1080 nm for PKH26 imaging. During the imaging process, each sample were scanned continuously (a frame of a 2D image) for PKH26 simultaneously at a speed of 10 second per frame. The exposure time of the samples was limited within 30 minutes. The intensities of the signals were analyzed using Image J software.

RNA Extraction and Quantitative Polymerase Chain Reaction (qPCR)

Total RNA was extracted from cells and tissues using RNAiso Plus (Takara, 9109) according to the manufacturer's manuals, MiRNAs of 2D-Exo and 3D-Exo were isolated using the SeraMir Exosome RNA Purification Column Kit (EZBioscience, EZB-exo-RN1). For mRNA detection, complementary DNA (cDNA) was synthesized from 500 ng of total RNA using a PrimeScript™ RT Master Mix (Takara); for miRNA expression analysis, 500 ng total RNA was reverse-transcribed into cDNA using Mir-X™ miRNA First Strand and Synthesis kit (Takara) as described by the manufacturer's protocol. All qPCR experiments were performed on a real-time PCR machine (ABI, ABI7500) with the QuantiTect SYBR Green PCR Kit (Takara) and specific primers of the genes of interest, and amplification efficiencies were checked by standard curves. Gene expression was quantified by the comparative cycle threshold (Ct) method. The relative amounts of target gene expression were determined by subtracting the Ct values of these genes from the Ct value of the housekeeping gene GAPDH and the abundance of U6 which was used as an internal control for comparison of relative changes in miRNA among different groups (△Ct).

The data were presented as $2^{-(\Delta\Delta CT)}$. The primer sequences used in this study were listed in Supplemental Table S1.

Histology, Immunohistochemistry (IHC) and Immunofluorescence (IF) Assays

On day 7, 14, 21 and 28 after administration of PBS, 2D-Exo and 3D-Exo, all mice were sacrificed, and the livers were isolated. Liver tissues were immediately fixed in 4% PFA overnight. Afterward, these tissues were embedded in paraffin and sectioned with a thickness of 5 μm, followed HE or Masson's trichrome staining, or ICH assay. The slices obtained were examined with an optical microscope (Nikon, Japan). Furthermore, another fraction of liver tissue was embedded into OCT compound (Sakura Finetek, Japan) and cut into micron-thick frozen sections with the thickness at 10 μm for IF staining. In brief, the sections for ICH and IF staining were incubated with primary antibodies, and then incubated with secondary antibodies. Cell nuclei in IF staining were counterstained with DAPI. Image J software was used to binarize the immunofluorescence images taken as previously described.[25] The numbers of positive cells were counted by blinded investigators in 6 randomly selected areas. The primary and secondary antibodies were listed in Supplemental Table S4.

miRNA Microarray and Data Analysis

Agilent Feature Extraction software (version 11.0.1.1) was used to analyze acquired array images. Quantile normalization and subsequent data processing were performed using the GeneSpring GX v14.9 software package (Agilent Technologies). After quantile normalization of the raw data, miRNAs that at least 3 out of 6 samples have

flags in Detected ("All Targets Value") were chosen for further data analysis. Differentially expressed miRNAs with statistical significance between the two groups were identified through Volcano Plot filtering. Differentially expressed miRNAs between the two samples were identified through Fold Change filtering. Hierarchical Clustering was performed using the R scripts. Fold change ≥ 2.0 or ≤ 0.5 and $P<0.05$ were the threshold to filtrate differentially expressed miRNAs. The primer sequences used in this study were listed in Supplemental Table S2 and S3.

Target Prediction

Microarray analysis of miRNAs from 2D-Exo and 3D-Exo, including sample labeling, array hybridization, data collection, and normalization, was performed by Kang Cheng Bio-tech (Shanghai, China) according to the Agilent miRNA Microarray System with miRNA Complete Labeling and Hyb Kit protocol (Agilent Technology). miRDB V5.0, miRNAMap and TargetScan 7.2 software were used to predict target genes of candidate miRNAs.

Dual Luciferase Reporter Assay

For identifying the binding site between miR-6766-3p and its target TGFβRII, the 3' untranslated regions (UTRs) of TGFβRII including wild-type (wt) or mutant type (mut) were respectively cloned downstream of the Renilla luciferase (Rluc) gene as fusion gene driven by SV40 promoter in pSI-check2 dual luciferase repoter vector in which the firefly luciferase (fluc) gene is driven by HSV TK promoter (http://www.hanbio.net, GenePharma Co., China), and 293T cells as the recipient

cells were used for luciferase activity assays. Briefly, cells were first transfected with the recombinant pSI-check2 vector containing the wt or mut TGFβRII 3' UTR, then the cells were co-transfected with miR-6766-3p mimics and miR-6766-3p mimics negative control. The protein translation of Rluc from transcribed mRNA of fusion gene depends on the binding between miR-6766-3p and its target site, and the fluc is normally expressed and used as the calibrator. The relative activity Rluc/fluc reflects the expression change of Rluc by miR-6766-3p mimics. The luciferase activities were detected using the Dual-Luciferase Reporter Assay System (Promega, U.S.A.) according to the manufacturer's protocol.

Validation of Target miRNA

2×10^5 LX2 cells were added into each well of 24 well plate (>6 hours), primed with TGFβ for 48 hours and then transfected with miR-6766-3p mimics (50 nM), inhibitors, negative control mimics and negative control inhibitors (100 nM) (Ribobio, Guangzhou) using ribo FECT CP Transfection Kit (Ribobio, Guangzhou) as per manufacturer's instructions for 24 hours. LX2 cells from each group were collected for subsequent analysis.

RNA Interference and Transfection

The LX2 cells were transfected with siRNAs against TGFβRII and negative control siRNA (Ribobio, Guangzhou). The LX2 cells were transfected with siRNAs using ribo FECT CP Transfection Kit (Ribobio, Guangzhou) as per manufacturer's instructions for 72 hours, and qRT-PCR and western blotting were performed to evaluate the knockdown efficiency of siRNAs.

Verification of Targeting Effect of TGFβRII

2×10^5 LX2 cells in each well of 24 well plate were transfected with siRNAs against TGFβRII and negative control siRNA (Ribobio, Guangzhou), without time interval, followed by TGFβ treatment for 48 hours and then transfected with miR-6766-3p mimics (50 nM) (Ribobio, Guangzhou) using ribo FECT CP Transfection Kit (Ribobio, Guangzhou) as per manufacturer's instructions for 24 hours. LX2 cells from each group were collected for subsequent analysis.

Reference:

20. Hou, D. R. et al. Derivation of Porcine Embryonic Stem-Like Cells from In Vitro-Produced Blastocyst-Stage Embryos. Sci Rep-Uk 6 (2016).

27. Mannaerts I, Leite SB, Verhulst S, Claerhout S, Eysackers N, Thoen LF, Hoorens A, Reynaert H, Halder G, van Grunsven LA. The Hippo pathway effector YAP controls mouse hepatic stellate cell activation. J Hepatol. 2015;63(3):679–88.

47. Zhong, C. Q. et al. Generation of human haploid embryonic stem cells from parthenogenetic embryos obtained by microsurgical removal of male pronucleus. Cell Res 26, 743-746 (2016).

Figure. S1

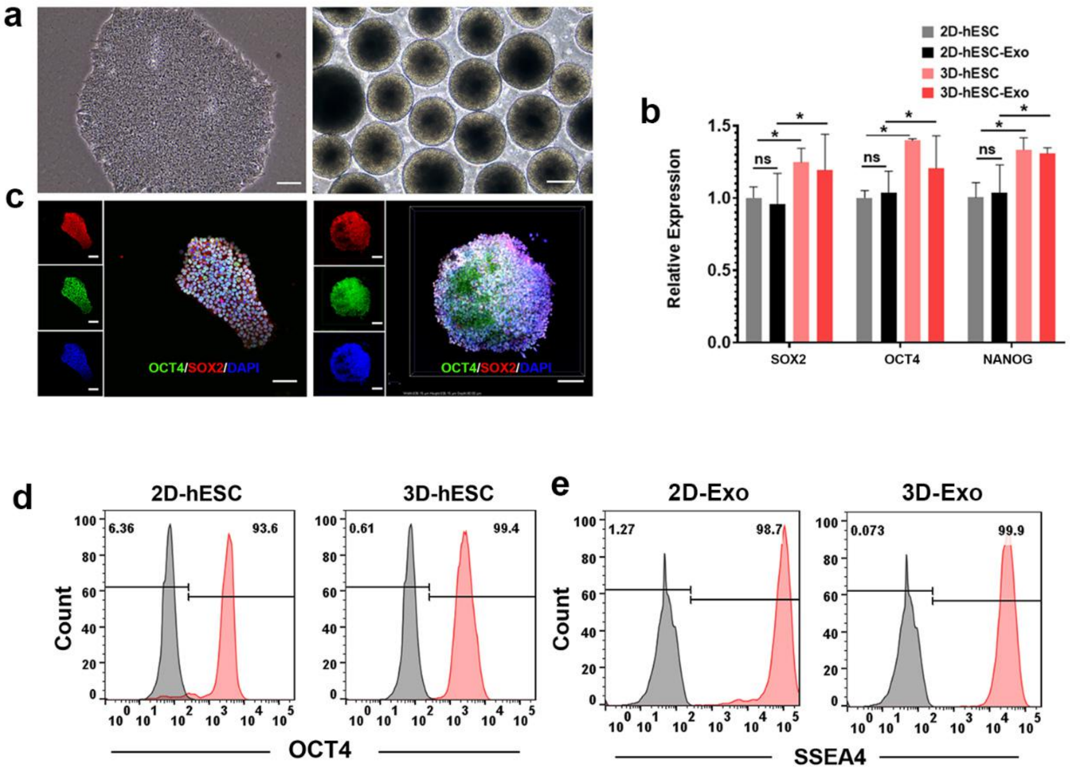

Figure S1. Characterization of pluripotent markers of cultured hESCs.

(a) Morphology of hESCs cultured as monolayer (2D) under feeder cell-free condition (left) and in suspension (3D) to form spheroids (right) (scale bar: 100μm) (b) Real-time qPCR analysis of the expression levels of stemness-related genes in 2D and 3D cultured hESCs, respectively. (c) Immunostaining analysis of OCT4 (green) and SOX2 (red) expression in 2D and 3D cultured hESCs using confocal microscopy. DAPI (blue) was used to localize the nuclei of the cells (scale bar: 40μm). The percentage of $OCT4^{+}$ cells in hESCs (d) and $SSEA4^{+}$ cells hESC-Exosomes (e) was measured by flow cytometry. n=3, Data represent the mean ± SEM. *P < 0.05, **P < 0.01, and ***P < 0.001.

Figure. S2

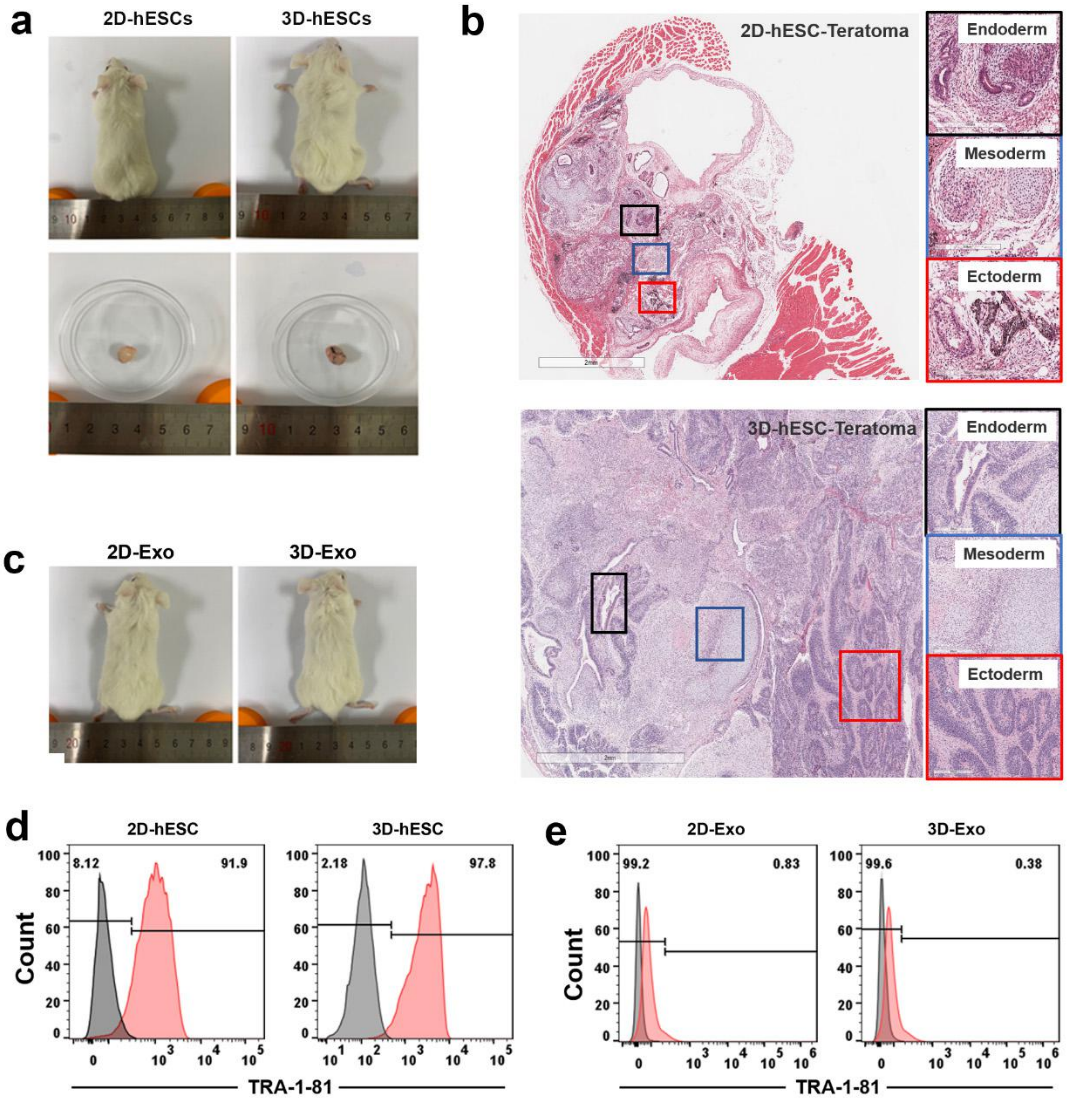

Figure S2. Evaluation of the capacity of hESCs and hESC-exosomes to form teratomas.

(a) Teratomas were formed at day 50 post transplantation of hESCs. (b) H&E staining of teratomas from (a). Three germ layers, mesoderm (immature cartilage), endoderm (digestive glands), and ectoderm (epithelium), were found in teratoma tissues. Original magnification, 10×, Scale bar, 900μm. (c) No teratoma was formed in mice

injected with hESCs-Exosomes. (d-e) The percentage of TRA-1-81$^+$ cells in hESCs (d) and hESC-Exosomes (e) was measured by flow cytometry.

Figure. S3

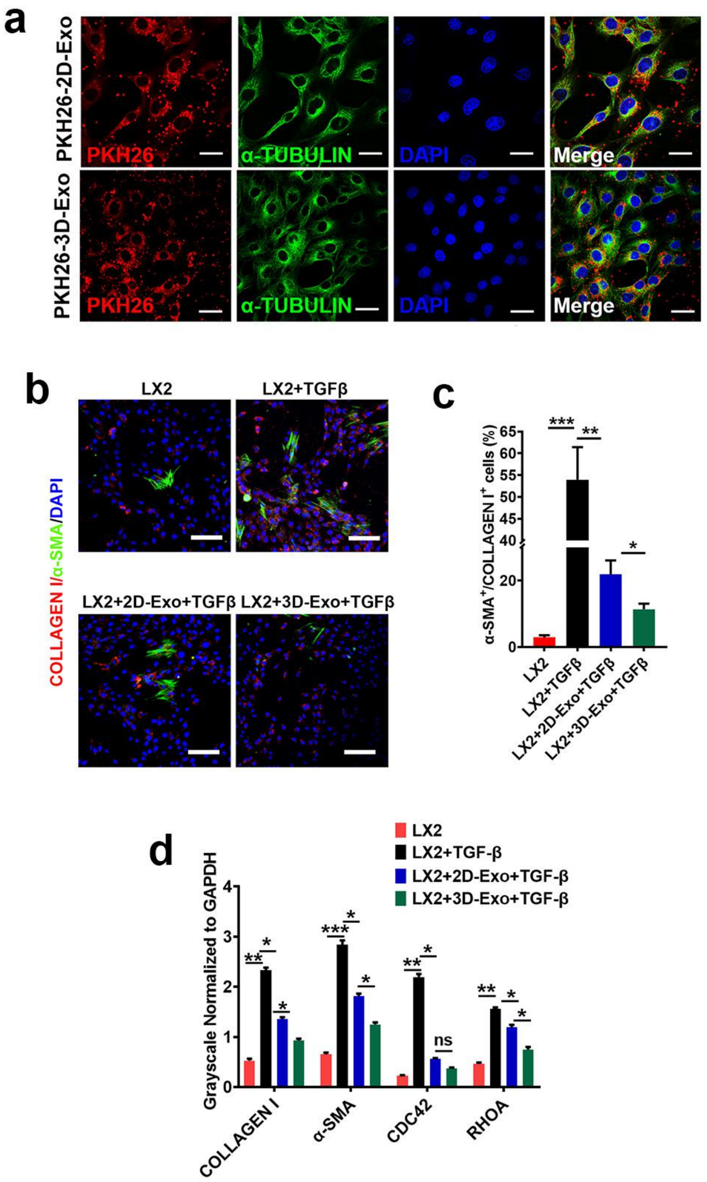

Figure S3. hESC-exosomes internalized by LX2 cells reduced LX2 cells profibrogenic phenotype.

(a) CLSM images of Tubulin immunostaining of internalized PKH26-2D-Exo and PKH26-3D-Exo in LX2 cells. Red: PKH26-2D-Exo and PKH26-3D-Exo. Green:

Tubulin. Blue: DAPI. Scale bars, 40μm. (b) CLSM images of immunostaining for α-SMA and COLLAGEN I in LX2 cells and TGFβ-induced LX2 cells after treatment with 2D-Exo and 3D-Exo. The colours of Green, red and blue represent the staining for COLLAGEN I, α-SMA and nuclei. (Original magnification, 20×. Scale bar, 40μm). (c) Quantification of the percentage of α-SMA^+ and COLLAGEN I^+ cells in (b). (d) According to Western blot analysis shown in Fig. 2d, corresponding densitometry of markers COLLAGEN I, CDC42, α-SMA and RHOA were analyzed in LX2 cells primed with TGFβ and exposed to 2D-Exo and 3D-Exo for 24 hours. GAPDH was used as a loading control. n=3, Data represent the mean ± SEM. *$P < 0.05$, **$P < 0.01$, and ***$P < 0.001$.

Figure. S4

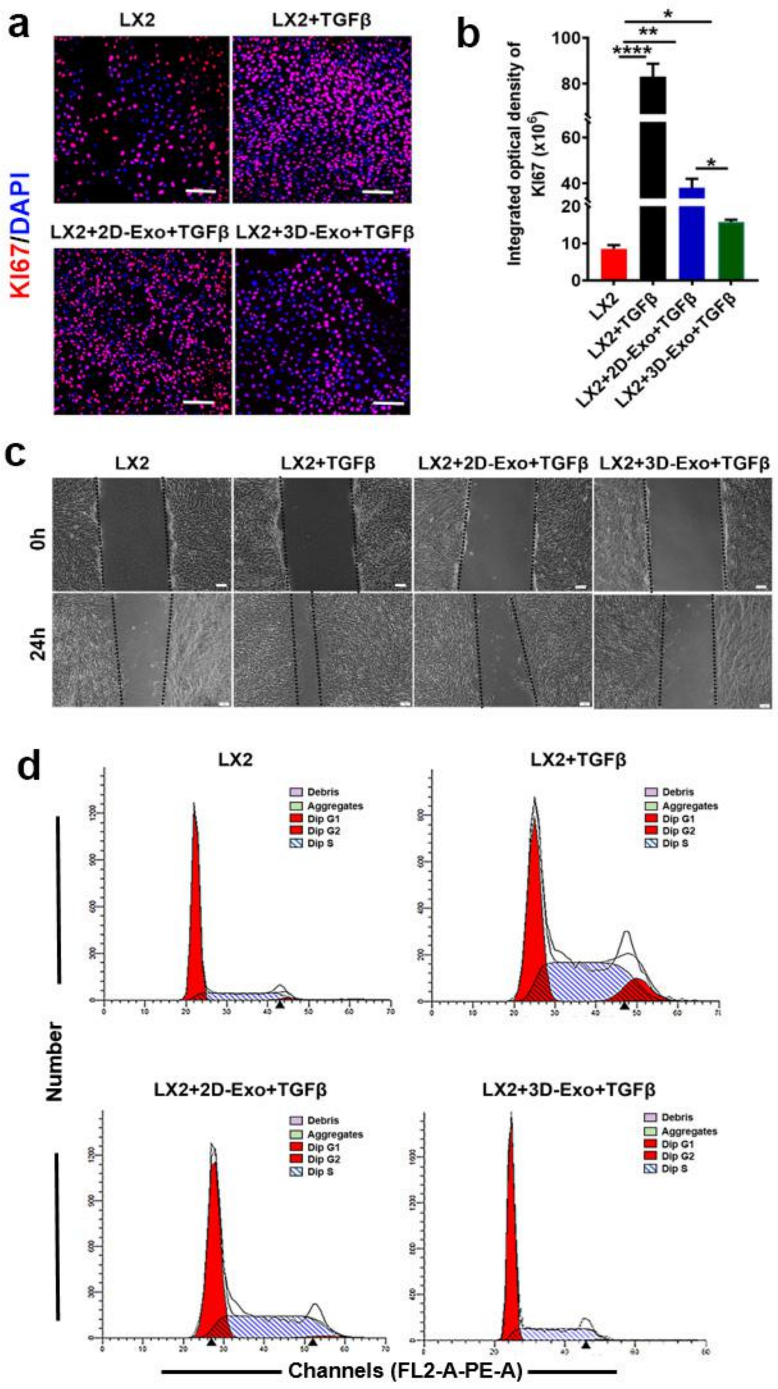

Figure S4. hESC-exosomes modulated proliferation and profibrogenic phenotype of LX2 cells.

(a) Immunostaining of KI67 in LX2 cells and activated LX2 cells with the treatment of 2D-Exo and 3D-Exo. The colours of Red and blue represent the staining for KI67

and nuclei. Scale bar, 100μm. (b) Quantification of integrated optical density of KI67 in LX2 cells primed with TGFβ and exposed to 2D-Exo, 3D-Exo or Exo-free supernatant for 24 hours. (c) Wound recovery rates of LX2 cells and activated LX2 with different treatments, modeled by cell scratch assays (Original magnification, 4×, Scale bar, 100μm). (d) Cell cycle analysis was used to determine the changes of cell cycle, and results showed that the growth of activated LX2 cells exposed to 2D-Exo or 3D-Exo was significantly inhibited, compared with the TGFβ-induced group. n=3, Data represent the mean ± SEM. *$P < 0.05$, **$P < 0.01$, and ***$P < 0.001$.

Figure. S5

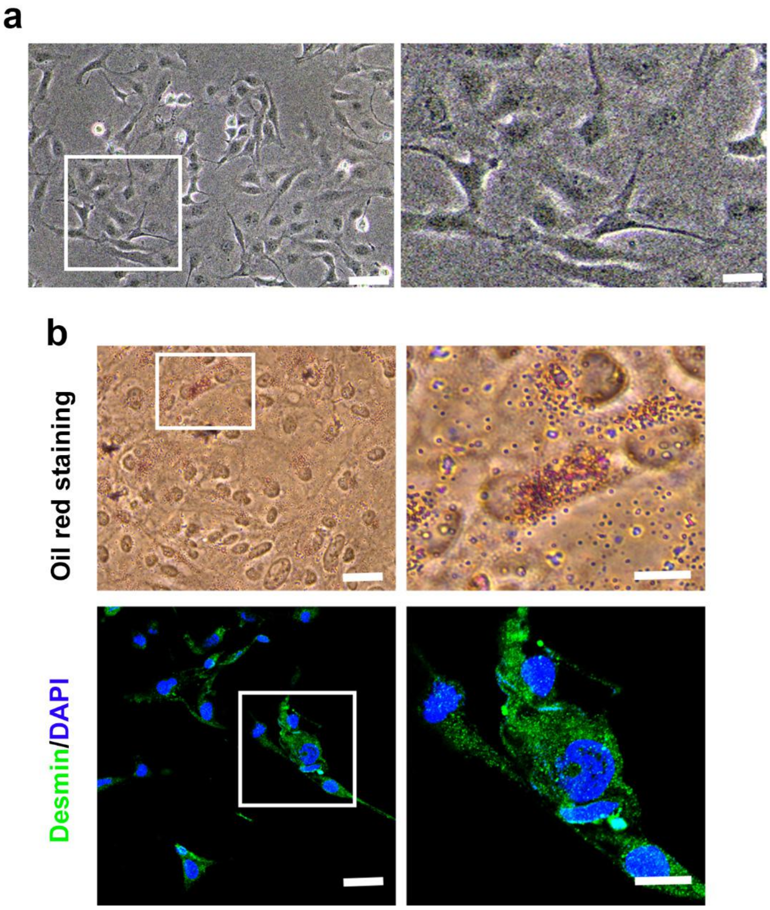

Figure S5. Culture and characterization of mouse primary stellate cells.

(a) Morphology of freshly-isolated mouse primary stellate cells (mHSCs) clutured at 5 days. (left, original magnification, 10×, Scale bar, 100μm; right, original magnification, 20×, Scale bar, 50μm) (b) Oil red staining of mHSCs. The lipid droplets were stained in red. (left, original magnification, 10×, Scale bar, 100μm; right, original magnification, 20×, Scale bar, 50μm) (c) Immunostaining results showed the expression of Desmin in mHSCs. The colors of Green and blue represent the stainings for Desmin and nuclei, respectively. (left, original magnification, 20×, Scale bar, 40μm; right, original magnification, 40×, Scale bar, 20μm)

Figure. S6

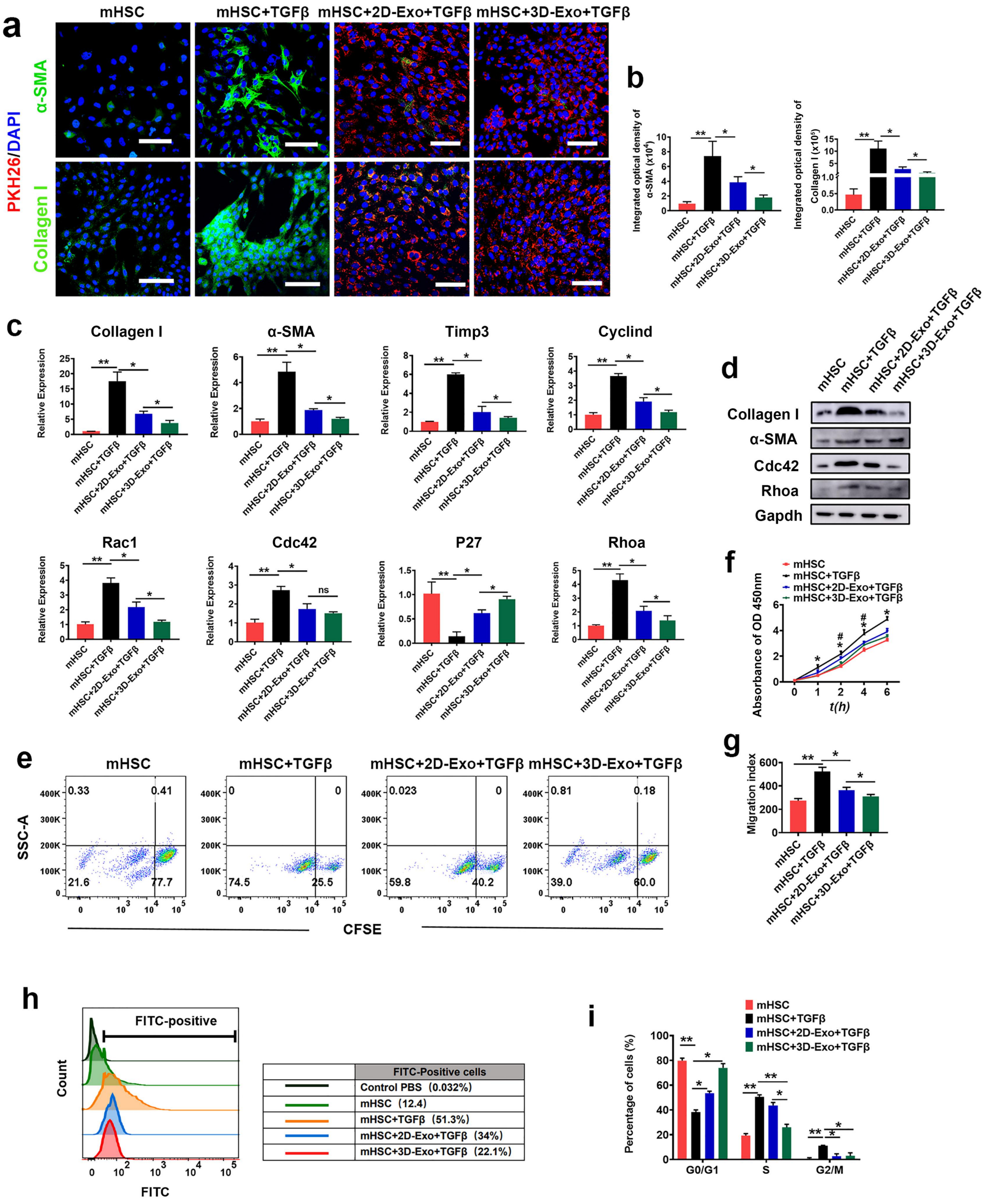

Figure S6. hESC-Exosomes internalized by mHSCs reduced profibrogenic phenotype of mHSCs.

(a) Representative confocal microphotographs of TGFβ-treated mHSCs exposed to PKH26-labeled 2D-Exo and 3D-Exo (red) and EV-free supernatant for 24 hours.

2D-Exo and 3D-Exo inhibited the expression of α-SMA and Collagen I in TGFβ-activated mHSCs. The colors of Green, red, and blue represent the stainings for α-SMA/Collagen I, PKH26 and nuclei, respectively (Original magnification, 20×, Scale bar, 40μm). (b) Quantification of integrated optical density of α-SMA and Collagen I in (a). (c) Quantification of Collagen I, α-SMA, Timp3, Cyclind, Rac1, Cdc42, P27, and Rhoa by qPCR. (d) Representative Western blot analyses of markers Collagen I, α-SMA, Cdc42, and Rhoa in mHSCs primed with TGFβ and exposed to 2D-Exo and 3D-Exo for 24 hours. Gapdh was used as a loading control. (e) Proliferation (as measured by the dilution of carboxyfluorescein succinimidyl ester (CFSE)) of mHSCs and activated mHSCs with different treatments. (f) The proliferative activity of mHSCs in each group were measured by CCK-8 assay at 1h, 2h, 4h and 6h after treatment. (g) Wound recovery rates of mHSCs with different treatment, modeled by cell scratch assays (The images of cell scratch assays were provided in Figs.8c.). (h) Fluo-3 AM fluorescent probes were used to detect the concentration of calcium ions in mHSCs of different treatment groups. (i) Quantification of cell cycle analysis by flow cytometry which was used to measure the growth of mHSCs and activated mHSCs exposed to 2D-Exo or 3D-Exo. Data represent the mean ± SEM. *P < 0.05, **P < 0.01, and ***P < 0.001.

Figure. S7

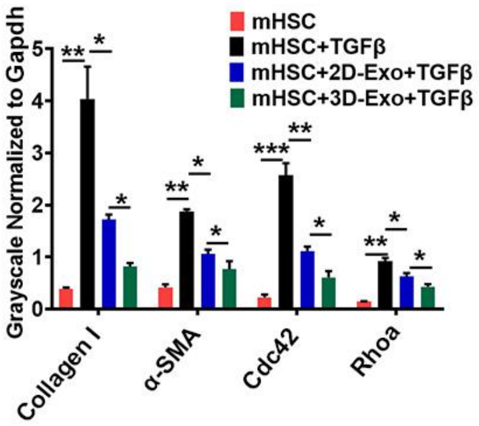

Figure S7. Quantification of protein levles by densitometry.

According to Western blot analysis shown in Figs.6d, corresponding densitometry of markers Collagen I, Cdc42, α-SMA and Rhoa were analyzed in mHSCs primed with TGFβ and exposed to 2D-Exo and 3D-Exo for 24 hours. Gapdh was used as a loading control. n=3, Data represent the mean ± SEM. *P < 0.05, **P < 0.01, and ***P < 0.001.

Figure. S8

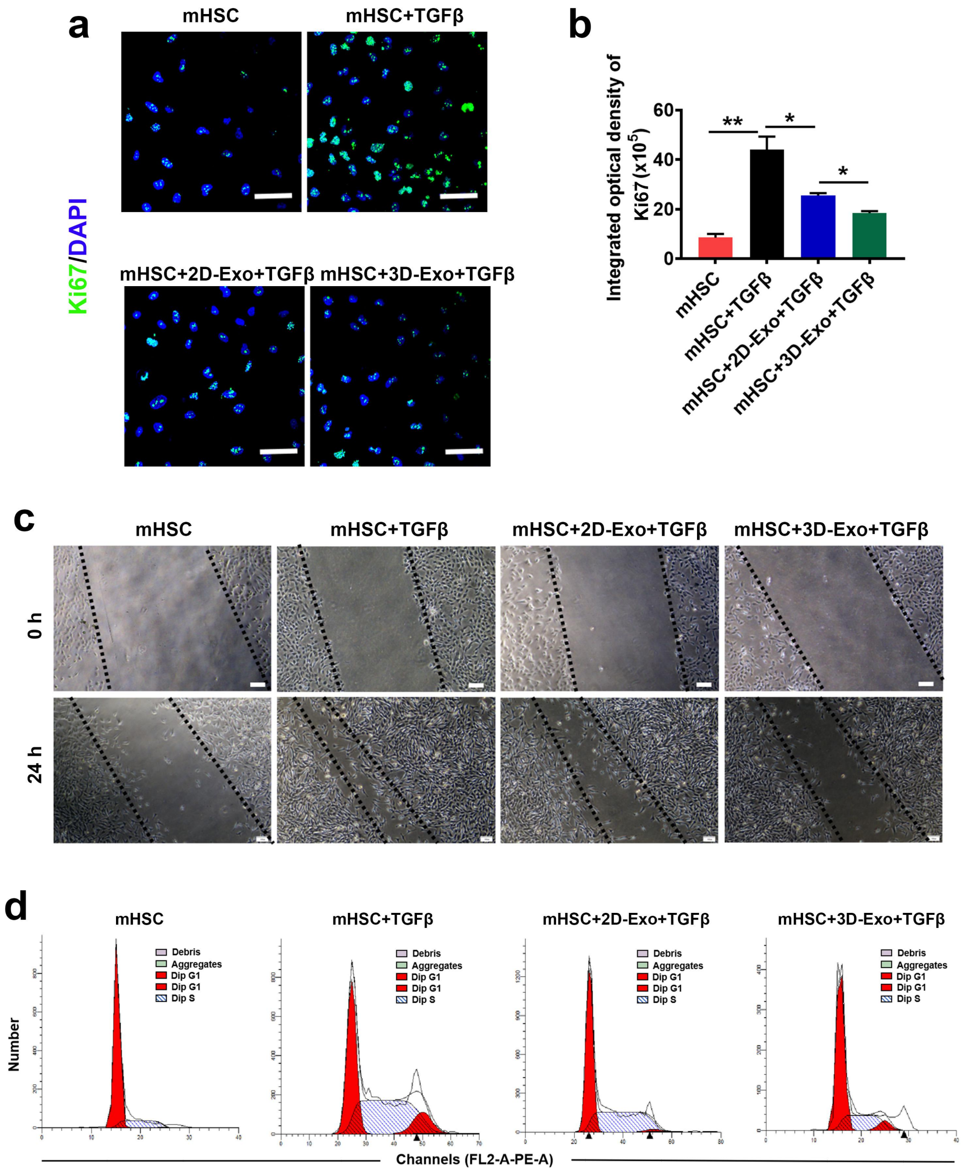

Figure S8. hESC-exosomes modulated proliferation and profibrogenic phenotype of mHSCs.

(a) Immunostaining of Ki67 in mHSCs and activated mHSCs with the treatment of 2D-Exo and 3D-Exo. The colours of Green and blue represent the staining for Ki67 and nuclei. Scale bar, 100μm. (b) Quantification of integrated optical density of Ki67 in mHSCs primed with TGFβ and exposed to 2D-Exo, 3D-Exo or Exo-free

supernatant for 24 hours. (c) Wound recovery rates of mHSCs and activated mHSCs with different treatments, modeled by cell scratch assays (Original magnification, 4×, Scale bar, 100μm). (d) Cell cycle analysis was used to determine the changes of cell cycle, and results showed that the growth of activated mHSCs exposed to 2D-Exo or 3D-Exo was significantly inhibited, compared with the TGFβ-induced group. n=3, Data represent the mean ± SEM. *P < 0.05, **P < 0.01, and ***P < 0.001.

Figure. S9

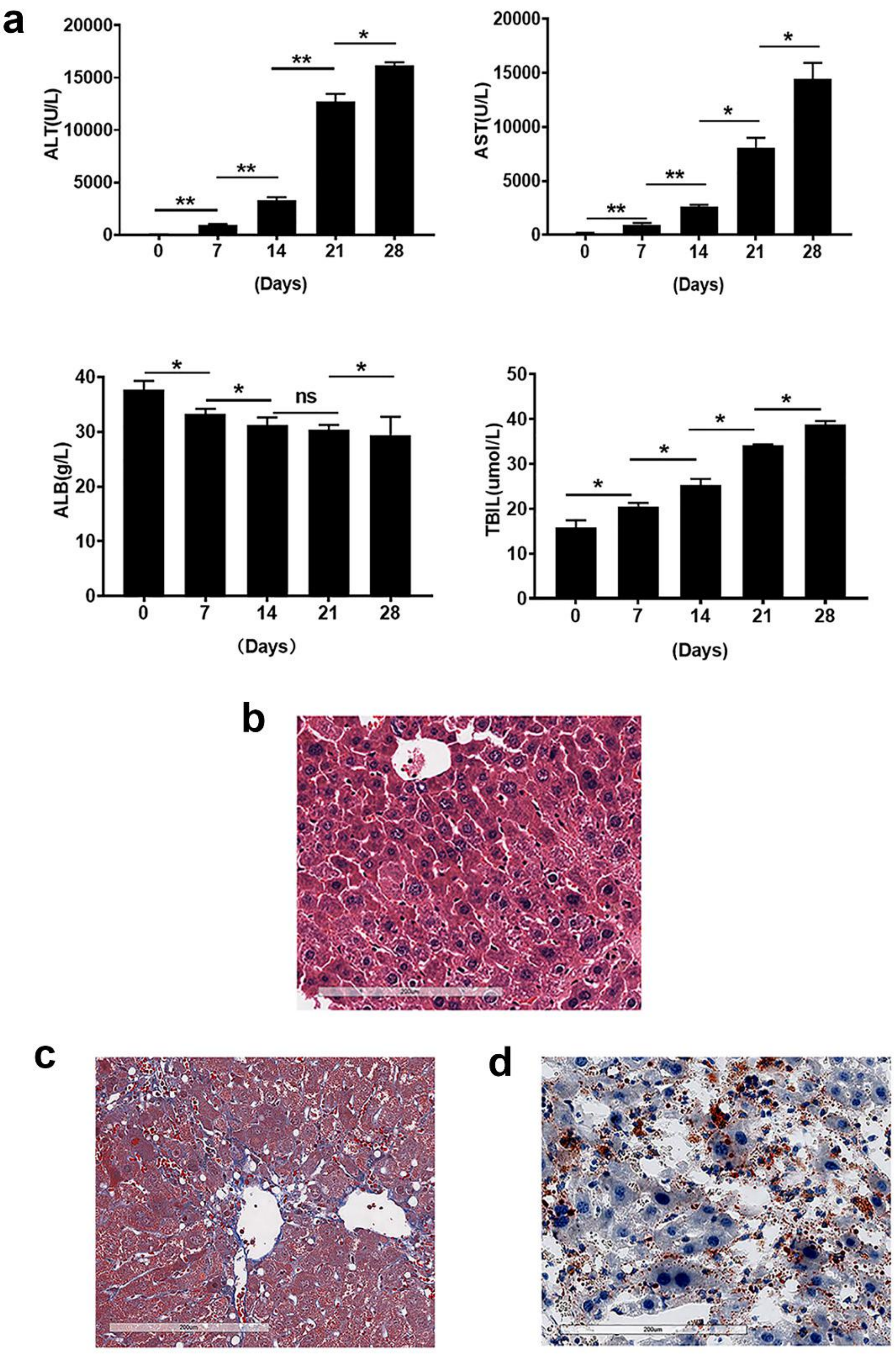

Figure S9. Evaluation of liver fibrosis induced by CCl_4 and alcohol in mice.

(a) Serological analysis of AST, ALT, TBIL and ALB in the serum of mice with induced-liver fibrosis. Employing CCl_4 in conjunction with using alcohol had great

effect on decreasing the serum levels of ALB and increasing the serum levels of AST, ALT and TBIL. (b) Representative H&E staining of livers of the fibrosis mice. (c) Collagen deposition in the livers of the fibrosis mice detected by Masson staining. (d) The accumulation of the lipid in the livers of the fibrosis mice detected by Oil Red staining. n=3, Data represent the mean ± SEM. **P* < 0.05, ***P* < 0.01, and ****P* < 0.001.

Figure. S10

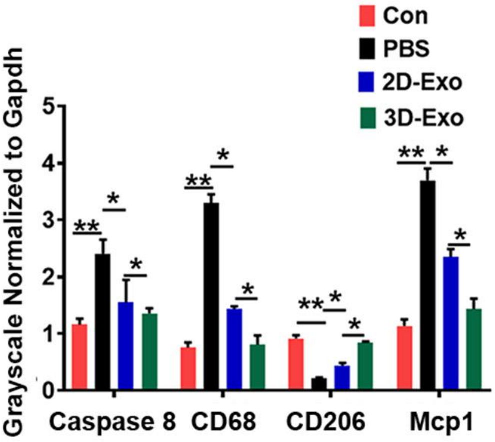

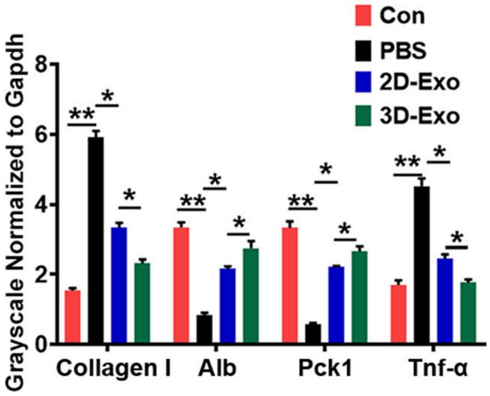

Figure S10. Apoptosis, inflammation and fibrosis could be weaken by hESC-exosomes in the livers of fibrosis mice.

According to Western blot analysis in Fig. 4f, corresponding densitometry for the protein levels of Collagen I, Alb, Pck1, Tnf-α, Caspase 8, CD68, CD206 and Mcp1 were quantitated in the liver tissues of the fibrosis mice with different treatments. Gapdh was used as a loading control. n=3, Data represent the mean ± SEM. *$P < 0.05$, **$P < 0.01$, and ***$P < 0.001$.

Figure. S11

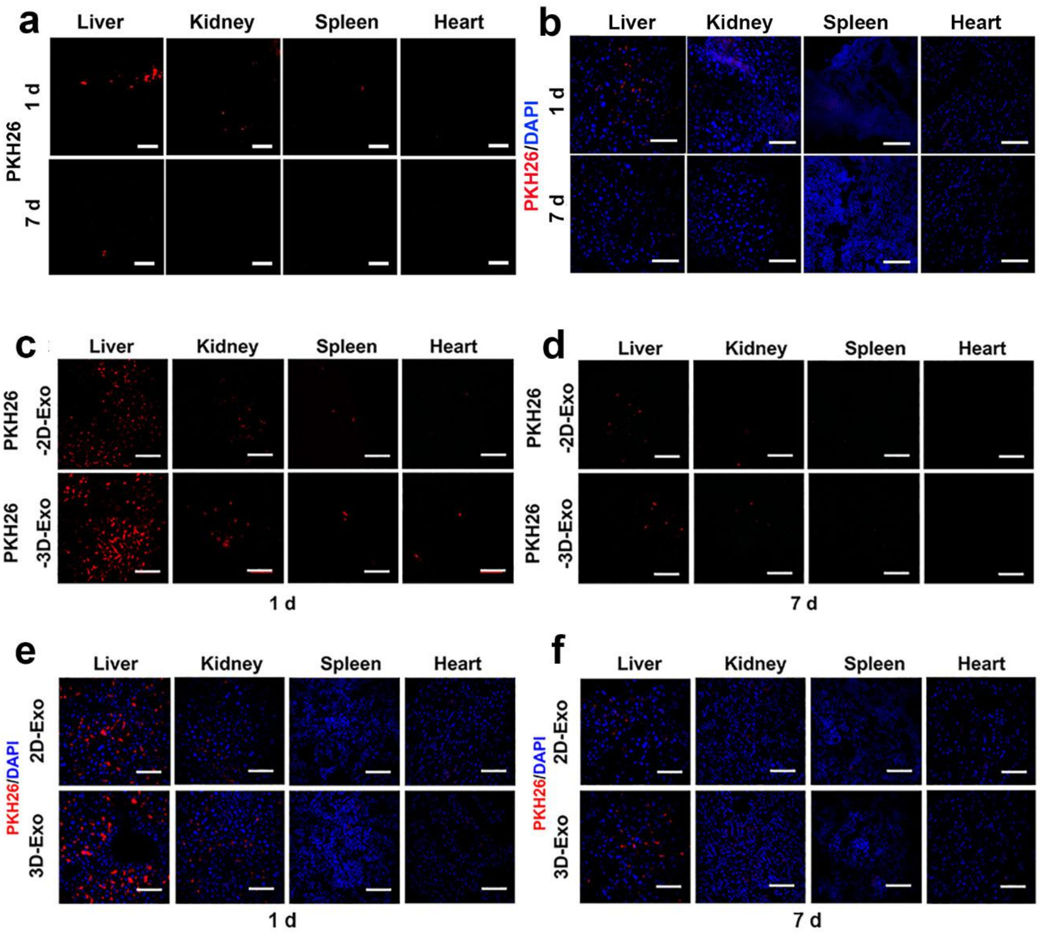

Figure S11. In vivo tracking of PKH26 and PKH lableled-Exos in mice.

(a) *In vivo* TPEF imaging of major organs (liver, kidney, spleen and heart) in PKH26-alone -treated mice on days 1 and 7. (b) *Ex vivo* CLSM imaging of major organs (liver, kidney, spleen and heart) dissected from sacrificed the mice on days 1 and 7. (c-d) The enrichment and metabolism of PKH26 labeled-exosomes in liver, kidney, spleen and heart were imaged using TPEF at 1d and 7d after caudal vein injection (Original magnification, 25×, Scale bar, 40μm). (e-f) *Ex vivo* CLSM imaging of major organs (liver, kidney, spleen and heart) dissected from sacrificed the mice on days 1 and 7. (Original magnification, 20×, Scale bar, 40μm).

Figure. S12

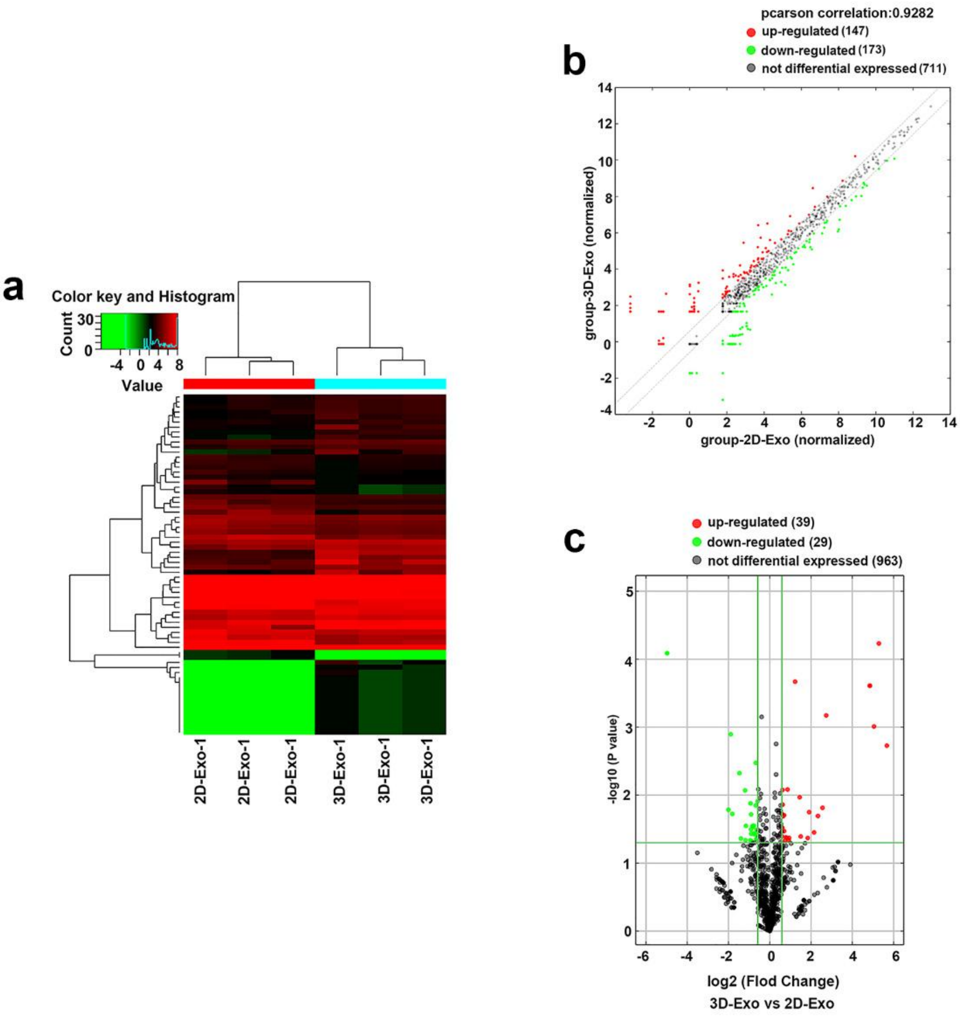

Figure S12. Microarray analysis of miRNAs between 2D-Exo and 3D-Exo.

(a) Microarray heat map representing distinct miRNA expression values in 3D-Exo compared with 2D-Exo controls (fold change≥2, P<0.05). Red color scale: higher expression. Green color scale: lower expression. n=3 samples/group. Statistical significance was evaluated by using Student's t-test and corrected for multiple testing by using the Benjamini & Hochberg method. (b) Standardization of the above image showed differentially expressed genes between the two groups. (c) Difference

analysis between 2D-Exo and 3D-Exo groups of miRNA expresion volcano map. Using log2 Fold Change (FC) > 1 and adjusted p-value < 0.01 as the screening criteria, 39 up-regulated miRNAs and 29 down-regulated miRNAs were screened.

Figure. S13

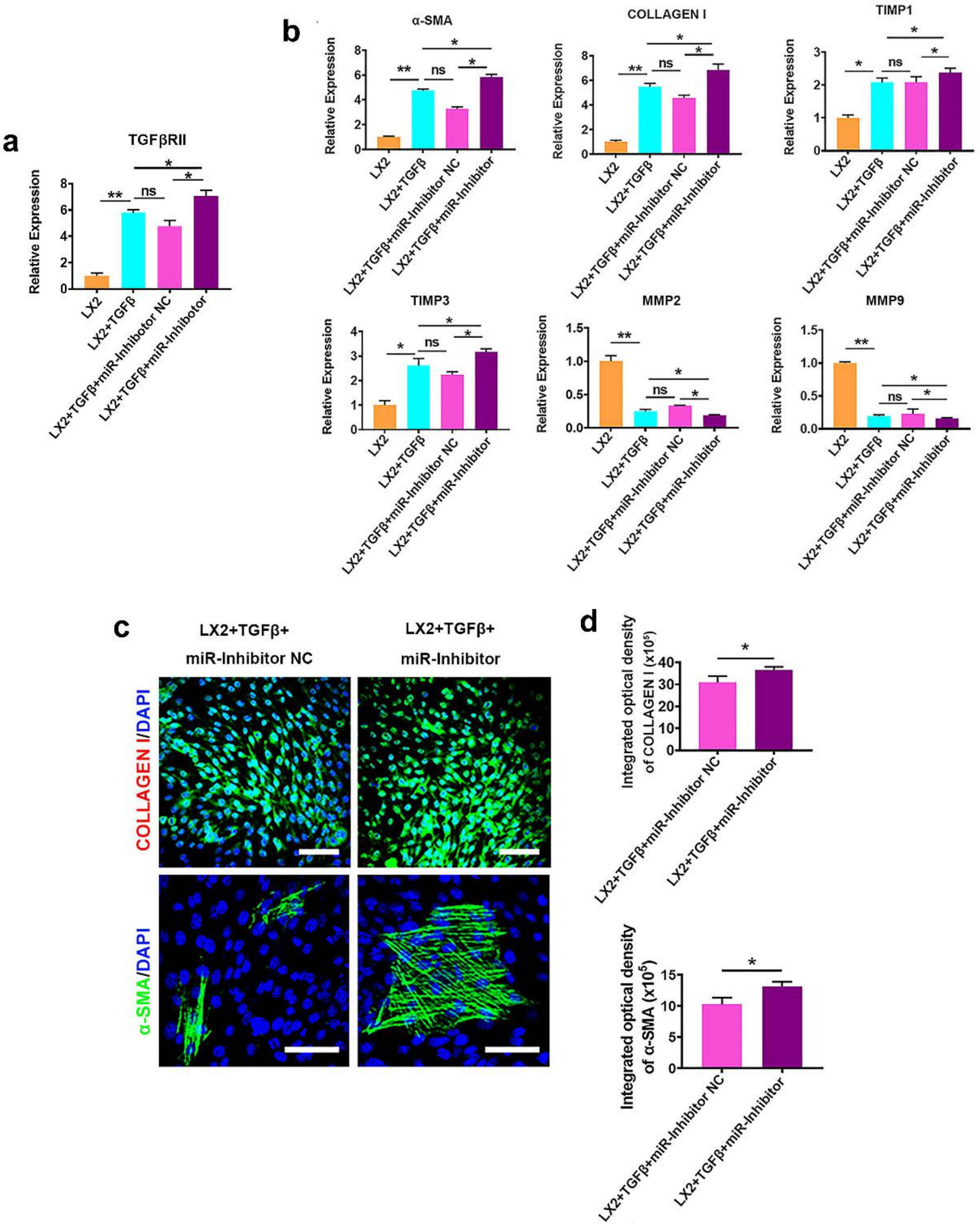

Figure S13. The knockdown of miR-6766-3p by inhibitor upregulated profibrogenic markers of activated LX2 cells.

(a) The expression level of TGFβRII genes determined by qPCR was increased in activated LX2 cells after coculture with miR-6766-3p inhibitor NC and miR-6766-3p inhibitor for 48 hours. (b) The knockdown of miR-6766-3p upregulated

profibrogenic-relatived genes (α-SMA, COLLAGEN I, TIMP1, TIMP3), and downregulated MMP2 and MMP9 in activated LX2 cells after co-culture with miR-6766-3p inhibitor NC and miR-6766-3p inhibitor for 48 hours, as determined by qPCR. (c) The positive cell number of COLLAGEN I and α-SMA was increased in activated LX2 cocultured with miR-6766-3p inhibitor for 48 hours. (d) Quantification of integrated optical density of COLLAGEN I and α-SMA in (c) showed a significant increase in miR-6766-3p inhibitor group. Magnification, 20×, 40μm; Green (COLLAGEN I; α-SMA) and DAPI (blue) were shown. n=3, Data represent the mean ± SEM. *$P < 0.05$, **$P < 0.01$, and ***$P < 0.001$.

Figure. S14

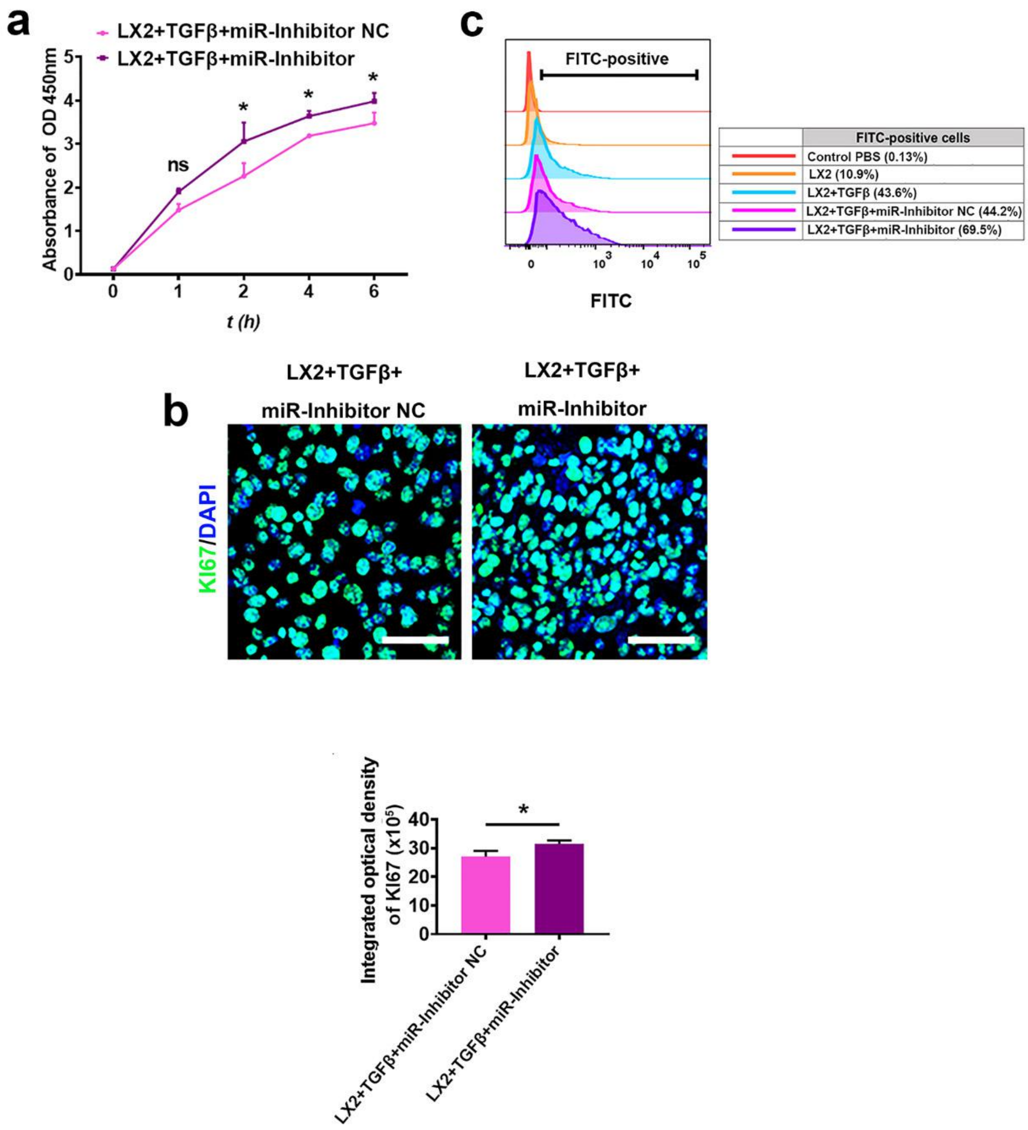

Figure S14. The knockdown of miR-6766-3p by inhibitor enhanced proliferation of LX2 cells.

(a) The inhibitory effect of miR-6766-3p on activated LX2 cell proliferation was reversed by the knockdown of miR-6766-3p with inhibitor, as measured using CCK8 assays at 2, 4 and 6 hours after exposure to miR-6766-3p inhibitor NC or miR-6766-3p inhibitor. (b) The expression of Ki67 was detected by the immunostaing

in activated LX2 cocultured with miR-6766-3p inhibitor NC and miR-6766-3p inhibitor for 48 hours. Quantification of integrated optical density of KI67 showed a significant increased by miR-6766-3p inhibitor. Magnification, 20×, 40μm; Green (KI67) and blue (DAPI) were shown. (c) Flow cytometry results showed that the increase in calcium content after TGFβ-induced activation of LX2 could be further activated by miR-6766-3p inhibitor, indicating the enhancement of activated LX2 cells by the knockdown of miR-6766-3p. n=3, Data represent the mean ± SEM. *$P < 0.05$, **$P < 0.01$, and ***$P < 0.001$.

Figure. S15

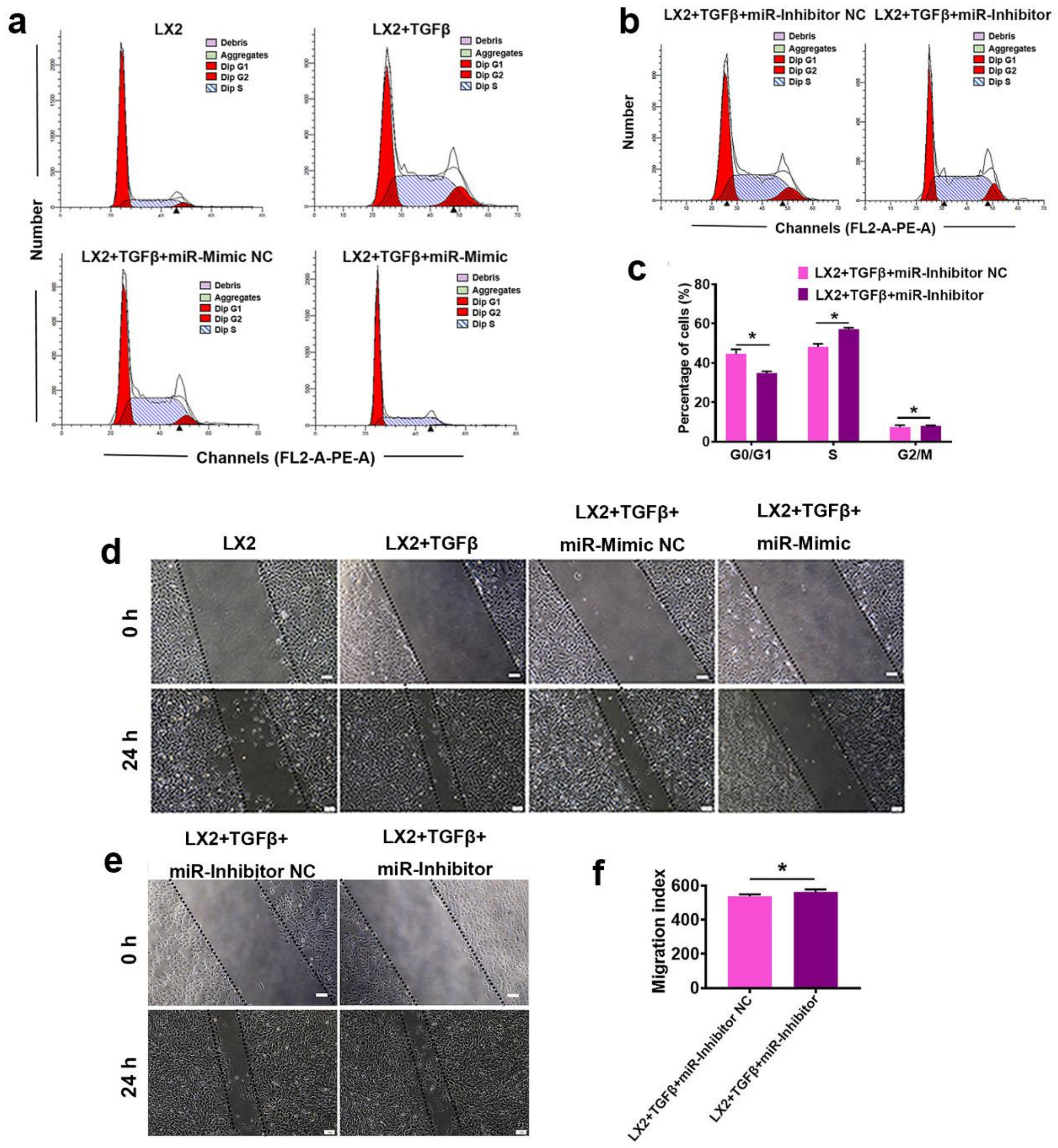

Figure S15. miR-6766-3p modulated cell cycle and migration of LX2 cells.

(a) Cell cycle analysis showed that the growth of activated LX2 cells exposed to 2D-Exo or 3D-Exo was significantly inhibited by showing lower percentage of S phase LX2 cells, when compared to the TGFβ-induced LX2 cells. (b-c) LX2 cell cycle distribution after growth in co-cultures with miR-6766-3p inhibitor NC and miR-6766-3p inhibitor for 48 hours. The percentage of S phase LX2 cells

significantly increased in miR-6766-3p inhibitor at 48 hours, indicating that the knockdown of miR-6766-3p promoted the growth of activated LX2 cells. (d) Wound recovery rates of activated LX2 cells was decreased by miR-6766-3p mimic, when compared to activated LX2 cells and the treatment group with miR-6766-3p mimic NC, as modeled by cell scratch assays (Original magnification, 4×, Scale bar, 100μm). (e-f) The decrease of wound recovery rates of active LX2 cells was inhibited by the knockdown of miR-6766-3p with the inhibitor, compared with the cells treated with miR-6766-3p inhibitor NC, as determined by cell scratch assays. n=3, Data represent the mean ± SEM. *$P < 0.05$, **$P < 0.01$, and ***$P < 0.001$.

Figure. S16

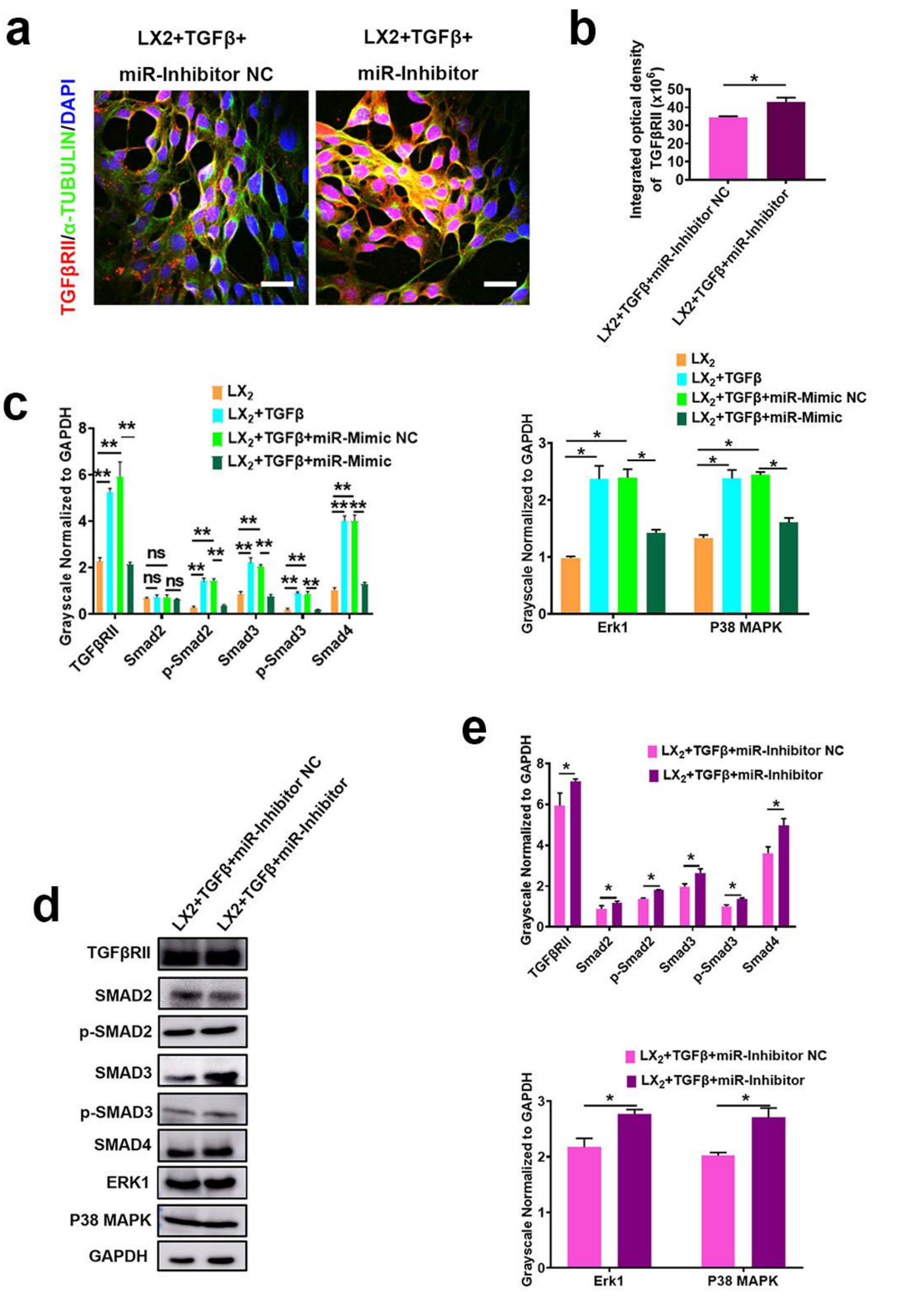

Figure S16. The knockdown of miR-6766-3p upregulated the expression of TGFβRII and activated the downstream Smads pathway.

(a) The percentage of positive cells for TGFβRII determined by immunostaining was increased in activated LX2 co-cultured with miR-6766-3p inhibitor NC and miR-6766-3p inhibitor for 48 hours, indicating that the expression of TGFβRII was upregulated by the knockdown of miR-6766-3p. (b) Quantification of integrated optical density of TGFβRII in (a) showed significantly increases in the miR-6766-3p inhibitor group. Magnification, 20×, 40 μm; Green (α-Tubulin), Red (TGFβRII) and DAPI (blue) were shown. (c) According to Western blot analysis in Fig. 8c, quantification for the expression changes of TGFβRII-mediated fibrosis pathway-associated proteins after miR-6766-3p overexpression in TGFβ treated LX2 cells. (d-e) Representative Western blot and corresponding densitometry analyses of markers, TGFβRII, p-SMAD2, SMAD3, p-SMAD3, SMAD4 and non-SMADs proteins, ERK1 and P38 MAPK in activated LX2 co-cultured with miR-6766-3p inhibitor NC and miR-6766-3p inhibitor for 48 hours. miR-6766-3p inhibitor increased the expression of the ERK1 and P38 MAPK as well as the expression of SMADs. n=3, Data represent the mean ± SEM. *P < 0.05, **P < 0.01, and ***P < 0.001.

Figure. S17

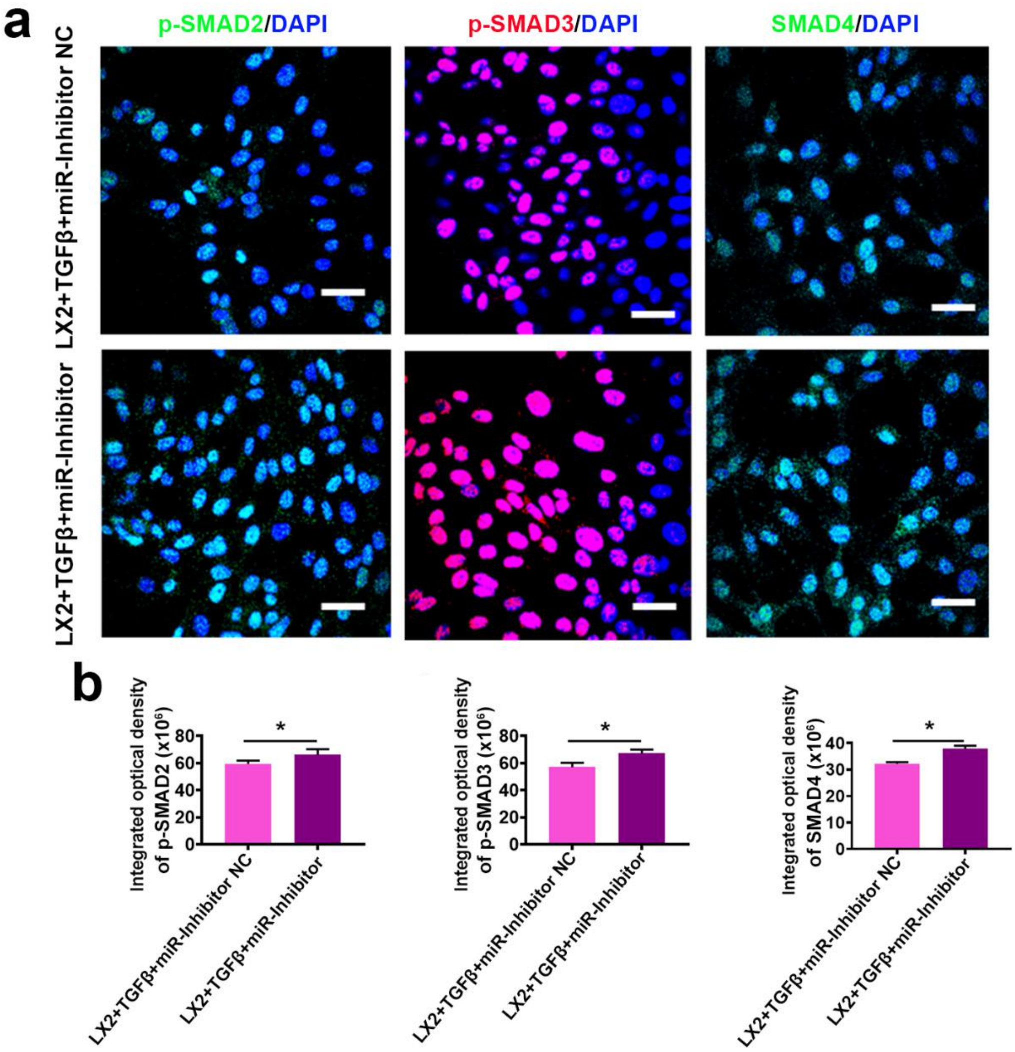

Figure S17. The knockdown of miR-6766-3p by the inhibitor activated the expression of the downstream Smads pathway.

(a) The percentage of positive nuclear staining for p-SMAD2, p-SMAD3 and SMAD4 measured by immunostaining was increased in activated LX2 cells co-cultured with miR-6766-3p inhibitor NC and miR-6766-3p inhibitor for 48 hours, indicating that the expression of SMADs were upregulated by the knockdown of miR-6766-3p with the inhibitor. (b) Quantification of integrated optical density in (a) showed that the cell

number of positive nuclear staining of p-SMAD2, p-SMAD3 and SMAD4 significantly was increased in activated LX2 cells treated with miR-6766-3p inhibitor. Magnification, 20×, 40μm; Green (p-SMAD2; SMAD4), Red (p-SMAD3) and DAPI (blue) were shown. n=3, Data represent the mean ± SEM. *$P < 0.05$, **$P < 0.01$, and ***$P < 0.001$.

Figure. S18

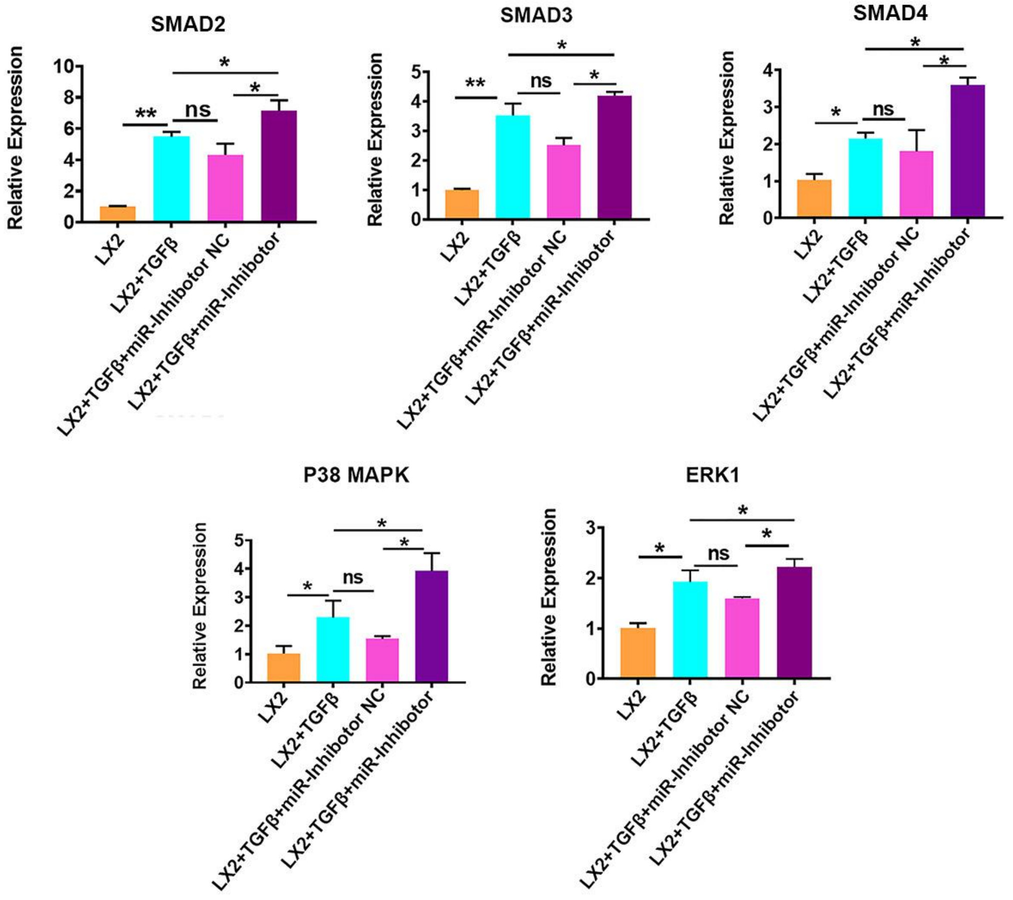

Figure S18. The inhibition of miR-6766-3p upregulated the gene expression of SMADs pathway.

The expression level of SMADs genes was increased in activated LX2 cells after co-culture with miR-6766-3p inhibitor for 48 hours when compared to those co-culture with miR-6766-3p inhibitor NC. n=3, Data represent the mean ± SEM. **P* < 0.05, ***P* < 0.01, and ****P* < 0.001.

Figure. S19

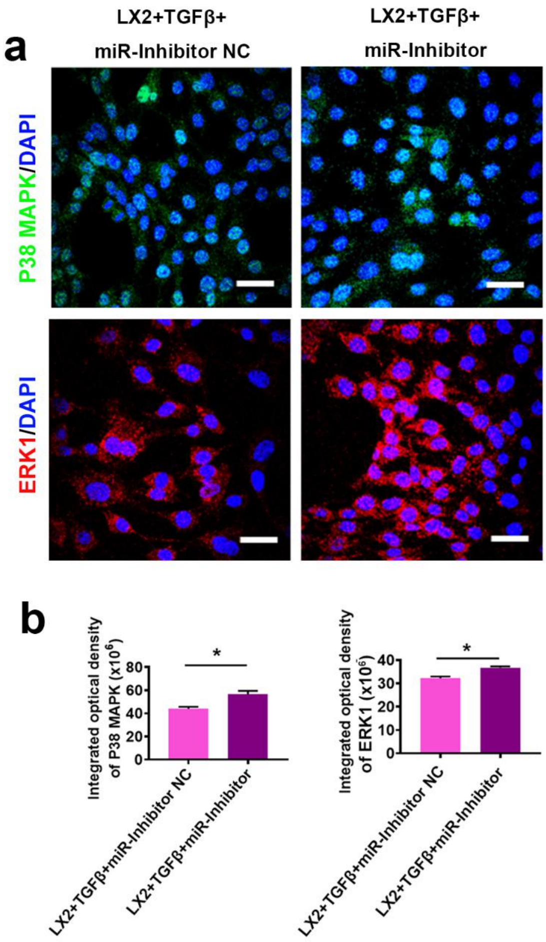

Figure S19. The knockdown of miR-6766-3p activated the expression of the upstream modulators of SMADs pathway.

(a-b) The number of positive cells for ERK1 and P38 MAPK, the upstream modulators of SMADs pathway was increased in activated LX2 cells co-cultured with miR-6766-3p inhibitor NC and miR-6766-3p inhibitor for 48 hours, as determined by immunostaining using CLSM images, indicating that miR-6766-3p inhibitor increased the expression of ERK1 and P38 MAPK, consequently leading to the expression of SMADs. (Original magnification, 20×. Scale bar, 40μm). n=3, Data represent the mean ± SEM. *P < 0.05, **P < 0.01, and ***P < 0.001.

Figure. S20

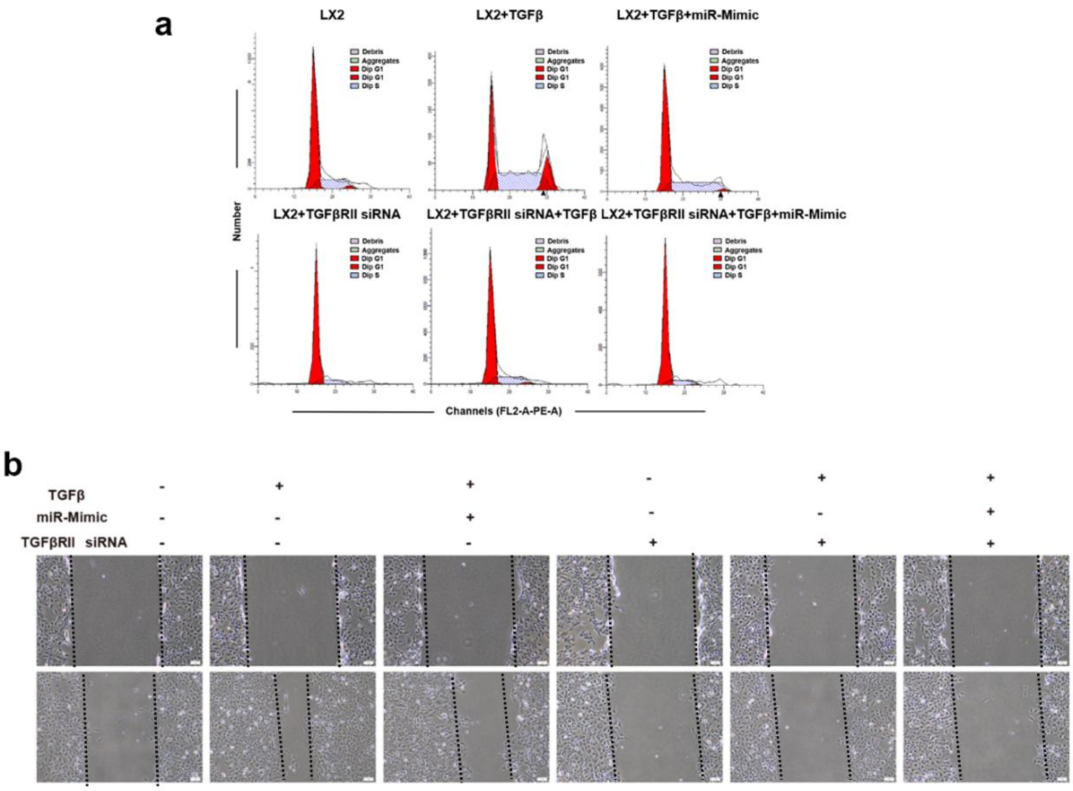

Figure S20. TGFbRII knockdown repressed proliferation and profibrogenic phenotype of LX2 cells.

(a) Cell cycle analysis showed that the growth of TGFβRII KD LX2 cells was significantly inhibited by showing lower percentage of S phase in LX2 cells. (b) Wound recovery rates of LX2 cells and TGFβRII KD LX2 cells was modeled by cell scratch assays when treated with TGFβ and/or miR-6766-3p mimic. (Original magnification, 4×, Scale bar, 100μm).

Figure. S21

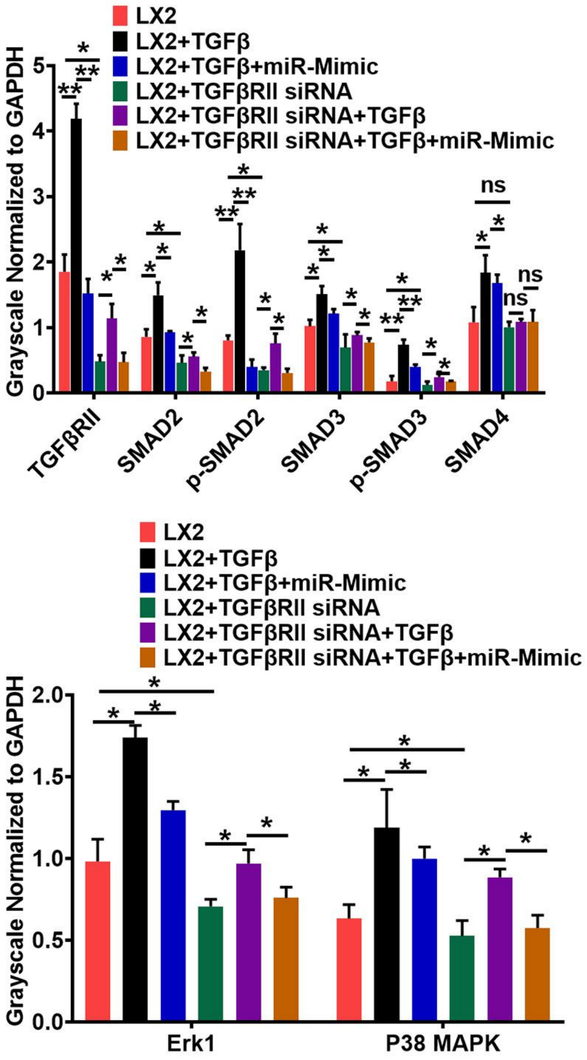

Figure S21. TGFbRII knockdown blocked the expression of downstream SMADs of TGFβ1.

According to Western blot analysis shown in Fig. 10a, the expression changes of TGFβRII/SMADs pathway-associated proteins were determined by the densitometry in LX2 cells and TGFβRII KD LX2 cells after different treatment. GAPDH was used as a loading control. n=3, Data represent the mean ± SEM. *$P < 0.05$, **$P < 0.01$, and ***$P < 0.001$.

Supporting Tables

Table S1. Primers for mRNA qPCR

Name	Sequences	Providers
h*SOX2*-qF	CGATGTACAACATGATGGAGACGGAGCT	Sangon Biotech
h*SOX2*-qR	ATTCACATGTGTGAGAGGGGCAGT	Sangon Biotech
h*OCT4*-qF	ATATGGCGGGACACCTGGCTT	Sangon Biotech
h*OCT4*-qR	CGTCAGTTTGAATGCATGGGAGAGC	Sangon Biotech
h*NANOG*-qF	ATATGAGTGTGGATCCAGCTTGTCC	Sangon Biotech
h*NANOG*-qR	CGTCACACGTCTTCAGGTTGCATGTT	Sangon Biotech
h*COLLAGEN I*-qF	ATGTCTAGGGTCTAGACATGTTCA	Sangon Biotech
h*COLLAGEN I*-qR	CCTTGCCGTTGTCGCAGACG	Sangon Biotech
h*RHOA*-qF	GGACTCGGATTCGTTGCCT	Sangon Biotech

h*RHOA*-qR	CCATCACCAACAATCACCAGTT	Sangon Biotech
h*CYCLIN D*-qF	CATCTACACCGACAACTCCATC	Sangon Biotech
h*CYCLIN D*-qR	TCTGGCATTTTGGAGAGGAAG	Sangon Biotech
h*RAC1*-qF	CCCAATACTCCTATCATCCTCG	Sangon Biotech
h*RAC1*-qR	CAGCAGGCATTTTCTCTTCC	Sangon Biotech
h*CDC42*-qF	GCCCGTGACCTGAAGGCTGTCA	Sangon Biotech
h*CDC42*-qR	TGCTTTTAGTATGATGCCGACACCA	Sangon Biotech
h*α-SMA*-qF	ACTGAGCGTGGCTATTCCTCCGTT	Sangon Biotech
h*α-SMA*-qR	GCAGTGGCCATCTCATTTTCA	Sangon Biotech
h*P27*-qF	TGCAACCGACGATTCTTCTACTCAA	Sangon Biotech
h*P27*-qR	CAAGCAGTGATGTATCTGATAAACAAGGA	Sangon

		Biotech
h*TIMP1*-qF	TTGTGGGACCTGTGGAAGTA	Sangon Biotech
h*TIMP1*-qR	CTGTTGTTGCTGTGGCTGAT	Sangon Biotech
h*MMP2*-qF	GATACCCCTTTGACGGTAAGGA	Sangon Biotech
h*MMP2*-qR	CCTTCTCCCAAGGTCCATAGC	Sangon Biotech
h*MMP9*-qF	GGGACGCAGACATCGTCATC	Sangon Biotech
h*MMP9*-qR	TCGTCATCGTCGAAATGGGC	Sangon Biotech
h*TIMP3*-qF	CTGACAGGTCGCGTCTATGA	Sangon Biotech
h*TIMP3*-qR	GGCGTAGTGTTTGGACTGGT	Sangon Biotech
h*P38 MAPK*-qF	AAGACTCGTTGGAACCCCAG	Sangon Biotech
h*P38 MAPK*-qR	TCCAGTAGGTCGACAGCCAG	Sangon Biotech

h*SMAD4*-qF	CCAATCATCCTGCTCCTGAGT	Sangon Biotech
h*SAMD4*-qR	CCAGAAGGGTCCACGTATCC	Sangon Biotech
h*TGFβRII*-qF	GTGAGAAGCCGCAGGAAGTC	Sangon Biotech
h*TGFβRII*-qR	CCGTGGTAGGTGAACTTGGG	Sangon Biotech
h*SMAD3*-qF	CATCGAGCCCCAGAGCAATA	Sangon Biotech
h*SMAD3*-qR	GTGGTTCATCTGGTGGTCACT	Sangon Biotech
h*ERK1*-qF	CCTGCGACCTTAAGATTTGTGATT	Sangon Biotech
h*ERK1*-qR	CAGGGAAGATGGGCCGGTTAGAGA	Sangon Biotech
h*SMAD2*-qF	CCGACACACCGAGATCCTAAC	Sangon Biotech
h*SMAD2*-qR	AGGAGGTGGCGTTTCTGGAAT	Sangon Biotech
h*GAPDH*-qF	GAAGATGGTGATGGGATTTC	Sangon

		Biotech
h*GAPDH*-qR	GAAGGTGAAGGTCGGAGTC	Sangon Biotech
m*Collagen I*-qF	TGACTGGAAGAGCGGAGAGT	Sangon Biotech
m*Collagen I*-qR	GACGGCTGAGTAGGGAACAC	Sangon Biotech
m*α-SMA*-qF	GAGGCACCACTGAACCCTAA	Sangon Biotech
m*α-SMA*-qR	CATCTCCAGAGTCCAGCACA	Sangon Biotech
m*Il6*-qF	GAGGATACCACTCCCAACAGACC	Sangon Biotech
m*Il6*-qR	AAGTGCATCATCGTTGTTCATACA	Sangon Biotech
m*Tgfβ*-qF	CAACAATTCCTGGCGTTACCTTGG	Sangon Biotech
m*Tgfβ*-qR	GAAAGCCCTGTATTCCGTCTCCTT	Sangon Biotech
m*Ifnγ*-qF	GGCCATCAGCAACAACATAAGCGT	Sangon Biotech

m*Ifnγ*-qR	TGGGTTGTTGACCTCAAACTTGGC	Sangon Biotech
m*Caspase 8*-qF	ATCTGGCTCGGGGTTACTG	Sangon Biotech
m*Caspase 8*-qR	CTGCGTGGTGGTCATTCTC	Sangon Biotech
m*Hgf*-qF	GAACTGCAAGCATGATGTGG	Sangon Biotech
m*Hgf*-qR	GATGCTGGAAATAGGGCAGAA	Sangon Biotech
m*Hss*-qF	GCGCAAGGCATAGAGACGA	Sangon Biotech
m*Hss*-qR	GTCCGCATCCACGACTTGAA	Sangon Biotech
m*Timp3*-qF	ACACGGAAGCCTCTGAAA	Sangon Biotech
m*Timp3*-qR	TGGAGGTCACAAAACAAGG	Sangon Biotech
m*Cyclind*-qF	TGCCATCCATGCGGAAA	Sangon Biotech
m*Cyclind*-qR	AGCGGGAAGAACTCCTCTTC	Sangon

		Biotech
m*Rac1*-qF	CGGAGCTGTTGGTAAAACCTG	Sangon Biotech
m*Rac1*-qR	ATAGGCCCAGATTCACTGGT	Sangon Biotech
m*Cdc42*-qF	TTGTTGGTGATGGTGCTGTT	Sangon Biotech
m*Cdc42*-qR	AATCCTCTTGCCCTGCAGTA	Sangon Biotech
m*P27*-qF	AAGGGCCAACAGAACAGAAG	Sangon Biotech
m*P27*-qR	GGATGTCCATTCAATGGAGTC	Sangon Biotech
m*Rhoa*-qF	CCGCCTGCGGCCTCTCTCTT	Sangon Biotech
m*Rhoa*-qR	TGGCCAACTCCCGTCTCGTGT	Sangon Biotech
m*Gapdh*-qF	TTGTCTCCTGCGACTTCAAC	Sangon Biotech
m*Gapdh*-qR	GTCATACCAGGAAATGAGCTTG	Sangon Biotech

Table S2. Primers for miRNA qPCR

Name	Sequences	Providers
hsa-miR-4728-5p-qF	GTCGTATCCAGTGCAGGGTCCGAGGT	Sangon
	ATTCGCACTGGATACGACTGCTTG	Biotech
hsa-miR-6734-5p-	GTCGTATCCAGTGCAGGGTCCGAGGT	Sangon
qF	ATTCGCACTGGATACGACTCTCCA	Biotech
hsa-miR-3652- qF	GTCGTATCCAGTGCAGGGTCCGAGGT	Sangon
	ATTCGCACTGGATACGACTCCTCA	Biotech
hsa-miR-6793-5p-	GTCGTATCCAGTGCAGGGTCCGAGGT	Sangon
qF	ATTCGCACTGGATACGACTCACCC	Biotech
hsa-miR-6766-3p-	GTCGTATCCAGTGCAGGGTCCGAGGT	Sangon
qF	ATTCGCACTGGATACGACTGAGGG	Biotech
hsa-miR-6785-5p-	GTCGTATCCAGTGCAGGGTCCGAGGT	Sangon
qF	ATTCGCACTGGATACGACCACCAT	Biotech
hsa-miR-500a-5p-	GTCGTATCCAGTGCAGGGTCCGAGGT	Sangon
qF	ATTCGCACTGGATACGACTCTCAC	Biotech
hsa-miR-6511b-5p-	GTCGTATCCAGTGCAGGGTCCGAGGT	Sangon
qF	ATTCGCACTGGATACGACTGTCAG	Biotech
hsa-miR-4690-5p-	GTCGTATCCAGTGCAGGGTCCGAGGT	Sangon
qF	ATTCGCACTGGATACGACTTCAGC	Biotech

hsa-miR-5194- qF	GTCGTATCCAGTGCAGGGTCCGAGGT ATTCGCACTGGATACGACCCATCC	Sangon Biotech
hsa-miR-4259- qF	GTCGTATCCAGTGCAGGGTCCGAGGT ATTCGCACTGGATACGACTCCTGA	Sangon Biotech
U6-qF	AAAATATGGAACGCTTCACGAATTTG	Sangon Biotech
hsa-miR-qR	AGTGCAGGGTCCGAGGTATT	Sangon Biotech

Table S3. Primers for miRNA PCR

Name	Sequences	Providers
hsa-miR-4728-5p-F	TGGGAGGGGAGAGGCAG	Sangon Biotech
hsa-miR-6734-5p-F	GCGTTGAGGGGAGAATGAGG	Sangon Biotech
hsa-miR-3652-F	CGCGCGGCTGGAGGTG	Sangon Biotech
hsa-miR-6793-5p-F	GCGTGTGGGTTCTGGGTTG	Sangon Biotech
hsa-miR-6766-3p-F	GCGTGATTGTCTTCCCCCA	Sangon Biotech
hsa-miR-6785-5p-F	TGGGAGGGCGTGGATG	Sangon Biotech
hsa-miR-500a-5p-F	CGCGTAATCCTTGCTACCTGG	Sangon Biotech
hsa-miR-6511b-5p-F	CTGCAGGCAGAAGTGGGG	Sangon Biotech
hsa-miR-4690-5p-F	GGAGCAGGCGAGGCTGG	Sangon Biotech
hsa-miR-5194-F	GCGTGAGGGGTTTGGAATG	Sangon Biotech
hsa-miR-4259-F	CGCAGTTGGGTCTAGGGG	Sangon Biotech
U6-F	CTCGCTTCGGCAGCACATATACT	Sangon Biotech
hsa-miR-R	ACGCTTCACGAATTTGCGTGTC	Sangon Biotech

Table S4. Antibodies used in this study

Name	Species reactivity	Applications	Providers	Cat. No.
anti-CD9	Human, Mouse, Rat	WB (1:500)	Bioss	bs-2489R
anti-CD63	Human	WB (1:500)	Bioss	bs-1523R
anti-α-SMA	Human, Mouse, Rat	WB (1:1000); IF (1:200)	CST	48938S
anti-collagen I	Human, Mouse, Rat, Rabbit	WB (1:500); IF (1:100)	Bioss	bs-10423R
anti-CDC42	Mouse, Rat, Human	WB (1:10000); IF (1:500)	Abcam	ab187643
anti-TGF beta1	Mouse, Rat, Human	WB (1:500); IF (1:100)	Bioss	bs-0086R
anti-RhoA	Mouse, Rat, Human	WB (1:5000); IF (1:150)	Abcam	ab187027
anti-GAPDH	Mouse, Rat, Human	WB (1:1000)	CST	5174s
anti-F4/80	Mouse	IHC (1:400)	CST	30325T

anti-iNOS	Mouse	IHC (1:400)	CST	13120S
anti-TNFα	Mouse	IHC (1:400)	CST	11948T
anti-PCNA	Mouse, Rat, Human	IHC (1:25)	Abcepta	AW5198
anti-ALB	Human, Mouse	WB (1:1000)	Abcepta	AP6540B
anti-PCK1	Mouse, Rat, Human	WB (1:1000)	Abcam	ab28455
anti-caspase 8	Human, Mouse	WB (1:2000)	Bioss	bsm-33190M
anti-CD68	Mouse, Rat, Human	WB (1:1000)	Abcam	ab955
anti-Desmin	Mouse, Rat, Human	IF (1:400)	CST	5332
anti-CD206	Mouse, Rat, Human	WB (1:1000)	Abcam	ab64693
anti-MCP1	Mouse, Rat, Human	WB (1:1000)	Novus	NBP1-07035SS
anti-Ki67	Human	IF (1:400)	CST	9449
anti-TGFβRII	Mouse, Rat, Human	WB (1:500)	Bioss	bs-0117R

anti-α-Tubulin	Mouse, Rat, Human	IF (1:1000)	CST	3873s
anti-p-SMAD2	Mouse, Rat, Human	WB (1:1000)	CST	18338T
anti-p-SMAD3	Mouse, Human	WB (1:2000)	Abcam	Ab52903
anti-SMAD4	Mouse, Rat, Human	WB (1:1000); IF (1:800)	CST	46535T
anti-SMAD2	Mouse, Rat, Human	WB (1:1000); IF (1:100)	CST	5339T
anti-SMAD3	Mouse, Rat, Human	WB (1:1000); IF (1:100)	CST	9523T
anti-ERK1	Mouse, Human	WB (1:1000); IF (1:400)	Abcam	Ab32537
anti-P38 MAPK	Mouse, Rat, Human	WB (1:1000); IF (1:200)	CST	8690T
anti-SOX2	Mouse, Human	IF (1:400)	CST	4900
anti-OCT4	Human	IF (1:200)	CST	2750
PE Anti-OCT4	Human	FlowCytometry (5ul/Test)	STEM CEL	60093PE

			L	
PE Anti-SSEA-4	Human	FlowCytometry (5ul/Test)	STEMCELL	60062PE
PE Anti-TRA-1-81	Human	FlowCytometry (5ul/Test)	STEMCELL	60065PE
PE-Cy™5 Mouse IgG1 κ Isotype Control	Human	FlowCytometry (20ul/Test)	BD	555750
Anti-mouse IgG (H+L) F(ab')2 Fragment (Alexa Fluor 594 Conjugate)	mouse	IF (1:1000)	CST	8890S
Anti-mouse	mouse	IF (1:1000)	CST	4408s

IgG (H+L)F(ab') 2 Fragment (Alexa Fluor 488 Conjugate)				
Anti-rabbit IgG (H+L) F(ab')2 Fragment (Alexa Fluor 594 Conjugate)	rabbit	IF (1:1000)	CST	8889s
Anti-rabbit IgG (H+L)F(ab') 2 Fragment (Alexa Fluor 488 Conjugate)	rabbit	IF (1:1000)	CST	4412s
Anti-rabbit	rabbit	WB (1:1000)	CST	7074s

IgG				
HRP-linked				
Antibody				
Anti-mouse IgG HRP-linked Antibody	mouse	WB (1:1000)	CST	7076S
